# Multidirectional Charge Separation in Self‐Assembled Aggregates of Perylenebisimide‐Porphyrin Bola‐Supra‐Amphiphiles

**DOI:** 10.1002/anie.202523324

**Published:** 2026-01-05

**Authors:** Erik J. Schulze, Elena A. Mack, Christian L. Ritterhoff, Ufuk Borucu, Bernd Meyer, Dirk M. Guldi, Andreas Hirsch

**Affiliations:** ^1^ Department of Chemistry and Pharmacy Chair of Organic Chemistry II Friedrich‐Alexander‐Universität Erlangen‐Nürnberg Nikolaus‐Fiebiger‐Straße 10 91058 Erlangen Germany; ^2^ Department of Chemistry and Pharmacy Chair of Physical Chemistry I Friedrich‐Alexander‐Universität Erlangen‐Nürnberg Egerlandstraße 3 91058 Erlangen Germany; ^3^ Interdisciplinary Center for Molecular Materials (ICMM) and Computer Chemistry Center (CCC) Friedrich‐Alexander‐Universität Erlangen‐Nürnberg Nägelsbachstraße 25 91052 Erlangen Germany; ^4^ GW4 Facility for High‐Resolution Electron Cryo‐Microscopy University of Bristol 24 Tyndall Avenue Bristol UK

**Keywords:** Charge Transfer, Donor‐Acceptor Systems, Hydrogen Bonds, Self‐Assembly, Supra‐Amphiphiles

## Abstract

We report on the synthesis of a hydrogen‐bond mediated bola‐type supra‐amphiphile and assembly thereof in water. The assembly is based on amphiphilic porphyrins and hydrophobic perylenebisimdes (PBI), which both form the resulting bola‐form solely through the H‐bonding motif, which is shielded by the assembly of the chromophores itself. The amphiphilic porphyrin was functionalized, on one hand, with a cyanuric acid and, on the other hand, with an oligo carboxylate dendron as polar head group. Two Hamilton receptors were linked to the PBI on each imide position. Assembly was achieved by lyophilizing solutions of both components in water/THF mixtures, followed by redispersion in pure water yielding stable suspensions. Cryogenic transmission electron microscopy (cryo‐TEM) and dynamic light scattering (DLS) reveal a spherical morphology with diameters ranging from 15 – 100 nm. Once formed, the assemblies showed broadened absorptions and quenched PBI‐centered fluorescence. Using time‐resolved absorption spectroscopy, the nature of the fluorescence quenching was confirmed to be either charge separation, by which the porphyrin donates an electron to the PBI, or symmetry breaking charge separation, by which π‐π stacked PBIs donate and accept electrons. Denaturation of the supra‐amphiphile went hand‐in‐hand with a reinstation of the PBI fluorescence and suppression of charge separation.

## Graphical abstract

Stable aggregates of a new hydrogen‐bond mediated porphyrin‐perylenebisimide (PBI) bola‐type supra‐amphiphile have been synthesized by lyophilization of solutions of both single molecules in THF/water mixtures, followed by redispersion in pure water. Cryogenic transmission electron microscopy verifies the spherical morphology. Femtosecond transient absorption spectroscopy unravels multi‐directional charge separation from the porphyrin to the PBI and within the PBI stacks.

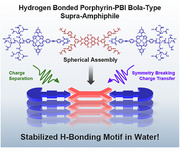

## Introduction

In the pursuit of synthetically mimicking photosynthetic systems, gaining precise control over the self‐assembly of multiple organic chromophores into hierarchical, highly organized structures presents a substantial challenge.^[^ ^1^, ^2^, ^3^ ^]^ Particularly enticing is hereby the possibility of assembling and ordering electron donor‐acceptor (D–A) pairs in water, capable of directed charge transfer (CT) interactions in their excited state. Such systems are attractive, as they not only resemble natural light harvesting processes, but also open the door for new optoelectronic materials,^[^ ^1^, ^4^, ^5^, ^6^ ^]^ notably for organic photovoltaics,^[^ ^7^, ^8^ ^]^ and photocatalysis.^[^ ^9^ ^]^ A especially powerful concept to realize large‐scale self‐organized chromophore systems lies in leveraging amphiphilic structures, while hydrogen bonding (H‐bonding) motifs stand out as being able to closely imitate covalent D‐A couples in their structure and function. Combining these two concepts is challenging as they are often mutually exclusive. Consequently, the stabilization of a directed H‐bonding motif between amphiphilic D‐A pairs would mark a significant step change towards self‐organizing aggregates in water that are subject to complex photophysics.

**Scheme 1:**
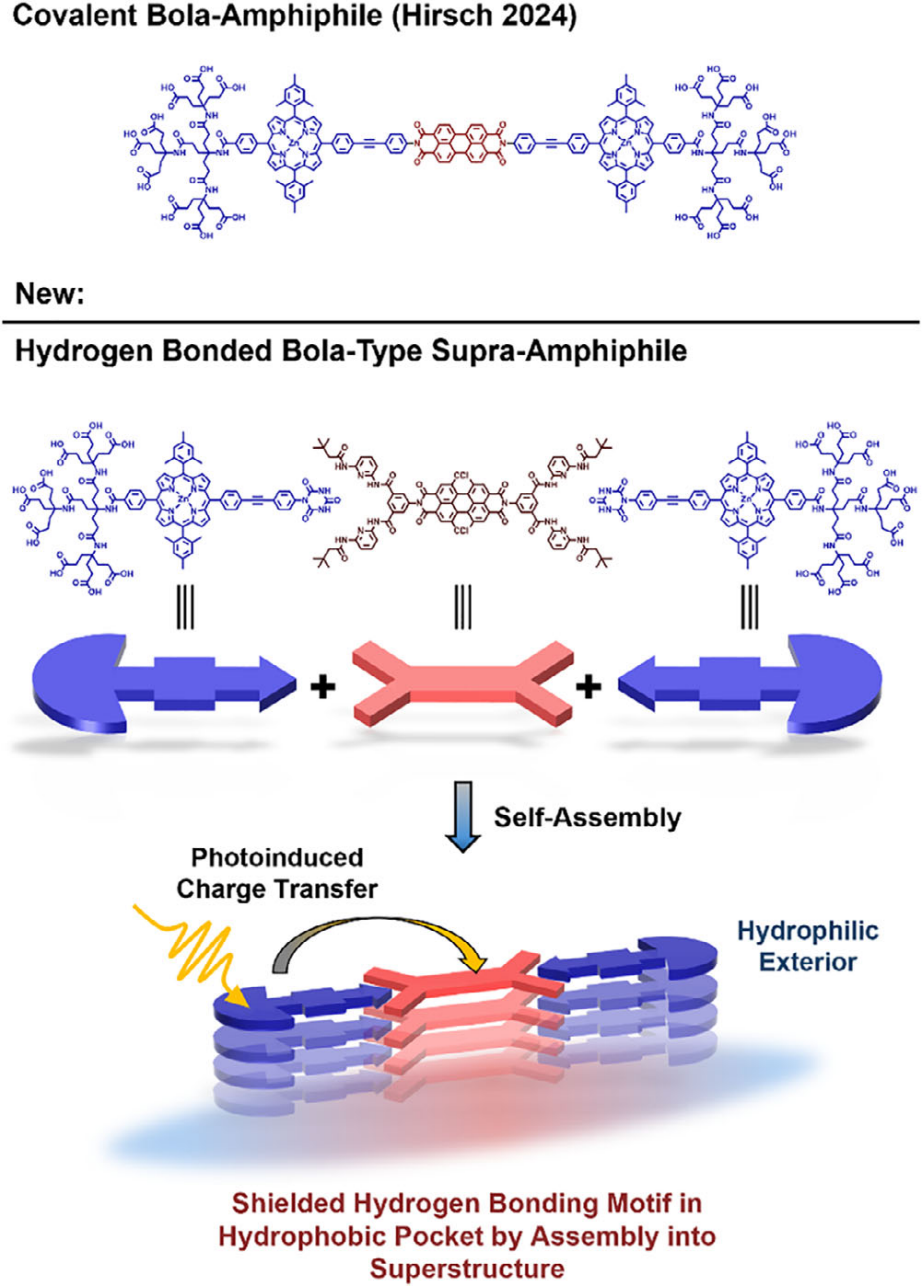
Schematic representation of the individual building blocks, that is, **CyPor** and **HamPBI**, and a simplified illustration of their assembly into a bola‐type supra‐amphiphile in water.

Generally, the design principles of amphiphiles have been already applied to multiple large photoactive molecules, such as porphyrins,^[^ ^9^, ^10^ ^]^ BODIPYs,^[^ ^11^, ^12^, ^13^ ^]^ HBCs,^[^ ^14^, ^15^ ^]^ perylenebisimides (PBI),^[^ ^16^, ^17^, ^18^, ^19^ ^]^ and fullerenes,^[^ ^20^, ^21^, ^22^ ^]^ resulting in highly organized supramolecular assemblies. In contrast, due to their often‐challenging synthesis, covalent electron D‐A systems that are amphiphilic and that reveal the desired directional CT interactions have only been scantly explored.^[^ ^10^, ^23^ ^]^ Such systems are highly attractive nonetheless, as the segregated stacking motifs support long‐lived charge‐separated (CS) states, countering a fast charge recombination in alternating stacks.^[^ ^23^, ^24^ ^]^ To this end, we recently synthesized covalent D–A amphiphiles on the basis of the family of porphyrin‐PBI conjugates^[^ ^25^, ^26^, ^27^ ^]^ and probed their self‐assembly in water (Scheme 1).^[^ ^28^, ^29^ ^]^

By seeking to expand the scope of covalent amphiphilic structures, the field of supra‐amphiphiles emerged, assembling the individual amphiphile itself via supramolecular interactions.^[^ ^30^, ^31^ ^]^ Supra‐amphiphiles exhibit behavior akin to their covalent counterparts, spontaneously organizing as complete units into larger, ordered structures.^[^ ^31^, ^32^, ^33^ ^]^ To date, outstanding supra‐amphiphiles have been successfully developed, focusing mostly on macrocyclic host‐guest systems such as pillararenes, calixarenes, or calixpyrroles combined with numerous guests,^[^ ^34^, ^35^, ^36^, ^37^ ^]^ π–π interactions,^[^ ^38^ ^]^ or charge‐transfer interactions.^[^ ^39^ ^]^ These interactions are however, far from being ideal in the context of mimicking linear D‐A systems without compromising their photophysical functionality. H‐bond motifs are often only prominent as cooperative secondary interactions and are rarely employed as the primary non‐covalent force due to their highly dynamic nature, especially in aqueous environments.^[^ ^40^, ^41^ ^]^

Therefore, when seeking to implement H‐bonds as the primary interaction, a highly directional H‐bonding motif is imperative, where the Hamilton receptor (**Ham**) – cyanuric acid (**Cy**) couple stands out with its excellent synergy. Its most notable features are its strong cooperative binding and its synthetic versatility.^[^ ^42^ ^]^ This motif has already been established in light harvesting systems,^[^ ^43^, ^44^, ^45^, ^46^ ^]^ functionalized nanoparticles,^[^ ^47^, ^48^ ^]^ supramolecular polymers,^[^ ^42^ ^]^ and graphene functionalization.^[^ ^49^ ^]^ An additional requirement to enable the functional H‐bonding is the balance between the spatial distance of the terminal polar head groups and the binding motif, as it is decisive for the formation of the hydrophobic pocket and enabling effective excited state communication.

In this work, we report on the design, synthesis, and assembly of a first example of a porphyrin‐PBI bola‐type supra‐amphiphile around a shielded **Ham**‐**Cy** motif in water (Scheme 1) that is capable of photoinduced charge separation processes. Notably, we established the bola‐form solely through a shielded H‐bonding motif, where stacks of the supra‐amphiphile itself provide a preorganized hydrophobic pocket. To the best of our knowledge, this type of stabilization without compromising its inherent CT activity is unprecedented. To this end, it features an amphiphilic cyanuric acid zinc porphyrin (**CyPor**) and a hydrophobic *bis*‐Hamilton receptor PBI (**HamPBI**). For improved synthetic accessibility and solubility, the Hamilton receptor was placed at the PBI. A diphenylacetylene (tolane) and a Newkome G2 oligo carboxylate were chosen as spacer and terminal head group, respectively. Spacer and dendrimer provide the optimum electron D‐A distance, notably without compromising the overall water‐solubility. The assemblies were found to be stable in aqueous solutions. The most important pieces of evidence came from cryo‐TEM imaging, which revealed their assembly into spherical aggregates, and from femtosecond transient absorption spectroscopy (fsTAS), which corroborated the formation of a charge separated state following photoexcitation and charge separation (CS) or symmetry breaking charge separation (SB‐CS). Our porphyrin‐PBI bola‐type supra‐amphiphile serves as a first prime example of a new class of supra‐amphiphiles, which easily assemble, and, which closely resemble covalent D‐A counterparts. The versatility of the hydrogen‐bonding system enables easy modular integration with various chromophores. This concept marks a meaningful advancement in the development of artificial photosynthetic systems, serving as an initial model that mimics key features of the complex, multi‐layered supramolecular interactions seen in natural photosynthesis.

## Results and Discussion

### Synthesis

**Scheme 2:**
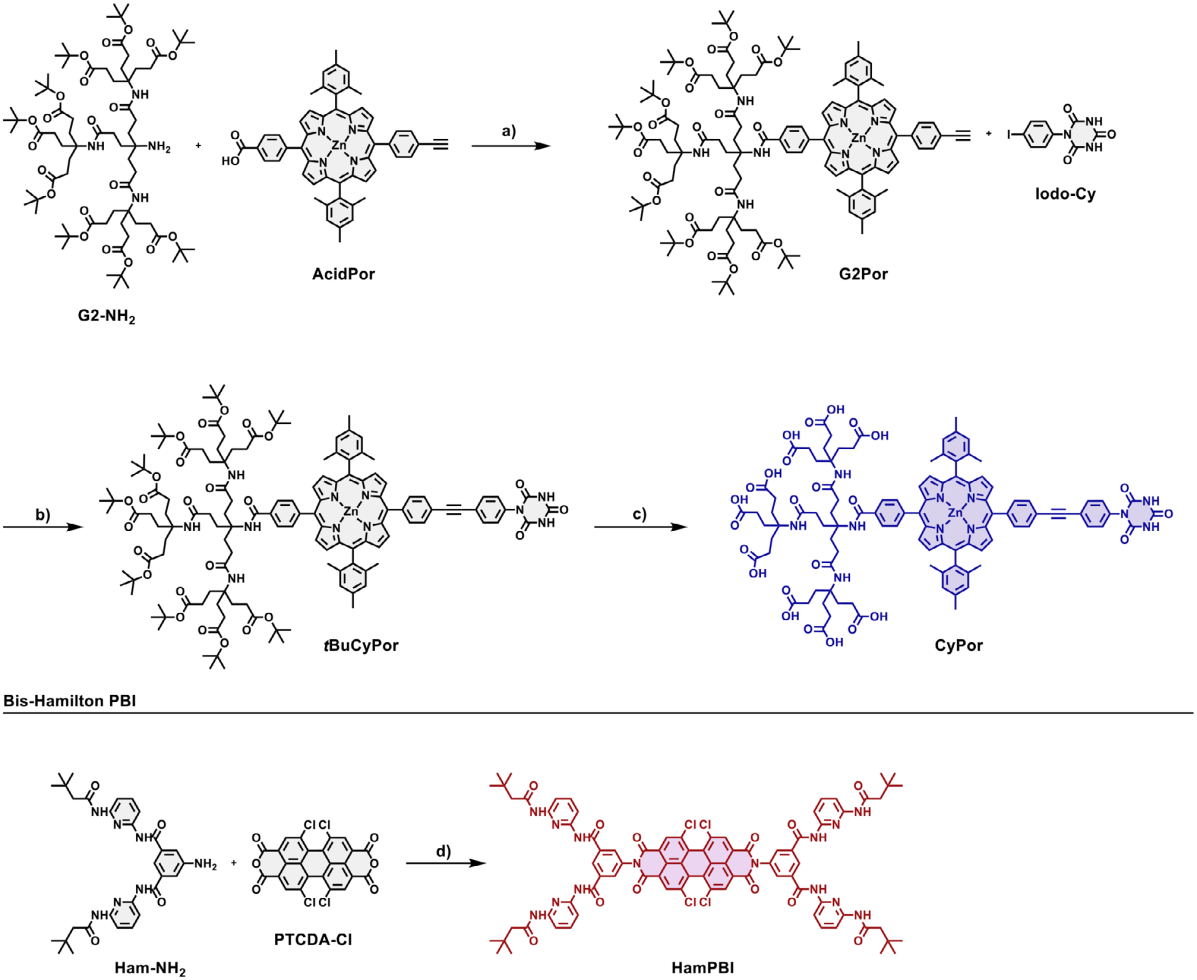
a) HOBt•H_2_O, DCC, DMF, rt., 3 d, 58 %; b) Pd(PPh_3_)_4_, PPh_3_, CuI, THF/NEt_3_ 2:1 (v/v), 80 °C, 16 h, 72%; c) HCOOH, rt., 3 d, 65%; d) Zn(OAc)_2_, pyridine, 120 °C, 7 d, 46%.

The synthetic route towards the desired porphyrin amphiphile **CyPor** is depicted in Scheme 2. To this end, the amide coupling of carboxylic acid porphyrin **AcidPor** with Newkome dendron **G2‐NH_2_** using HOBt•H_2_O and DCC in DMF gave the dendronized porphyrin **G2Por** in 58% yield. The linkage of the 4‐iodo‐phenyl cyanuric acid (**Iodo‐Cy**) and **G2Por** was achieved by Sonogashira cross coupling using Pd(PPh_3_)_4_ with CuI and additional PPh_3_ in THF/NEt_3_ and afforded ***t*BuCyPor** in 72% yields. The solubility of ***t*BuCyPor** was limited in chlorinated solvents like dichloromethane, but good in THF. ***t*BuCyPor** was fully characterized by NMR spectroscopy and high‐resolution mass spectrometry (Supporting Information). Cleavage of the *t*Bu‐esters of the dendrons to obtain the oligo carboxylic acids was carried out by stirring in neat formic acid at room temperature for 3 days. Despite the clean conversion towards the carboxylic acids, a drawback of this method is the demetallation of the porphyrin. Therefore, re‐metalation with additional Zn(OAc)_2_ was necessary to isolate the desired amphiphile **CyPor** in 65% yield. Successful reinsertion of zinc was monitored by tracking the characteristic features of the free base porphyrin in the absorption spectra. **CyPor** is insoluble in pure organic solvents, including DMSO, DMF, and HFIP. **CyPor** shows, however, good solubility in basic aqueous solutions and acidified solutions of THF. Further addition of THF to basic aqueous solutions enhances the solubility. By dissolving **CyPor** in water with the aid of 9 eq. NaOH, followed by removal of the solvent, the sodium salt of **CyPor** was obtained as a highly water‐soluble dark purple powder. Cyanuric acid amphiphile **CyPor** was unambiguously characterized by high‐resolution mass spectrometry (HRMS) as well as NMR and ATR‐IR spectroscopy (Supporting Information). ^1^H NMR of **CyPor** in basic D_2_O gave broad signals due to aggregation at this concentration, even at elevated temperatures (Figure S46). However, NMR spectra recorded in D_2_O / THF‐*d_8_* mixtures gave reasonably resolved spectra (Figure S50). Here, the absence of the signals stemming form *t*Bu‐signals in ^1^H NMR and ^13^C NMR corroborated the complete deprotection of the dendron. HRMS spectrum of **CyPor** was obtained by electrospray ionization (ESI) in negative mode and confirmed the successful synthesis (Figures S61–S64). The main signal corresponds to the doubly negatively charged amphiphile [M‐2H]^2−^ at 972.8241 m/z (calc. 972.8214 m/z). Additionally, higher charged signals between [M‐3H]^3−^ and [M‐H]^−^ are also observed.

To synthesize the complementary *bis*‐Hamilton receptor PBI (**HamPBI**), imidization of a commercially available *tetra* chloro‐PBI (**PTCDA‐Cl**) and a literature‐known amino‐substituted Hamilton receptor (**Ham‐NH_2_**) was carried out (Scheme 2). **PTCDA‐Cl** was chosen, as the absorption of the corresponding PBI poorly overlaps with the porphyrin Q‐band absorption of **CyPor**. It exhibits also higher solubility compared to the unsubstituted **PTCDA**. Using molten imidazole at a variety of temperatures, as well as using Zn(OAc)_2_ as a catalyst, did not afford the desired product. However, by changing the solvent to pyridine and the temperature to 110 °C together with Zn(OAc)_2_ we isolated **HamPBI** in 46% yield. The reaction proceeds slowly and takes 7 days to reach maximum conversion. Longer reaction times, that is, of up to 14 days, as well as higher reaction temperatures, that is, of up to 130 °C, did not improve the yield significantly. Instead, more side products were observed. **HamPBI** was fully characterized by NMR and ATR‐IR spectroscopy as well as HRMS (Supporting Information).

### Theoretical Investigations

**Figure 1:**
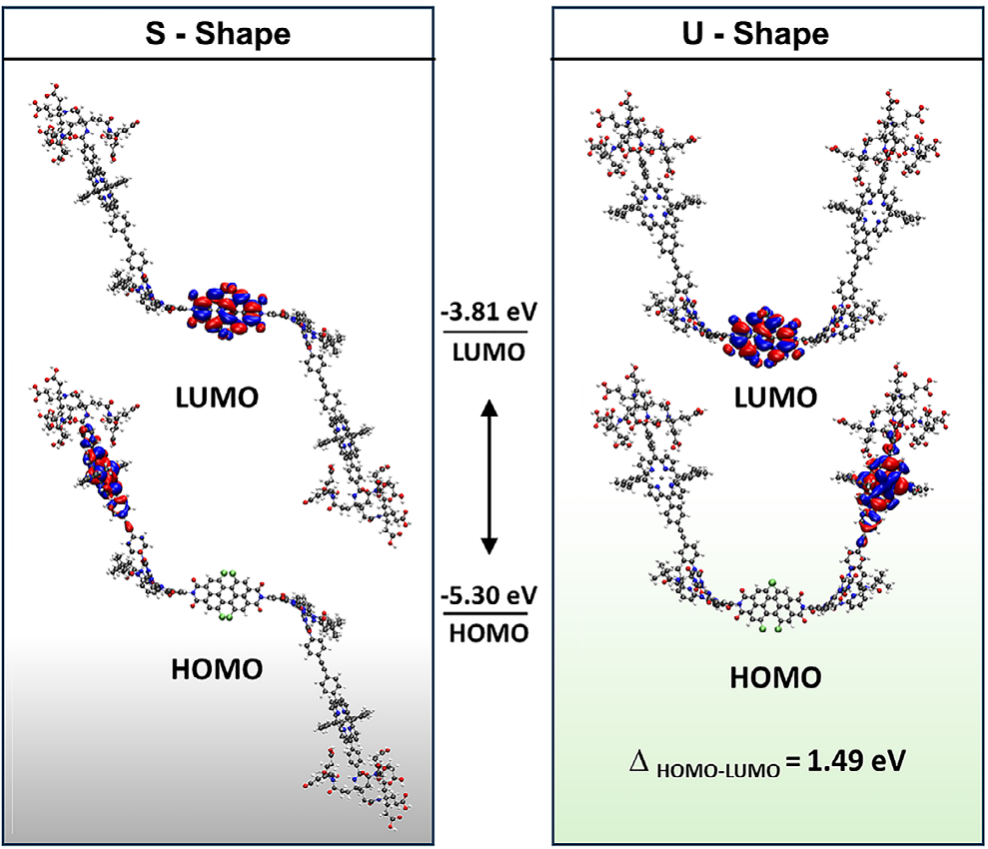
Geometry‐optimized structure and orbital contours of **HamPBI(CyPor)_2_** in the gas phase, calculated by DFT at the B3LYP/def2‐TZVPP level of theory including an implicit solvation model. The eigenvalues of the frontier orbitals as well as their energy gap are also denoted. As a result of the symmetry of the assembly, the HOMO is degenerate and could also be drawn on the other **CyPor** moiety.

Density functional theory (DFT) calculations were performed to elucidate the structure and electronic properties of the envisioned supra‐amphiphile. Geometry optimizations reveal a bent structure due to the steric hindrance of the hydrogen atoms of the receptor (Figure 1), which is in accordance with similar assemblies reported in the literature.^[^ ^50^ ^]^ This inherent bending leads to S‐ or U‐shaped assemblies with similar ground state energies and electronic properties (Tables S1 and S2). Interconversion between the two configurations is expected to possess at a low energy barrier and results, in turn, in a geometrically flexible system. Analysis of the electronic structure of **HamPBI(CyPor)_2_** as well as the **HamPBI** and **CyPor** building blocks document the successful separation of the electronic states in the supramolecular triad. The orbital contours (Figures S2–S5) and their respective energies (Table S1) reveal that the molecular orbitals of the triad are perfect superpositions of the building block orbitals, with the HOMO stemming from either of the two **CyPor** and the HOMO from **HamPBI** (Figure 1). Consequently, the HOMO‐LUMO transition in the triad is significantly lowered from 2.43 eV for **HamPBI** and 2.68 eV for **CyPor** to 1.49 eV for the **HamPBI(CyPor)_2_** supra‐amphiphile.

Additional TD‐DFT calculations were performed to probe changes in the absorption spectra due to the assembly of the building blocks. The calculated spectra as well as the electronic transitions are detailed in the Supporting Information. Similar as observed for the orbital contours, little to no differences were found between the spectrum of the assembly and the combination of the spectra of the two individual components. Therefore, it can be concluded that **HamPBI(CyPor)_2_** features the characteristics of the envisioned supra‐amphiphile, namely an electronic separation suitable for electron transfer, together with a geometric shape capable of forming the desired aggregates.

### Supramolecular Assembly

To gain first insights into the nature of the H‐bonding motif, isothermal titration calorimetry (ITC) measurements were carried out using **HamPBI** and ***t*BuCyPor**. Here, a 2 mM solution of ***t*BuCyPor** in CHCl_3_ was titrated into a 0.1 mM CHCl_3_ solution of **HamPBI** at 25 °C (Figure S7). To account for the dilution and possible disaggregation heats, reference measurements of titrating ***t*BuCyPor** in blank chloroform were carried out and subtracted from the obtained titration data. Global analysis using a 2:1 binding model of three subsequent titrations gives association constants of *k*_ass1_ = (9.00 ± 0.23) × 10^4^ M^−1^ and *k*_ass1 _ =  (4.08 ± 0.20) × 10^3^ M^−1^ (Table S10), which is well in line with literature reports on similar systems.^[^ ^51^ ^]^

**Figure 2:**
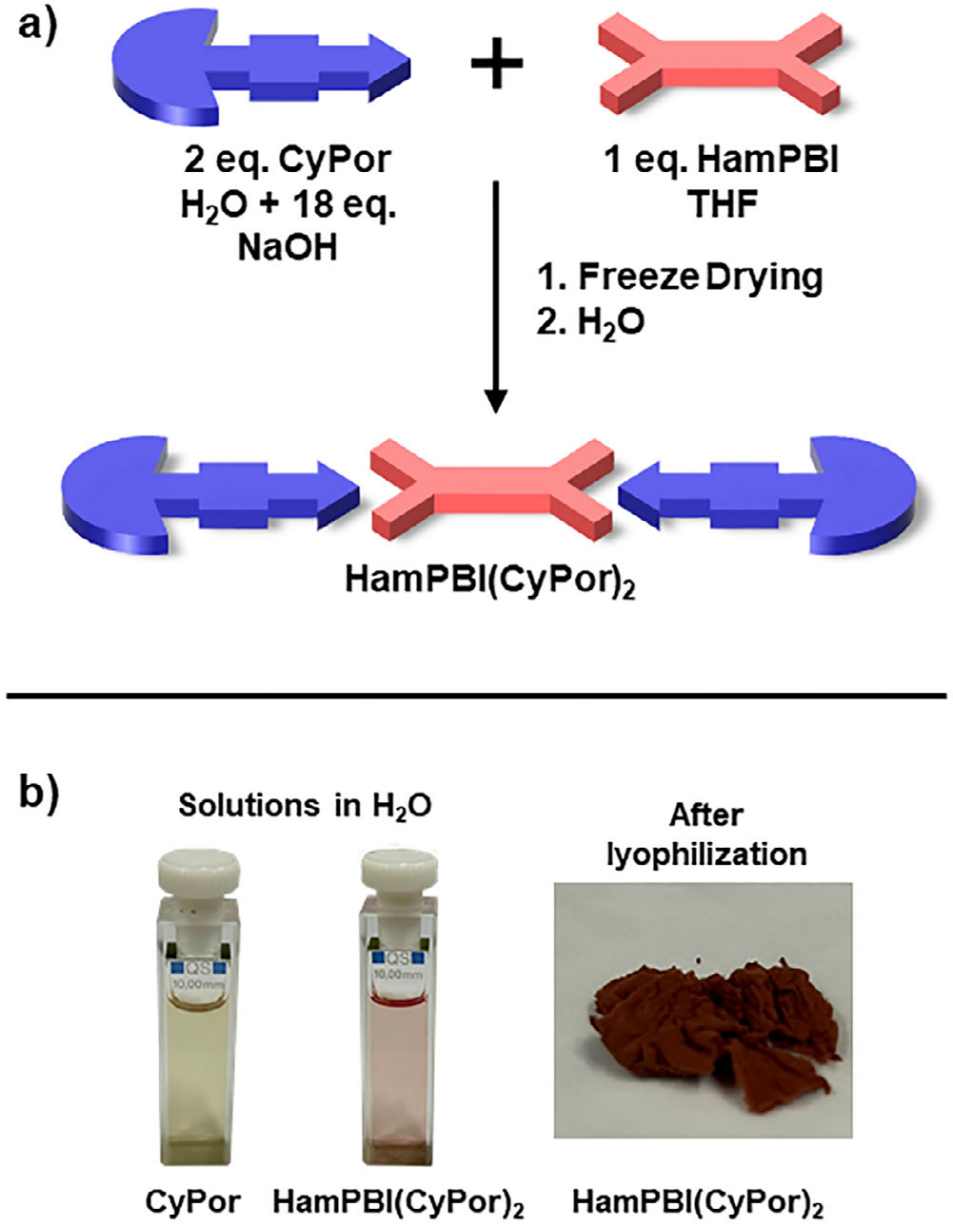
a) Illustration of the procedure to obtain **HamPBI(CyPor)_2_** supra‐amphiphile; b) images of **CyPor** and **HamPBI(CyPor)_2_** in aqueous solution (left) and of solid **HamPBI(CyPor)_2_** after lyophilization (right).

The spontaneous assembly of supra‐amphiphiles can generally be achieved after mixing the respective components in water.^[^ ^52^ ^]^ If one component is water insoluble, either a common solvent or a solvent mixture that promotes the assembly is usually utilized. The solvent is then removed and the newly formed amphiphiles are subsequently redispersed in, for example, aqueous solutions. Figure 2a illustrates our assembly procedure en‐route towards **HamPBI(CyPor)_2_**. As **CyPor** is insoluble in common organic solvents and **HamPBI** is insoluble in aqueous solutions, we opted for a mixture of basic aqueous solutions (1 eq. of NaOH per carboxylic acid) and THF in a ratio of 7.5:1 v/v, which readily dissolved both of them. This is, however, far from ideal, as the solvent mixture hinders the self‐assembly by forming H‐bonds. Nevertheless, upon removal of the solvent mixture via lyophilization, a spongy red solid was isolated. When resolving in pure water or basic buffers a clear solution is obtained. The red solutions remained stable for days due to the stabilization of **HamPBI** in the aqueous phase (Figure 2b).^[^ ^53^ ^]^

Characterization of **HamPBI(CyPor)_2_** by commonly employed methods like titrations or NMR spectroscopy provided significant challenges. In general, stabilization of **HamPBI** in aqueous solutions is thought to take either place by incorporation into the supra‐amphiphile or by a surfactant‐type process, where irregular aggregates of **HamPBI** are shielded by **CyPor**. Moreover, as demonstrated above, the supra‐amphiphile assembles into multiple conformations and allows for a diverse range of aggregation motifs. We started our investigations by means of ATR‐IR spectroscopy (Figures S8 and S9). The ATR‐IR spectra revealed the characteristic signals of the individual components, showing no significant alterations. A direct structural transfer from solid state to dispersion is highly unlikely, providing little reason to analyze the solid structure further. When turning to solution, we continued our investigations by performing ^1^H NMR. In D_2_O, only signals stemming from **CyPor** (Figure S56) were discernable. They originate from either residual dissolved species or from a shielding of the interieur of the aggregates. Titration assays with **CyPor** and **HamPBI** are unfeasible due to their incompatible solubility. Likewise, our efforts to employ DOSY NMR experiments were also unsuccessful.

**Figure 3:**
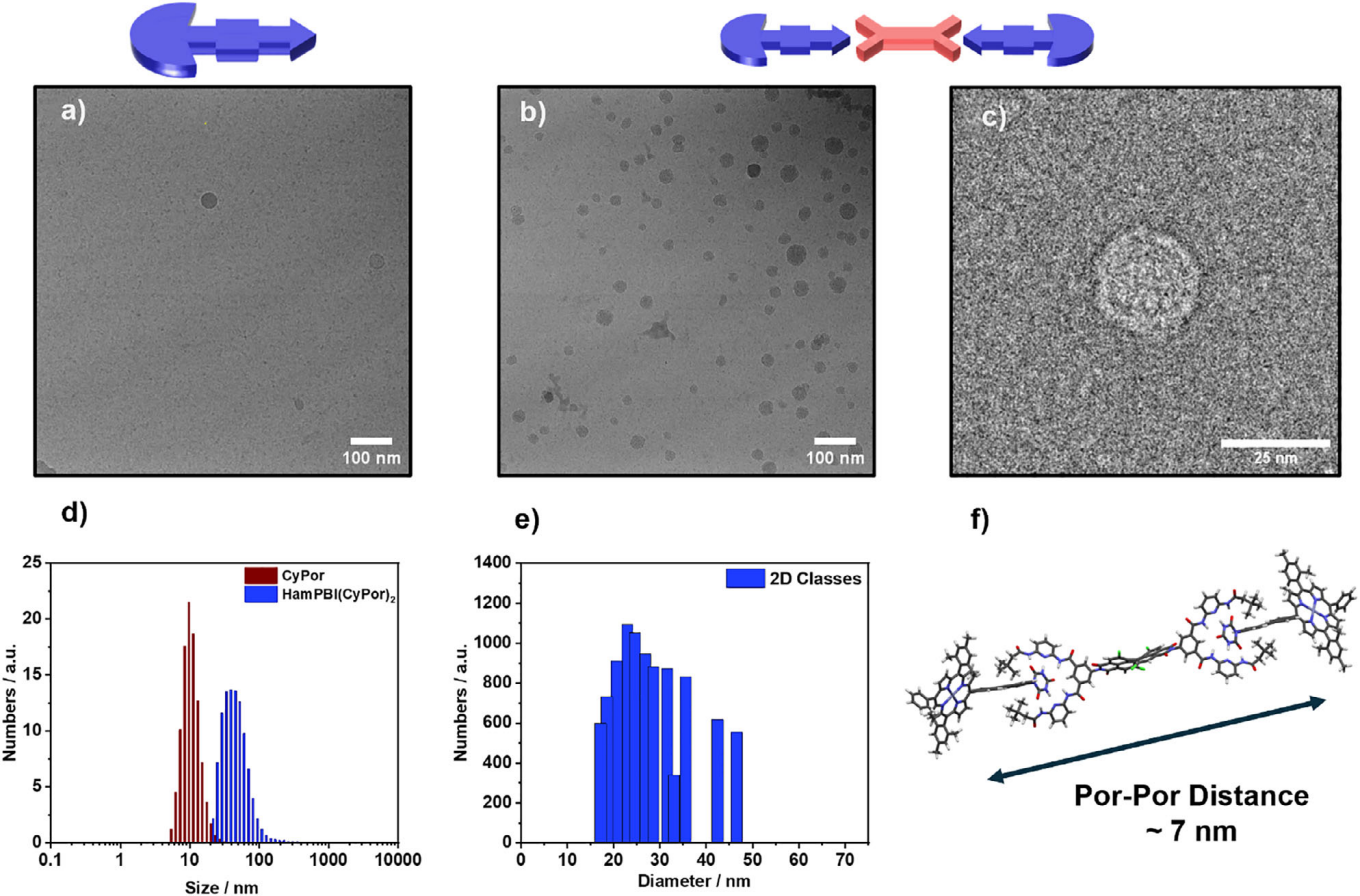
a) Cryo‐TEM image of a 2 mM aqueous solution of the sodium salt of **CyPor**, scalebar 100 nm; b) cryo‐TEM image of a 2 mM aqueous solution of **HamPBI(CyPor)_2_**, scalebar 100 nm; c) cryo‐TEM image of an extracted aggregate particle from a 2 mM aqueous solution of **HamPBI(CyPor)_2_**, scalebar 25 nm; d) DLS size distribution of **CyPor** (red) and **HamPBI(CyPor)_2_** (blue) in PO_4_‐buffered solutions (5 × 10^−6^ M; pH 7.2; average of three measurements); e) size distribution obtained from 2D classification of the automatically picked aggregates; f) calculated molecular structure of **HamPBI(CyPor)_2_** at the DFT level of theory (the dendrons were omitted for clarity).

First insights into the nature of the aggregates were obtained by dynamic light scattering (DLS) experiments. Here, the average hydrodynamic diameters obtained for **CyPor** and **HamPBI(CyPor)_2_** (Figure 3d) in a phosphate buffered solution (5 × 10^−6^ M) at pH 7.2 are 11.9 and 47.5 nm, respectively. In pure water the results are quite similar (Figure S10). The polydisperse size distribution for **HamPBI(CyPor)_2_** is broader than for **CyPor**. It reaches up to 100 nm and suggests the formation of large super‐structures.

Additional information, especially concerning the morphology, came from cryogenic electron transmission microscopy (cryo‐TEM). The sodium salt of **CyPor** and **HamPBI(CyPor)_2_** were both probed in aqueous 2 mM solutions. For **CyPor**, small particles in the range of a few nanometers were found. These are due to single solvated amphiphiles or small aggregates (Figure 3a). In stark contrast, cryo‐TEM images of **HamPBI(CyPor)_2_** show large, spherical assemblies in the range of approximately 15 to 50 nm (Figure 3b).^[^ ^54^ ^]^

We applied an automated picking and sorting procedure of single particles into averaged 2‐dimensional (2D) classes by size (Figure S12j) to gather data regarding the overall size distribution. This protocol has recently been established as a comprehensive tool in nanoparticle analysis.^[^ ^55^ ^]^ The resulting size distribution (Figure 3e) ranges from around 16 to 47 nm and maximizes at approximately 25 nm. A representative single particle is shown in Figure 3c. We also noticed particles, whose size is significantly outside from 16 to 47 nm range, and consequently cannot be picked automatically without altering the applied restrictions.^[^ ^56^ ^]^ These particles are ascribed to **HamPBI(CyPor)_2_** aggregates. Their smallest size is approximately twice that of what the DFT‐optimized structure for the S‐shape of **HamPBI(CyPor)_2_** predicts (Figure 3f). We failed, however, to resolve the molecular structure due to the polydisperse nature of the aggregates. In short, cryo‐TEM images underscore the successful formation of spherical particles with features that are distinctly different from those seen for **CyPor**.

### Steady‐State Absorption and Emission

**Figure 4:**
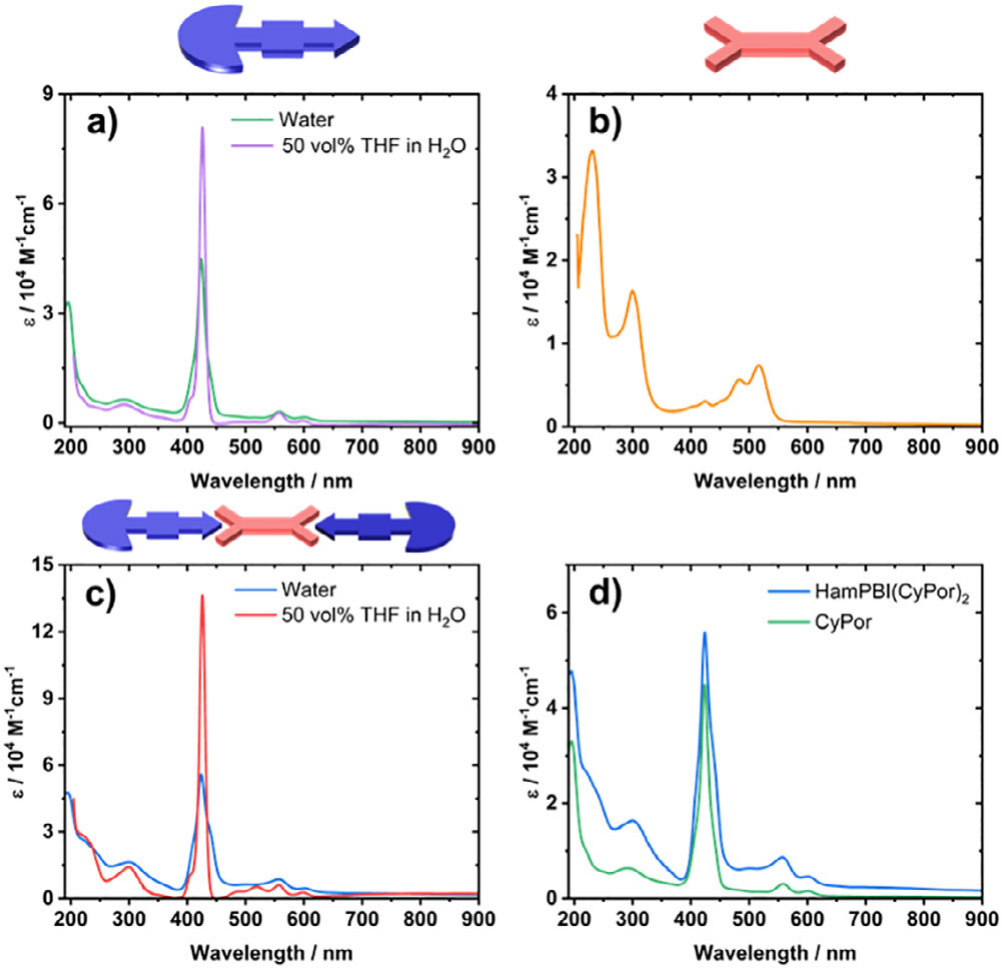
Steady‐state absorption spectra of a) **CyPor**, b) **HamPBI** in THF, c) **HamPBI(CyPor)_2_**, and d) **HamPBI(CyPor)_2_** in H_2_O and **CyPor** in H_2_O.

To probe the electronic interactions in the **HamPBI(CyPor)_2_** supra‐amphiphile, we first turned to absorption spectroscopy. **CyPor** in 1:1 v/v of THF and water shows transitions typically observed for porphyrins, namely intense Soret‐band absorptions and weaker Q‐band absorptions. The Soret‐band absorption / S_0_‐to‐S_2_ transition of **CyPor** is observed at 430 nm with an extinction coefficient of 8 × 10^5^ M^−1^cm^−1^, while the Q‐band absorption / S_0_‐to‐S_1_ transition appears at 550 nm with an extinction coefficient of 5000 M^−1^cm^−1^ (Figure 4a). **CyPor** shows in water the same characteristic transitions, but the Soret‐band absorption is broadened and weaker. This is indicative for mutual **CyPor** interactions in water. An increased background scattering also prompts to the formation of supramolecular assemblies. **HamPBI** absorptions in THF (Figure 4b) are best described by the characteristic absorption features for PBIs, namely the S_0_–S_2_ transitions at 300 nm and the S_0_–S_1_ transitions at 500 nm. **HamPBI(CyPor)_2_** shows spectral contributions from **HamPBI** and **CyPor** on top of background scattering (Figure 4c). The latter accounts for the presence of supra‐amphiphile assemblies in water. Once 50 vol% THF is added to an aqueous solution of **HamPBI(CyPor)_2_**, the absorptions become a clear superposition of **CyPor** and **HamPBI** in a 2:1 ratio and lack any background scattering (for stepwise addition of THF refer to Figure S13). This underlines the fact that the two components are separately solubilized without showing any mutual interactions. When comparing the absorptions of **CyPor** and **HamPBI(CyPor)_2_** in water (Figure 4d), an additional shoulder is observed at 441 nm, which is absent in **CyPor** and **HamPBI** and could be related to vibronic signatures, stemming from the **HamPBI** due to its absence in the **CyPor** aggregates.^[^ ^57^, ^58^ ^]^

**Figure 5:**
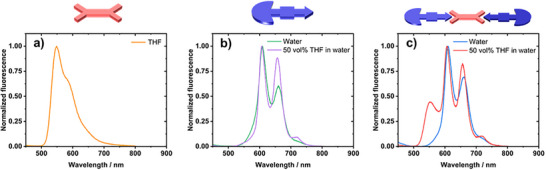
Normalized steady‐state fluorescence spectra of a) **HamPBI** in THF, b) **CyPor** in 50 vol% THF in H_2_O, and c) **HamPBI(CyPor)_2_** in 50 vol% in H_2_O with excitation at 300 nm.

When turning to steady‐state fluorescence spectroscopy, we gathered evidence for electronic interactions that dominate the excited‐state behavior. We employed 300 nm photoexcitation given that at this wavelength **HamPBI** as well as **CyPor** absorb. The fluorescence spectrum of **HamPBI** (Figure 5a) shows a maximum at 550 nm, which is ascribed to the *0–0 transition, while the shoulder at 580 nm corresponds to the *0–1 transition. In water, **CyPor** (Figure 5b) shows fluorescence maxima at 606 and 656 nm, which reflect the *0–0 and *0–1 transitions, respectively. Upon addition of 50 vol% THF, an additional fluorescence feature evolves at 718 nm. It is very likely that it originates from a vibrational level that is inaccessible in the assemblies. Also, the 656 nm feature is much stronger than in water. When turning to **HamPBI(CyPor)_2_**, the fluorescence spectrum in water resembles that noticed for **CyPor** in water (Figure 5c). In particular, maxima between 606 and 656 nm evolve albeit markedly quenched in comparison to **CyPor**. Please note that the **HamPBI**‐centered fluorescence is completely absent. At this point we conclude a near quantitative quenching of the **HamPBI**‐ and **CyPor**‐centered fluorescence and postulate either an energy or electron transfer in **HamPBI(CyPor)_2_** to be responsible. Interestingly, once 50 vol% THF is added to the aqueous dispersion, **HamPBI(CyPor)**_2_ fully dissolve and the fluorescence from **HamPBI** and **CyPor** is quantitatively recovered (Figure 5c).

To further asses the electronic interactions in **HamPBI(CyPor)_2_**, we performed electrochemical investigations of **CyPor** and **HamPBI(CyPor)_2_** in an aqueous 0.2 M KNO_3_ solution. Under oxidative conditions, **CyPor** and **HamPBI(CyPor)_2_** undergo oxidations at + 0.83 and + 0.8 V vs. Ag/AgCl, respectively. Both oxidations are irreversible (Figure S15). Under reductive conditions, **HamPBI(CyPor)_2_** failed to show the reductions that was noted for **HamPBI** in THF between ‐0.66 and ‐0.89 V vs. Fc/Fc^+^ (Figure S15). We rationalize the absence of **HamPBI** reduction in **HamPBI(CyPor)_2_** by its localization inside the hydrophobic pocket of the supra‐amphiphile.

### Transient Absorption Spectroscopy

To corroborate the nature of the excited‐state interactions in the **HamPBI(CyPor)_2_** supra‐amphiphile we performed femtosecond transient absorption spectroscopy (fsTA). To understand the deactivation processes in the **HamPBI(CyPor)_2_** supra‐amphiphile we studied its deactivation following photoexcitation at either 430 or 500 nm to selectively photoexcite **CyPor** or **HamPBI**, respectively.

fsTA raw data of **CyPor** following 430 nm photoexcitation in water are best described as ground state bleaching (GSB) at 560 and 600 nm next to excited state absorption (ESA) at 460, 660, and 870 nm and stimulated emission (SE) at 660 nm that evolves after a few picoseconds (Figure S16). fsTA raw data are fit with a kinetic model based on four sequentially formed species. The first species between 560 and 600 nm GSBs next to between 460 and 650 nm ESAs is attributed to an energetically higher lying second singlet excited state (S_2_) of **CyPor** (Figure S16). (S_2_) then decays with 1.8 ps into a hot singlet excited state (S_1_^hot^) with ESAs between 460 and 870 nm, GSBs between 560 and 600 nm, and SE at 660 nm. Next, (S_1_^hot^) transforms within 25.2 ps to a relaxed singlet excited state (S_1_^rel^), however, without showing any spectral changes. Finally, the fourth species is the triplet excited state (T_1_), which is characterized by the absence of the 650 nm SE and a 500 nm ESA. (T_1_) is populated from (S_1_^rel^) via intersystem crossing (ISC) that takes 3.3 ns. Lifetime determination of the long‐lived (T_1_) required nanosecond transient absorption (nsTA) (Figure S18). Here, the observed features include GSBs at 562 and 608 nm and an ESA at 466 nm. As such, they resemble the long‐lived state seen in the fsTA experiments. (T_1_) has a lifetime of 3.9 µs.^[^ ^59^ ^]^ fsTA of **HamPBI** in THF, when photoexcited at 500 nm are characterized by GSBs between 483 and 518 nm, SE at 550, and ESA at 980 nm (Figure S20). A sequential fit based on two species yielded two lifetimes with 19.7 and 790.3 ps (Figure S21). The short‐lived first species is assigned to a hot singlet excited state (S_1_^hot^), while the long‐lived second species relates to a relaxed singlet excited state (S_1_^rel^). **HamPBI** feature no discernable (T_1_).

**Figure 6:**
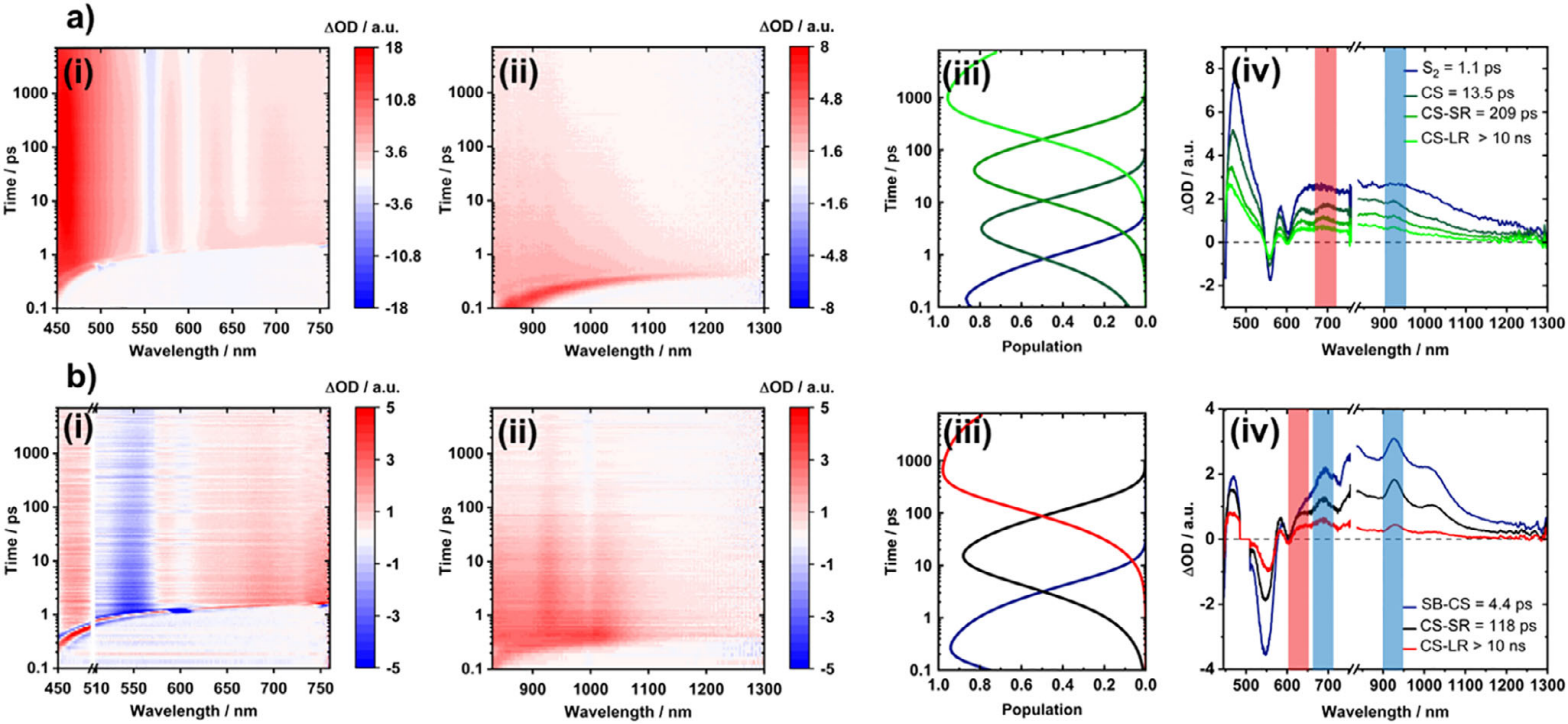
fsTA data of **HamPBI(CyPor)_2_** (1.7 × 10^−5^ M) in water a) photoexcited at 430 nm and b) photoexcited at 500 nm. All experiments were conducted using a 0.4 µJ pump energy. For each of the four examples the differential absorption spectra are shown in the (i) visible and (ii) near‐infrared region obtained in femtosecond transient absorption experiments, (iii) shows the population dynamics, and (iv) the deconvoluted spectra obtained through fitting the experimental data using a sequential model for (a) and (b). The obtained lifetimes are gathered in Table 1.

**Scheme 3:**
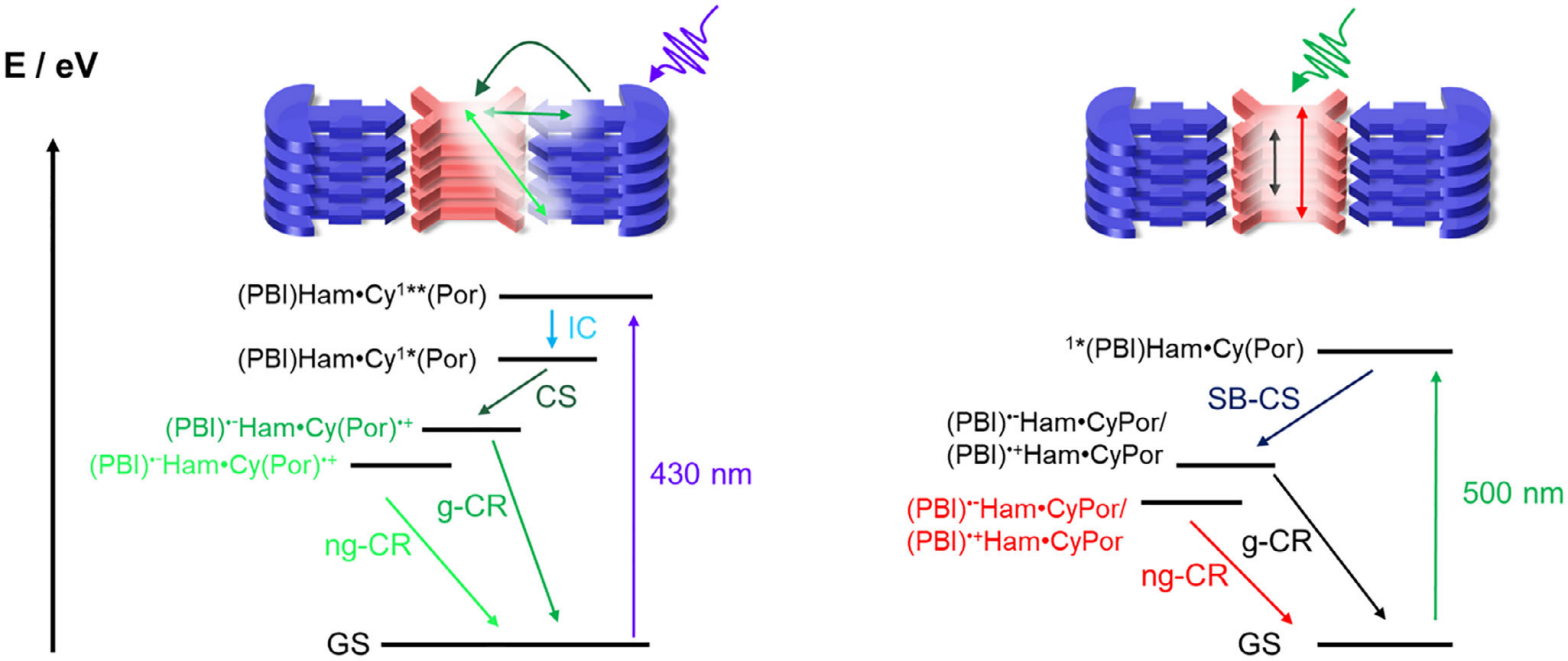
Proposed deactivation mechanism for the photoexcitation of **HamPBI(CyPor)_2_** at 430 (left) and 500 (right) nm; IC is internal conversion, CS is charge separation, g‐CR is geminate charge recombination, ng‐CR is nongeminate charge separation, SB‐CS is symmetry breaking charge separation.

When we employed 430 nm to photoexcite **HamPBI(CyPor)_2_** we observed GSBs between 558 and 602 nm and an ESA at 477 nm as well as a broad near‐infrared ESA within the first five picoseconds (Figure 6). Additionally, at longer times one observes the appearance of the **CyPor** SE signal evolving around 660 nm, as well as an ESA at 930 nm. The experimental data was deconvoluted using a sequential model based on four species (Figure 6). In line with the excitation wavelength, the first species with 477 nm ESA and 558 and 602 nm GSBs are assigned to (S_2_) of **CyPor** and it transforms within 1.1 ps into (S_1_^hot^) of **CyPor** as the second species. Rather than seeing the slow relaxation and ISC to afford (S_1_^rel^) and (T_1_), we note for the subsequently formed species, namely, the third one, the characteristic features of the one‐electron reduced form of **HamPBI** (Figure S22),^[^ ^60^ ^]^ that is, a 925 nm ESA (Figure 6a), and the one‐electron oxidized form of **CyPor**, that is, 645 and 690 nm ESAs (Figure S23). This prompts to a charge separation (CS) upon photoexcitation of **HamPBI(CyPor)_2_** to yield the corresponding charge separated state **{Ham(PBI)^•−^Cy(Por)^•+^}**. Based on the electrochemical measurements and the theoretical calculations – vide supra – a thermodynamically driven CS is feasible from (S_1_) of **CyPor** and from our fsTA experiments we conclude that CS takes 13.5 ps, which is of the same order than for a Hamilton system with a **Por** as electron donor and a fullerene as electronacceptor.^[^ ^45^ ^]^ Considering that the third and fourth species are spectroscopically identical we hypothesize that both relate to charge separated states **{Ham(PBI)^•−^Cy(Por)^•+^}**. Their lifetimes are 209 ps and >10 ns as they reflect geminate charge recombination (g‐CR) and non‐geminate charge recombination (ng‐CR), respectively (Scheme 3). Electron hopping between π–π stacked **Ham(PBI)**s enables hereby the transformation of an adjacent charge separated state into a distant one to drive g‐CR and ng‐CR, respectively. To corroborate not only the exact lifetime of ng‐CR, but also the product thereof, we turned to nsTA (Figure S24). To fit the corresponding raw data, we employed a kinetic model based on two consecutively formed species. The spectral characteristics of both of them match those seen for the charge separated state **{Ham(PBI)^•−^Cy(Por)^•+^}** on the fs‐TA timescale. We derive two lifetimes, that is, <1 ns and 1.6 µs, by which the ground state is recovered by means of g‐CR and ng‐CR, respectively. No long‐lived (T_1_) – neither **HamPBI**‐ nor **CyPor**‐centered – are noticed once g‐CR and ng‐CR come to an end.

When changing the wavelength of photoexciting **HamPBI(CyPor)_2_** to 500 nm the spectral features are characterized by a broad GSB at 550 nm as well as an ESA that ranges from 580 to 1300 nm (Figure 6). Of particular relevance is the near‐infrared region as it mirrors **Ham(PBI)**s‐centered states. To deconvolute the visible and near‐infrared regions of the fs‐TA required a three species kinetic model. In line with the excitation wavelength, the first species features a rather broad 550 nm GSB next to a 980 nm ESA and is assigned to (S_1_) of **Ham(PBI)** (Figure 6). Its lifetimes is short, as it transforms quickly within 2.0 ps into the second species with ESAs at 468, 590, 680, 930, and 1020 nms. Importantly, between 930 and 1020 nm ESAs are characteristic fingerprints known for the one‐electron reduced form of **Ham(PBI)**, while the 680 nm ESA stems from the one‐electron oxidized form of **Ham(PBI)**. In other words, **HamPBI(CyPor)_2_** undergoes upon 500 nm photoexcitation a symmetry breaking charge separation (SB‐CS) to form **{Ham(PBI)^•−^CyPor/Ham(PBI)^•+^CyPor}** rather than the charge separated state **{Ham(PBI)^•−^Cy(Por)^•+^}**. This process has previously been observed in PBI nanocrystals.^[^ ^61^ ^]^ Important is that SB‐CS outcompetes the much slower CS seen in the 430 nm photoexcitation experiments by one order of magnitude. A closer look at the processes following SB‐CS reveals that the second and third species are identical. Similar to CS seen upon 430 nm photoexcitation of **Cy(Por)**, SB‐CS upon 500 nm photoexcitation of **Ham(PBI)** creates first an adjacent symmetry broken charge separated state and second a distant one to drive g‐SB‐CR and ng‐SB‐CR, respectively. Both symmetry broken charge separated state **{Ham(PBI)^•−^CyPor/Ham(PBI)^•+^CyPor}** return to the ground state with 52 ps and >10 ns.^[^ ^62^ ^]^

Through combining the TA information, by means of photoexciting at either 430 or 500 nm, we confirm that CS takes place in the **HamPBI(CyPor)_2_** supra‐amphiphile. Depending on photoexciting **CyPor** or **HamPBI**, not only the mechanism of CS is different but also the outcome. It is either a very fast SB‐CS or a slower CS across the Hamilton acceptor‐donor system that occur within 2.0 or 13.5 ps, respectively.

Finally, to corroborate that SB‐CS and CS depend on the intact **HamPBI(CyPor)_2_** supra‐amphiphile, 50 vol% THF was added to the aqueous dispersion of **HamPBI(CyPor)_2_** and photoexcited at 430 nm (Figure S26) and 500 nm (Figure S28). In the first case, deconvolution of the fs‐TA data afforded four species with spectroscopic signatures and lifetimes that are similar to those observed for **CyPor** in 1:1 v/v of THF and water (Table 1). In the latter case, the same two species were found that evolved in the reference experiments with **HamPBI** in THF (Figures S27 and S29).

**Table 1:** Associated lifetimes obtained from fitting the fsTA data using the proposed deactivation scheme for **HamPBI(CyPor)_2_**.

	Excitation at 430 nm				Excitation at 500 nm		
	H_2_O		50 vol% THF in H_2_O		H_2_O	50 vol% THF in H_2_O	THF
	HamPBI (CyPor)_2_	CyPor	HamPBI (CyPor)_2_	CyPor	HamPBI (CyPor)_2_		HamPBI
**t_1_ / ps**	1.1	1.8	4.1	1.4	4.4	6.2	19.7
**t_2_ / ps**	13.5	25.2	61	77.9	118	344	790.3
**t_3_ / ps**	209	1100	1270	1920	>0.01	–	
**t_4_ / µs**	4.8	3.87	1.6	1.8	–	–	

## Conclusions

In summary, we report the synthesis and assembly of a bola‐type porphyrin‐PBI supra amphiphile **HamPBI(CyPor)_2_** consisting of an amphiphilic cyanuric acid bearing porphyrin (**CyPor**) and a *bis*‐Hamilton receptor PBI (**HamPBI**). The assembly takes place through an unprecedented stabilization principle, which is based on a conventional amphiphilic **CyPor** and hydrophobic **HamPBI**, forming the resulting bola‐form solely through the H‐bonding motif, which is shielded by the assembly of the chromophores itself. The amphiphilic porphyrin, in which an oligo carboxylic acid Newkome dendron is used as the polar head group is well soluble in basic aqueous solutions, while PBI is highly insoluble in water. Assembly of the supra‐amphiphile was achieved by mixing of the single components with 1 eq. NaOH per carboxylic acid in THF/water mixtures, followed by lyophilization and subsequent redispersion in pure water. Notably, the resulting dispersions are stable over days, indicating successful stabilization of the PBI by the amphiphilic porphyrin. Complementary analysis of the morphology and size of the **HamPBI(CyPor)_2_** supra‐amphiphile via cryo‐TEM imaging and DLS reveal spherical aggregates in the size range of approximately 15 – 100 nm, which cannot be observed for the single amphiphilic porphyrin. The polydisperse size distribution of the aggregates precluded us from performing further structural analysis.

Investigating the photophysical properties of the systems further demonstrates its successful stabilization. For example, absorption spectra of **HamPBI(CyPor)_2_** in pure water show broadened features of both porphyrin and PBI, as well as strongly quenched emission of the **HamPBI** in fluorescence spectroscopy. Addition of THF leads to a denaturation of the supra‐amphiphile, which reinstates both absorption and fluorescence characteristics of separately solubilized chromophores. Femtosecond transient absorption spectroscopy (fsTAS) corroborated in pure water the formation of a charge separated state following photoexcitation and charge separation (CS) or symmetry breaking charge separation (SB–CS) upon either **CyPor** 430 nm photoexcitation or **HamPBI** 500 nm photoexcitation, respectively. None of them, that is, neither CS nor SB‐CS, occurred in THF/water mixtures.

These results represent a proof‐of‐concept for a powerful strategy to simply construct highly complex functional supra‐amphiphiles. Moreover, our new self‐assembled architecture represents the first example for the realization of targeted supramolecular organization of multiple photoactive chromophores in water, resembling core‐principles of artificial photosynthesis. Based on this, future endeavors will include the precise structural analysis on the molecular level and derivatization of the building blocks by using different functional chromophores. These investigations will also give more insights into the presence of certain underlying criteria, which allow for successful stabilization of the hydrophobic chromophores within these aggregates.

## Supporting Information

The authors have cited additional references within the Supporting Information.^[^ ^63^, ^64^, ^65^, ^66^, ^67^, ^68^, ^69^, ^70^, ^71^, ^72^, ^73^, ^74^, ^75^, ^76^ ^]^

## Conflict of Interests

The authors declare no conflict of interest.

## Supporting information



Supporting Information

## Data Availability

The data that support the findings of this study are available from the corresponding author upon reasonable request.

## References

[1] M. R. Wasielewski , Chem. Rev. 1992, 92, 435–461, 10.1021/cr00011a005.

[2] D. Gust , T. A. Moore , A. L. Moore , Acc. Chem. Res. 2009, 42, 1890–1898, 10.1021/ar900209b. 19902921

[3] G. Bottari , O. Trukhina , M. Ince , T. Torres , Coord. Chem. Rev. 2012, 256, 2453–2477, 10.1016/j.ccr.2012.03.011.

[4] Y. Li , T. Liu , H. Liu , M. Z. Tian , Y. Li , Acc. Chem. Res. 2014, 47, 1186–1198, 10.1021/ar400264e. 24666347

[5] M. R. Wasielewski , Acc. Chem. Res. 2009, 42, 1910–1921, 10.1021/ar9001735. 19803479

[6] M. D. Ward , Chem. Soc. Rev. 1997, 26, 365, 10.1039/cs9972600365.

[7] F. Zhao , C. Wang , X. Zhan , Adv. Energy Mater. 2018, 8, 1703147, 10.1002/aenm.201703147.

[8] J. Hou , O. Inganäs , R. H. Friend , F. Gao , Nat. Mater. 2018, 17, 119–128, 10.1038/nmat5063. 29358765

[9] J. Yu , L. Huang , Q. Tang , S.‐B. Yu , Q.‐Y. Qi , J. Zhang , D. Ma , Y. Lei , J. Su , Y. Song , J.‐C. Eloi , R. L. Harniman , U. Borucu , L. Zhang , M. Zhu , F. Tian , L. Du , D. L. Phillips , I. Manners , R. Ye , J. Tian , Nat. Catal. 2023, 6, 464–475, 10.1038/s41929-023-00962-z.

[10] R. Charvet , Y. Yamamoto , T. Sasaki , J. Kim , K. Kato , M. Takata , A. Saeki , S. Seki , T. Aida , J. Am. Chem. Soc. 2012, 134, 2524–2527, 10.1021/ja211334k. 22280067

[11] B. Matarranz , G. Fernández , Chem. Phys. Rev. 2021, 2, 041304, 10.1063/5.0065873.

[12] R. M. Veedu , Z. Fernández , N. Bäumer , A. Albers , G. Fernández , Chem. Sci. 2024, 15, 10745–10752, 10.1039/D4SC02499K. 39027305 PMC11253169

[13] N. Bäumer , S. Ogi , L. Borsdorf , S. Yamaguchi , G. Fernández , Chem. Commun. 2023, 59, 8937–8940, 10.1039/D3CC02297H. 37365975

[14] Y. Yamamoto , T. Fukushima , Y. Suna , N. Ishii , A. Saeki , S. Seki , S. Tagawa , M. Taniguchi , T. Kawai , T. Aida , Science 2006, 314, 1761–1764, 10.1126/science.1134441. 17170300

[15] W. Zhang , W. Jin , T. Fukushima , N. Ishii , T. Aida , J. Am. Chem. Soc. 2013, 135, 114–117, 10.1021/ja311738m. 23252447

[16] M. Sun , K. Müllen , M. Yin , Chem. Soc. Rev. 2016, 45, 1513–1528, 10.1039/C5CS00754B. 26797049

[17] C. D. Schmidt , C. Böttcher , A. Hirsch , European J. Org. Chem. 2007, 2007, 5497–5505, 10.1002/ejoc.200700567.

[18] H. Wu , L. Xue , Y. Shi , Y. Chen , X. Li , Langmuir 2011, 27, 3074–3082, 10.1021/la104888p. 21319851

[19] X. Wang , T. Zeng , M. Nourrein , B. H. Lai , K. Shen , C. L. Wang , B. Sun , M. Zhu , RSC Adv. 2017, 7, 26074–26081, 10.1039/c7ra04296e

[20] A. Hirsch , Pure Appl. Chem. 2008, 80, 571–587, 10.1351/pac200880030571.

[21] S. Burghardt , A. Hirsch , B. Schade , K. Ludwig , C. Böttcher , Angew. Chemie – Int. Ed. 2005, 44, 2976–2979, 10.1002/anie.200462465. 15830405

[22] M. Brettreich , S. Burghardt , C. Böttcher , T. Bayerl , S. Bayerl , A. Hirsch , Angew. Chem. Int. Ed. 2000, 39, 1845–1848, 10.1002/(SICI)1521-3773(20000515)39:10<1845::AID-ANIE1845>3.0.CO;2-Q. 10934382

[23] S. Tu , S. H. Kim , J. Joseph , D. A. Modarelli , J. R. Parquette , ChemPhysChem 2013, 14, 1609–1617, 10.1002/cphc.201300023 23564748

[24] S. Tu , S. H. Kim , J. Joseph , D. A. Modarelli , J. R. Parquette , J. Am. Chem. Soc. 2011, 133, 19125–19130, 10.1021/ja205868b. 22004360

[25] R. S. Loewe , K. ya Tomizaki , F. Chevalier , J. S. Lindsey , J. Porphyr. Phthalocyanines 2002, 06, 626–642, 10.1142/S1088424602000774.

[26] G. Hu , H. S. Kang , A. K. Mandal , A. Roy , C. Kirmaier , D. F. Bocian , D. Holten , J. S. Lindsey , RSC Adv. 2018, 8, 23854–23874, 10.1039/c8ra04052d. 35540249 PMC9081848

[27] R. S. Loewe , K. ya Tomizaki , W. J. Youngblood , Z. Bo , J. S. Lindsey , J. Mater. Chem. 2002, 12, 3438–3451, 10.1039/B205680A.

[28] E. J. Schulze , C. L. Ritterhoff , E. Franz , O. Tavlui , O. Brummel , B. Meyer , A. Hirsch , Chem. – A Eur. J. 2024, 30, e202303515, 10.1002/chem.202303515. 38200652

[29] E. J. Schulze , M. Wu , C. D. Methfessel , E. Spiecker , A. Hirsch , Chem. – A Eur. J. 2025, 31, e202500279, 10.1002/chem.202500279. PMC12133624 40252076

[30] Y. Kang , K. Liu , X. Zhang , Langmuir 2014, 30, 5989–6001, 10.1021/la500327s. 24617560

[31] B. Liu , W. Zhu , Y. Wang , W. Wu , X. Li , B. Chen , Y. T. Long , Y. Xie , J. Mater. Chem. 2012, 22, 7434, 10.1039/c2jm16804a.

[32] Y. Kang , X. Tang , Z. Cai , X. Zhang , Adv. Funct. Mater. 2016, 26, 8920–8931, 10.1002/adfm.201602998.

[33] G. Ouyang , M. Liu , Mater. Chem. Front. 2020, 4, 155–167, 10.1039/C9QM00571D.

[34] H.‐W. Tian , Y.‐C. Liu , D.‐S. Guo , Mater. Chem. Front. 2020, 4, 46–98, 10.1039/C9QM00489K.

[35] H. Zhu , L. Shangguan , B. Shi , G. Yu , F. Huang , Mater. Chem. Front. 2018, 2, 2152–2174, 10.1039/C8QM00314A.

[36] Z. Q. Li , Y. M. Zhang , Y. Chen , Y. Liu , Chem. – A Eur. J. 2014, 20, 8566–8570, 10.1002/chem.201402612. 24890802

[37] X. Chi , H. Zhang , G. I. Vargas‐Zúñiga , G. M. Peters , J. L. Sessler , J. Am. Chem. Soc. 2016, 138, 5829–5832, 10.1021/jacs.6b03159. 27123813

[38] S. P. Wang , W. Lin , X. Wang , T. Y. Cen , H. Xie , J. Huang , B. Y. Zhu , Z. Zhang , A. Song , J. Hao , J. Wu , S. Li , Nat. Commun. 2019, 10, 1399, 10.1038/s41467-019-09363-y. 30923311 PMC6438973

[39] C. Wang , S. Yin , S. Chen , H. Xu , Z. Wang , X. Zhang , C. Wang , S. C. Yin , S. L. Chen , H. P. Xu , Z. Q. Wang , X. Zhang , Angew. Chem. Int. Ed. 2008, 47, 9049–9052, 10.1002/anie.200803361. 18937241

[40] N. Kimizuka , T. Kawasaki , K. Hirata , T. Kunitake , J. Am. Chem. Soc. 1998, 120, 4094–4104, 10.1021/ja974379+.

[41] N. Kimizuka , T. Kawasaki , T. Kunitake , J. Am. Chem. Soc. 1993, 115, 4387–4388, 10.1021/ja00063a077.

[42] S. A. Wagay , R. Ali , Top. Curr. Chem. 2024, 382, 27, 10.1007/s41061-024-00471-y. 39033235

[43] J. F. Gnichwitz , M. Wielopolski , K. Hartnagel , U. Hartnagel , D. M. Guldi , A. Hirsch , J. Am. Chem. Soc. 2008, 130, 8491–8501, 10.1021/ja8018065. 18540584

[44] M. Wachter , B. Scholz , E. J. Schulze , F. Hampel , M. E. Pérez‐Ojeda , A. Hirsch , Chem. – A Eur. J. 2024, 30, e202400915, 10.1002/chem.202400915. 38616170

[45] F. Wessendorf , B. Grimm , D. M. Guldi , A. Hirsch , J. Am. Chem. Soc. 2010, 132, 10786–10795, 10.1021/ja101937w. 20681711

[46] K. Maurer , B. Grimm , F. Wessendorf , K. Hartnagel , D. M. Guldi , A. Hirsch , European J. Org. Chem. 2010, 2010, 5010–5029, 10.1002/ejoc.201000233.

[47] M. Ali , E. Kataev , J. Müller , H. Park , M. Halik , A. Hirsch , Chem. – A Eur. J. 2021, 27, 16429–16439, 10.1002/chem.202102581. PMC9297977 34651355

[48] T. Luchs , A. Zieleniewska , A. Kunzmann , P. R. Schol , D. M. Guldi , A. Hirsch , Chem. – A Eur. J. 2021, 27, 5041–5050, 10.1002/chem.202004928. PMC7986074 33428285

[49] L. Bao , B. Zhao , M. Ali , M. Assebban , B. Yang , M. Kohring , D. Ryndyk , T. Heine , H. B. Weber , M. Halik , F. Hauke , A. Hirsch , Adv. Mater. Interfaces 2022, 9, 2200425, 10.1002/admi.202200425.

[50] J. M. McGrath , M. D. Pluth , J. Org. Chem. 2014, 79, 711–719, 10.1021/jo402500a. 24377967 PMC4002384

[51] C. Dethlefs , J. Eckelmann , H. Kobarg , T. Weyrich , S. Brammer , C. Näther , U. Lüning , European J. Org. Chem. 2011, 2011, 2066–2074, 10.1002/ejoc.201001684.

[52] X. Zhang , C. Wang , Chem. Soc. Rev. 2011, 40, 94–101, 10.1039/B919678C. 20890490

[53] For clarity, we refer to the here formed substance as **HamPBI(CyPor)_2_ **, despite the possibility of other motifs being present in solution.

[54] We want to note, that also larger agglomerates and crystalline segments are observable (Figure S11). Their presence is attributed to incompletely dissolved residues that result from using high concentrations of material.

[55] I. Harley , A. Kaltbeitzel , F. Mazzotta , K. Koynov , S. S. Lembke , T. P. Doan‐Nguyen , K. Landfester , I. Lieberwirth , Nanoscale Horiz. 2025, 10, 1642–1652, 10.1039/D5NH00094G. 40452314

[56] It is important to note that due to the finite thickness of ice, the diameter distribution of very large particles is likely affected. Therefore, we decided not to include them in the given size distribution.

[57] N. J. Hestand , F. C. Spano , Chem. Rev. 2018, 118, 7069–7163, 10.1021/acs.chemrev.7b00581. 29664617

[58] We also performed titration studies by adding a concentrated **HamPBI** (2 × 10^‐4^ M) THF solution to a dilute **CyPor** solution (2 × 10^‐6^ M) in, water. Notable is the fact that the resulting absorption (Figure S14) differs from that seen for **HamPBI(CyPor)_2_ ** in water (Figure 4c). Furthermore, we performed temperature and time dependend measurement of these solutions, which did not yield spectra resembling **HamPBI(CyPor)_2_ ** as well (Figure S14). This could be reasoned in the competitive self‐assembly of **CyPor** and in the inability of **HamPBI**, which is not soluble in water, to access the site where H‐bond formation would be expected between the Hamilton receptor and the cyanuric acid. Furthermore, THF can effectively form competitive hydrogen bonds, hindering the spontaneous assembly of the aggregates when mixing solutions of **HamPBI** in THF and **CyPor** in water.

[59] fsTA of **CyPor** when 50 vol% of THF are added and photoexcited at 430 nm (Figure S17) are superimposable to those seen in water. Notable is, however, that the 660 nm SE is much stronger in the presence of THF. This supports the notion that THF denaturates the **CyPor** assemblies. The ESA at 870 nm is not observed once THF is added. This feature probably stems from some aggregation induced process. Nonetheless, the four lifetimes and the associated spectra though fitting the raw data using a sequential model (Figure S19) only reveal subtle changes, which are explained by a faster vibrational relaxation in the aggregates (Table 1).

[60] P. E. Hartnett , C. M. Mauck , M. A. Harris , R. M. Young , Y. L. Wu , T. J. Marks , M. R. Wasielewski , J. Am. Chem. Soc. 2017, 139, 749–756, 10.1021/jacs.6b10140. 28026177

[61] C. Schierl , A. Niazov‐Elkan , L. J. W. Shimon , Y. Feldman , B. Rybtchinski , D. M. Guldi , Nanoscale 2018, 10, 20147–20154, 10.1039/C8NR04155E. 30221262

[62] ns‐TA experiments to resolve the longer‐lived SB‐CS were unsuccessful.

[63] M. J. Plater , J. P. Sinclair , S. Aiken , T. Gelbrich , M. B. Hursthouse , Tetrahedron 2004, 60, 6385–6394, 10.1016/j.tet.2004.03.059.

[64] P. Giannozzi , S. Baroni , N. Bonini , M. Calandra , R. Car , C. Cavazzoni , D. Ceresoli , G. L. Chiarotti , M. Cococcioni , I. Dabo , A. Dal Corso , S. de Gironcoli , S. Fabris , G. Fratesi , R. Gebauer , U. Gerstmann , C. Gougoussis , A. Kokalj , M. Lazzeri , L. Martin‐Samos , N. Marzari , F. Mauri , R. Mazzarello , S. Paolini , A. Pasquarello , L. Paulatto , C. Sbraccia , S. Scandolo , G. Sclauzero , A. P. Seitsonen , et al., J. Phys. Condens. Matter 2009, 21, 395502, 10.1088/0953-8984/21/39/395502. 21832390

[65] J. P. Perdew , K. Burke , M. Ernzerhof , Phys. Rev. Lett. 1996, 77, 3865–3868, 10.1103/PhysRevLett.77.3865. 10062328

[66] S. Grimme , J. Antony , S. Ehrlich , H. Krieg , J. Chem. Phys. 2010, 132, 10.1063/1.3382344. 20423165

[67] S. Grimme , S. Ehrlich , L. Goerigk , J. Comput. Chem. 2011, 32, 1456–1465, 10.1002/jcc.21759. 21370243

[68] D. Vanderbilt , Phys. Rev. B 1990, 41, 7892–7895, 10.1103/PhysRevB.41.7892. 9993096

[69] F. Neese , Wiley Interdiscip. Rev. Comput. Mol. Sci. 2012, 2, 73–78, 10.1002/wcms.81.

[70] C. Lee , W. Yang , R. G. Parr , Phys. Rev. B 1988, 37, 785–789, 10.1103/PhysRevB.37.785. 9944570

[71] A. D. Becke , J. Chem. Phys. 1993, 98, 1372–1377, 10.1063/1.464304.

[72] F. Weigend , R. Ahlrichs , Phys. Chem. Chem. Phys. 2005, 7, 3297, 10.1039/b508541a. 16240044

[73] F. Neese , F. Wennmohs , A. Hansen , U. Becker , Chem. Phys. 2009, 356, 98–109, 10.1016/j.chemphys.2008.10.036.

[74] T. Yanai , D. P. Tew , N. C. Handy , Chem. Phys. Lett. 2004, 393, 51–57, 10.1016/j.cplett.2004.06.011.

[75] J. J. Snellenburg , S. P. Laptenok , R. Seger , K. M. Mullen , I. H. M. van Stokkum , J. Stat. Softw. 2012, 49, 1–22, 10.18637/jss.v049.i03.

[76] I. H. M. van Stokkum , D. S. Larsen , R. van Grondelle , Biochim. Biophys. Acta – Bioenerg. 2004, 1657, 82–104, 10.1016/j.bbabio.2004.04.011. 15238266

