# Supplementary material for: Multidirectional Charge Separation in Self‐Assembled Aggregates of Perylenebisimide‐Porphyrin Bola‐Supra‐Amphiphiles
Source: Angew Chem Int Ed Engl. 2026 Jan 5;65(10):e23324. doi: 10.1002/anie.202523324 (PMC12955521; doi:10.1002/anie.202523324)
Supplement: Supplementary file 1 — Supporting Information [file ANIE-65-e23324-s001.pdf]

Multidirectional Charge Separation in Self-Assembled Aggregates of Perylenebisimide-Porphyrin Bola-Supra-Amphiphiles

Erik J. Schulze,[a]† Elena A. Mack,[b]† Christian L. Ritterhoff,[c] Ufuk Borucu,[d]
Bernd Meyer,[c] Dirk M. Guldi,[b]* Andreas Hirsch[a]*

[a] E.J. Schulze, Prof. Dr. A. Hirsch
Department of Chemistry and Pharmacy, Chair of Organic Chemistry II
Friedrich-Alexander-Universität Erlangen-Nürnberg
Nikolaus-Fiebiger-Straße 10, 91058 Erlangen, Germany.
E-mail: andreas.hirsch@fau.de

[b] E.A. Mack, Prof. Dr. D.M. Guldi
Department of Chemistry and Pharmacy, Chair of Physical Chemistry I
Friedrich-Alexander-Universität Erlangen-Nürnberg
Egerlandstraße 3, 91058 Erlangen, Germany.
E-mail: dirk.guldi@fau.de

[c] C.L. Ritterhoff, Prof. Dr. B. Meyer
Interdisciplinary Center for Molecular Materials (ICMM) and Computer Chemistry Center (CCC)
Friedrich-Alexander-Universität Erlangen-Nürnberg
Nägelsbachstraße 25, 91052 Erlangen, Germany.

[d] Dr. U. Borucu
GW4 Facility for High-Resolution Electron Cryo-Microscopy
University of Bristol
24 Tyndall Avenue, Bristol, United Kingdom

[†] These authors contributed equally to this work.

Table of Contents

Materials and Methods 3
Synthetic Procedures 5
Theoretical Investigations 13
ITC Measurement 23
IR Spectroscopy 25
DLS Measurement 27
Cryo-TEM Imaging 28
Photophysical Investigations 29
NMR Spectroscopy 36
Mass Spectrometry 55

Materials and Methods

Synthesis and characterization

Reagents and solvents were obtained from commercial suppliers and used without any further purification. Solvents of technical grade were distilled prior to usage. HPLC solvents were used as received. Thin layer chromatography (TLC) was performed on Merck silica gel 60 F524, detected by UV-light (254 nm, 366 nm). Plug chromatography and column chromatography were performed on Macherey-Nagel silica gel 60 M (deactivated, 230−400 mesh, 0.04−0.063 mm). Gel permeation size exclusion chromatography was performed on BioBeads SX1 from BioRad in $CHCl_3$. NMR spectra were recorded on a Bruker Avance 400 (^{1}H: 400 MHz, ^{13}C: 101 MHz), a Bruker Avance 500 (^{1}H: 500 MHz, ^{13}C: 126 MHz), or a Bruker Avance Neo Cryo-Probe DCH (^{1}H: 600 MHz, ^{13}C: 150 MHz). Deuterated solvents were purchased from Sigma-Aldrich, ARMAR Isotopes and Deutero and used as received. Chemical shifts are given in ppm at room temperature and are referenced to residual protic impurities in the solvents (^{1}H: $CDCl_3$: 7.26 ppm, CD_2Cl_2: 5.34 ppm, THF-d_8: 3.58 ppm, D_2O: 4.79 ppm) or the deuterated solvent itself (^{13}C{^{1}H}: $CDCl_3$: 77.16 ppm, CD_2Cl_2: 53.4 ppm, THF-d_8: 66.57 ppm). The resonance multiplicities are indicated as “s” (singlet), “brs” (broad singlet), “d” (doublet), “t” (triplet), “q” (quartet) and “m” (multiplet). Mass spectrometry was carried out with a Shimadzu AXIMA Confidence (MALDI-TOF, matrix: 2,5-dihydroxybenzoic acid DHB, trans-2-[3-(4-tert-butylphenyl)-2-methyl-2-propenyliden]-malononitrile, (DCTB) or without matrix (OM). High resolution mass spectrometry (HRMS) was recorded on a LDI/MALDI-ToF Bruker Ultraflex Extreme machine or on a APPI-ToF mass spectrometer Bruker maXis 4G UHR MS/MS spectrometer. UV/vis spectroscopy was carried out on a Varian Cary 5000 UV−vis−NIR spectrometer. The spectra were recorded at rt in degassed THF / 0.1 M NaOH solution in quartz cuvettes (edge length = 1 cm) under ambient conditions. Fluorescence spectra were obtained from a Shimadzu RF-5301 PC and a NanoLog spectrofluorometer and a Fluoro Max 4 spectrofluorometer (Horiba Scientific). Dynamic Light Scattering (DLS) was performed on a Zetasizer Nano Series ZEN3600 (Malvern Instruments) with a 633 nm He-Ne laser. IR was measured on an PerkinElmer Frontier FT-IR spectrometer equipped with a PerkinElmer “Universal ATR Sampling Accessory” unit.

Steady-state spectroscopy

Steady-state absorption was performed using a Shimadzu UV-1900i UV-Vis spectrophotometer double beam spectrometer (190 to 1100 nm). Steady-state emission/excitation spectroscopy was recorded on an FS5 spectrofluorometer from Edinburgh Instruments using 10 mm optical path cuvettes.

Electrochemical characterizations

Cyclic and square wave voltammograms were measured with a Metrohm FRA 2 µAutolab Type III potentiostat. The phthalocyanine solutions in anhydrous DMF (Sigma Aldrich, CAS 68-12-2) were millimolar in the compound and 0.2 M in tetrabutylammonium hexafluorophosphate ($TBAPF_6$). The three-electrode arrangement included a glassy carbon (9.4 mm^2 area) working electrode, a counter electrode made of a Pt wire, and an Ag wire acting as a pseudo-reference electrode. Ferrocene was used as an internal reference.

Spectroelectrochemistry

Spectroelectrochemistry was measured in an OTTLE (Optically Transparent Thin-Layer Electrochemical) cell using a Pt mesh as a working electrode, a Pt electrode as a counter electrode, and an Ag electrode as a pseudo-reference electrode. The phthalocyanine solutions in anhydrous DMF were micromolar in the compound and 0.2 M in $TBAPF_6$. Spectra were obtained with an Avantes AvaLight/AvaSpec fiberspectrophotometer in the visible and near-infrared region.

Femtosecond transient absorption

The fsTA was conducted using an Astrella-F-1K amplified Ti:sapphire femtosecond laser system from Coherent, operating at a repetition rate 1kHz, 5.5 W power (5 mJ pulse energy), with a pulse duration of 80 fs. TA pump/probe Helios (fsTA) and EOS (nsTA) detection systems from Ultrafast Systems were utilized. In the fsTA experiment, an optical delay line placed in the probe beam pathway allowed for time delays up to 7.2 ns. White light was generated by focusing a fraction of the fundamental 800 nm output onto 2 mm (vis) or 2 cm (near-infrared) sapphire crystals. A 1.2 mJ fraction of the fundamental is used for pump beam generation by a TOPAS Prime from Light Conversion with standard extension. Pump energy was typically 1000 nJ. A depolarizer was placed in the pump beam to avoid rotational dynamics. Bandpass filters with ± 5 or ± 10 nm were used to ensure low spectral width and to exclude 800 nm photons. Typical excitation spot diameters are around 500 µm and ensured to be larger than those of the probe beam. The sample solutions were measured in a 2 mm quartz cuvette with absorbances of 0.2-0.5 at room temperature. To analyze transient absorption data, we used a suggested procedure.[1] We start with SVD and global analysis, using an all-sequential decay model that provides evolution associated spectra of potentially intervening species, to determine the number of decaying species that participate in the decay cascade. Obtained data were treated by SVD, global and target analyses using the R- package TIMP and GloTarAn.[1],[2] The instrument response function (IRF) and dispersion (chirp of the white light pulse) were modelled and taken into account during the fitting procedure.

Synthetic Procedures

For the synthetic procedures for **G2-NH$_2$**, **NH$_2$-Hamilton** and **ZnPor(COOMe)** were refer to the literature.[3],[4]

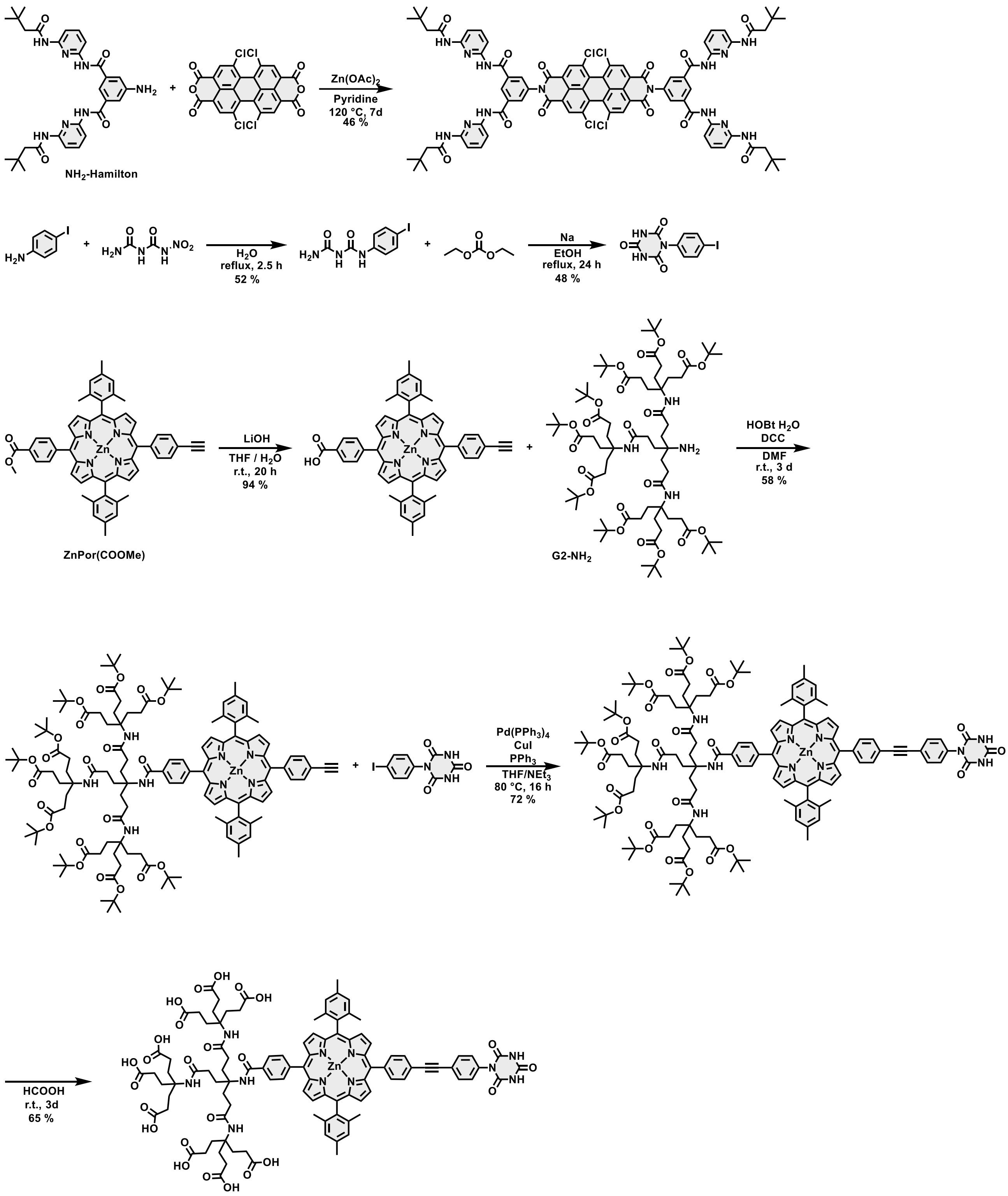

Scheme S1 Overview over the synthetic pathway towards **CyPor** and **HamPBI**.

Bis Hamilton perylenebisimide (HamPBI)

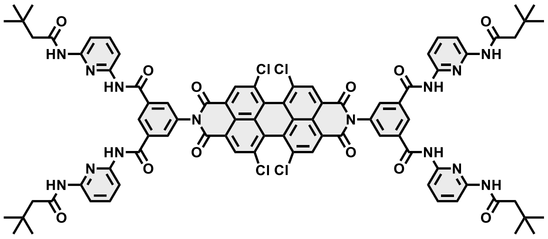

A 10 mL sealable vial was charged with 1,6,7,12-Tetrachloroperylene tetracarboxylic acid dianhydride (10 mg, 18.9 µmol), NH_2-Hamilton (26.4 mg, 47.2 µmol) and $Zn(OAc)_2$ (0.7 mg, 3.8 µmol) under nitrogen atmosphere and dissolved in anhydrous pyridine (2 mL). The reaction was purged by a nitrogen stream for 15 min and then heated to 120 °C for 7 days. Subsequently, the solvent was evaporated under reduced pressure and filtered through a plug of silica (CH_2Cl_2/MeOH 10:1) and further purified by gel-permeation size-exclusion chromatography (BioBeads SX1, $CHCl_3$) yielding the desired product as a dark purple solid (14 mg, 46 %)

^{1}H NMR (500 MHz, THF-*d8*) δ [ppm] = 9.66 (s, 4H), 9.04 (s, 4H), 8.75 (s, 4H), 8.61 (s, 2H), 8.22 (s, 2H), 8.04 – 7.98 (m, 8H), 7.72 (t, J=8.1, 4H), 2.25 (s, 8H), 1.07 (s, 39H)

^{13}C NMR (126 MHz, THF-*d8*) δ [ppm] = 170.74, 164.89, 162.74, 151.74, 151.20, 140.53, 137.29, 137.17, 135.94, 133.00, 132.64, 132.54, 129.58, 127.08, 125.04, 124.66, 110.31, 110.02, 50.67, 31.61

HRMS (MALDI-dctb): m/z**:** Calc.: $[M+Na]^+$ ($C_{84}H_{74}Cl_4N_{14}NaO_{12}$): 1633.4257 found: 1633.4250

IR (ATR): ṽ [cm^{-1}] = 3545, 3426, 3299, 2950, 2867, 1674, 1583, 1443, 1381, 1291, 1236, 1191, 1150, 797

1-(4-Iodophenyl) biuret

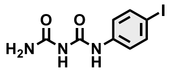

Following a literature procedure,[5] 1-Nitrobiuret (726 mg, 4.90 mmol, 1.3 eq) and 4-Iodoanline (826 mg, 3.77 mmol, 1 eq.) were dissolved in H_2O (10 mL) and heated to reflux for 2.5 h. Subsequently, the mixture was filtered while hot and the collected precipitate was washed with MeOH (5 mL), yielding the title compound as an off-white solid (601 mg, 1.97 mmol, 52 %)

^{1}H NMR (400 MHz, DMSO-d_6) δ [ppm] = 10.05 (s, 1H), 8.92 (s, 1H), 7.67 – 7.58 (m, 2H), 7.35 – 7.24 (m, 2H), 7.02 (br s, 1H), 6.74 (br s, 1H)

^{13}C NMR (101 MHz, DMSO-d_6) δ [ppm] = 155.37, 151.90, 138.06, 137.49, 121.26, 86.31

MS (APPI) m/z: Calc.: $[M+H]^+$ ($C_8H_9IN_3O_2$): 305,9739 found: 305,9734

N-(4-Iodophenyl)cyanuric acid

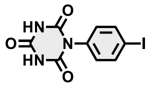

Following a literature procedure,[5] in a dried Schlenk flask under nitrogen atmosphere, sodium (132 mg, 5.74 mmol, 3.5 eq.) was dissolved in dry ethanol (20 mL). Subsequently, 1-(4-Iodophenyl) biuret (500 mg, 1.64 mmol, 1 eq.) and diethylcarbonate (387 mg, 396 mL, 3.28 mmol, 2 eq.) were added and the reaction mixture was stirred at reflux for 24 h. Then, the mixture was allowed to cool to room temperature and the formed white precipitate was collected by vacuum filtration and the residue was washed with toluene (10 mL). The obtained solid was redissolved in water (15 mL) and carefully acidified with aqueous HCl (32 w%) until a precipitate forms, which is again collected via vacuum filtration, yielding the desired cyanuric acid as a white powder (264 mg, 0.797 mmol, 48 %)

^{1}H NMR (400 MHz, DMSO-d_6) δ [ppm] = 11.58 (s, 2H), 7.86 – 7.78 (m, 2H), 7.20 – 7.11 (m, 2H)

^{13}C NMR (101 MHz, DMSO-d_6) δ [ppm] = 149.56, 148.86, 137.70, 134.15, 131.51, 94.83

MS (APPI) m/z: Calc.: $[M+H]^+$ ($C_9H_7IN_3O_3$): 331.9532 found: 331.9529

AB$_2$C carboxylic acid–mesityl-phenylacetylene zinc porphyrin (AcidPor)

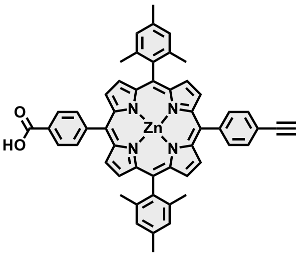

ZnPor(COOMe) (50.0 mg, 59.2 µmol) was dissolved in THF (20 mL) and LiOH (17.0 mg, 710 µmol, 12 eq) dissolved in H_2O (5 mL) was added. The solution was purged with a nitrogen stream for 15 min and the reaction was stirred at room temperature for 20 h under the exclusion of light. Upon full consumption of the starting material, the solvents were removed under reduced pressure. The residue was redissolved in water (25 mL) and carefully acidified with 1 M HCl (ca. 2 mL). The formed precipitate was collected *via* filtration, washed with water until neutrality and dried *in vacuo,* yielding the desired acid porphyrin as a purple powder (46.0 mg, 55.4 µmol, 94 %)

^{1}H NMR (400 MHz, THF-*d*$_8$) δ [ppm] = 8.68 – 8.63 (m, 4H), 8.58 – 8.54 (m, 4H), 8.31 – 8.26 (m, 2H), 8.20 – 8.15 (m, 2H), 8.09 – 8.03 (m, 2H), 7.75 – 7.70 (m, 2H), 7.18 (s, 4H), 3.65 (s, 1H), 2.49 (s, 6H), 1.72 (s, 12H)

^{13}C NMR (101 MHz, THF-*d*$_8$) δ [ppm] = 168.15, 150.91, 150.88, 150.69, 150.56, 145.15, 140.76, 140.01, 138.31, 135.49, 135.42, 132.73, 132.67, 131.22, 131.18, 130.98, 128.69, 128.65, 122.79, 120.23, 120.04, 119.79, 84.63, 79.86, 30.82, 22.13, 21.74

HRMS (MALDI-dctb): m/z: Calc.: $[M]^+$ ($C_{53}H_{40}N_4O_2Zn$) : 828.2443 found: 828.2437

UV/Vis (THF, quali.): 425 nm, 557 nm, 598 nm

Fluorescence (THF): 606 nm (rel. Intens. 100 %), 655 nm (rel. Intens. 81 %)

AB$_2$C *t*Bu-G2-Newkome–mesityl-phenylacetylene zinc porphyrin (G2Por)

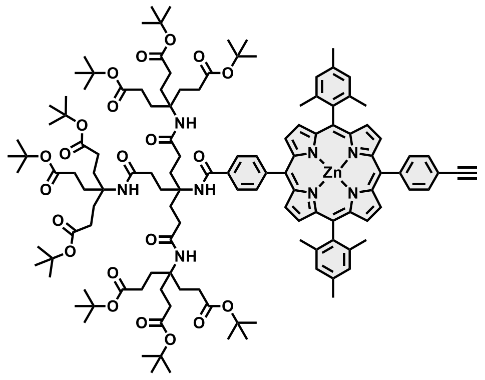

ZnPor(COOH) (43.7 mg, 52.6 µmol), G2-NH_2 (114 mg, 78.9 µmol, 1.5 eq) and HOBt•H_2O (10.5 mg, 68.4 µmol, 1.3 eq) were dissolved in DMF (10 mL) and cooled to 0 °C in an ice bath. Following, a solution of DCC (14.1 mg, 68.4 µmol, 1.3 eq) in DMF (5 mL) was added slowly and the solution was allowed to warm to room temperature. The reaction mixture was stirred at room temperature for 3 d. After full consumption of the starting material was observed as judged by TLC, the mixture was diluted with EtOAc (100 mL), washed with water (2x 200 mL) and the organic phase was dried over $MgSO_4$. The solvent was removed under reduced pressure and the crude residue was purified by filtration through a silica plug (DCM:EtOAc 2:1) and subsequent gel permeation size exclusion chromatography (BioBeads SX1, $CHCl_3$) yielded the desired product as a purple solid (68.9 mg, 30.6 µmol, 58 %)

^{1}H NMR: (600 MHz, THF-*d*$_8$) δ [ppm] = 8.83 (s, 1H), 8.74 – 8.63 (m, 4H), 8.58 – 8.53 (m, 4H), 8.28 – 8.22 (m, 2H), 8.16 – 8.11 (m, 2H), 8.10 – 8.04 (m, 2H), 7.76 – 7.70 (m, 2H), 7.18 (s, 4H), 6.74 (s, 3H), 3.66 (s, 1H), 2.49 (s, 6H), 2.32 – 2.25 (m, 6H), 2.19 – 2.13 (m, 5H), 2.12 – 2.06 (m, 18H), 1.91 – 1.76 (m, 18H), 1.73 (s, 12H), 1.27 (s, 81H)

^{13}C NMR: (151 MHz, THF-*d*$_8$) δ [ppm] = 173.95, 173.16, 173.12, 166.85, 150.87, 150.80, 150.60, 146.99, 145.21, 140.83, 140.01, 138.25, 135.79, 135.48, 135.25, 133.08, 132.61, 131.09, 131.06, 130.97, 128.67, 126.60, 122.74, 120.68, 120.02, 119.64, 84.63, 80.27, 79.86, 59.13, 58.08, 32.36, 32.13, 30.57, 30.37, 28.53, 22.15, 21.74

HRMS (MALDI-dctb): m/z: Calc.: $[M]^+$ ($C_{129}H_{172}N_8O_{22}Zn$): 2249.1878 found: 2249.1864

UV/Vis (THF) (Wavelength (ε [mol*cm^{-1}*L^{-1}])): 425 nm (4.88 × 10^5), 557 nm (1.17 × 10^4), 597 nm (3.91 × 10^3)

Fluorescence (THF): 603 nm (rel. Intens. 100 %), 655 nm (rel. Intens. 88 %)

AB_2C *t*Bu-G2-Newkome–mesityl-cynauric acid zinc porphyrin (*t*BuCyPor)

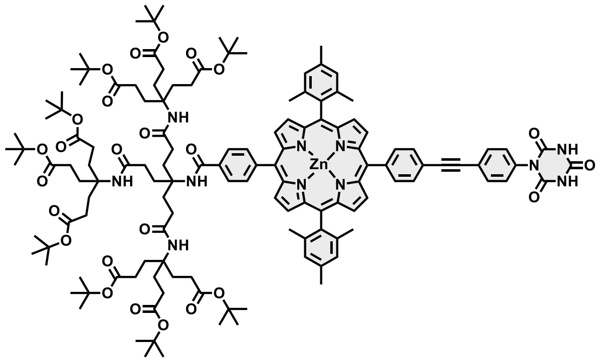

A 20 mL microwave vial was charged with **G2Por** (100 mg, 44.4 µmol), 4-iodophenyl cyanuric acid (14.7 mg, 44.4 µmol), $Pd(PPh_3)_4$ (2.5 mg, 2.22 µmol, 5 mol%), CuI (0.8 mg, 4.44 µmol, 10 mol%) and PPh_3 (1.2 mg, 4.44 µmol, 10 mol%) and set under a nitrogen atmosphere. Further a mixture of THF/NEt_3 (15 mL, 2:1 vv.) was degassed in a ultrasonication bath and added to the reaction vial. The reaction was heated to 80 °C for 16 h. Then, the crude mixture purified by column chromatography (SiO_2, DCM/EtOAc 1:1 → DCM/EtOH 1:1), yielding the desired compound as a purple powder (79 mg, 72 %)

^{1}H NMR (600 MHz, THF-d_8) δ [ppm] = 10.70 (s, 2H), 8.93 (s, 1H), 8.84 – 8.78 (m, 4H), 8.70 – 8.66 (m, 4H), 8.37 (d, *J*=8.2, 2H), 8.28 – 8.18 (m, 4H), 7.93 (d, *J*=8.2, 2H), 7.72 (d, *J*=8.6, 1H), 7.38 (d, *J*=8.6, 2H), 7.30 (s, 4H), 6.86 (s, 3H), 2.44 – 2.39 (m, 6H), 2.32 – 2.25 (m, 6H), 2.25 – 2.15 (m, 18H), 2.01 – 1.94 (m, 18H), 1.85 (s, 12H), 1.39 (s, 81H)

^{13}C NMR (151 MHz, THF-d_8) δ [ppm] = 173.87, 173.14, 166.77, 150.88, 150.87, 150.81, 150.65, 150.33, 149.34, 146.98, 145.10, 140.86, 140.03, 138.24, 135.83, 135.63, 135.56, 135.26, 133.09, 132.76, 132.66, 131.12, 131.06, 130.59, 130.54, 130.40, 128.68, 126.59, 124.60, 123.27, 120.70, 120.13, 119.65, 91.16, 90.49, 80.26, 59.13, 58.07, 32.29, 32.10, 30.59, 30.38, 28.54, 22.17, 21.75

HRMS (MALDI-dctb): m/z:Calc.: $[M]^+$ ($C_{129}H_{172}N_8O_{22}Zn$): 2452.2209 found: 2452.2184

IR (ATR): ṽ [cm^{-1}] = 3322, 2978, 2927, 2851, 1720, 1627, 1523, 1447, 1366, 1311, 1245, 1150, 996

UV/Vis (THF) (Wavelength): 426 nm, 557 nm, 599 nm

Fluorescence (THF): 596 nm (rel. Intens. 87 %), 644 nm (rel. Intens. 100 %)

($CHCl_3$): 605 nm (rel. Intens. 100 %), 655 nm (rel. Intens. 73 %)

AB_2C COOH-G2-Newkome–mesityl-cynauric acid zinc porphyrin (CyPor)

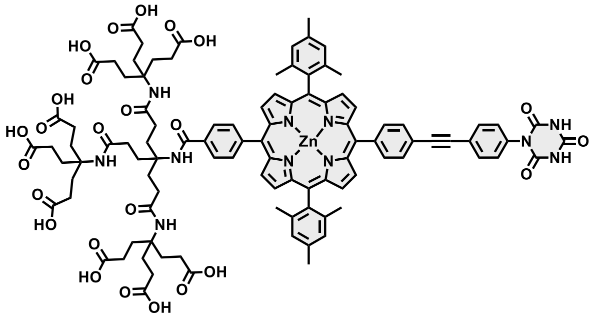

In a 50 mL flask, ***t*BuCyPor** (16.0 mg, 6.50 µmol) was dissolved in formic acid (5 mL) under nitrogen atmosphere. The solution was stirred at room temperature for 3 d under the exclusion of light. Subsequently, the reaction mixture was co-evaporated with toluene (3 x 25 mL) under reduced pressure. The resulting crude solid was redispersed in THF (15 mL) and $Zn(OAc)_2$ (2.4 mg, 13.0 µmol, 2 eq.) was added and the mixture was refluxed for 2 h to re-metalate possible free-base porphyrin. After cooling to room temperature, the precipitate was filtered off and washed with MeOH (10 mL), H_2O (10 mL) and acetone (10 mL). The solid was redissolved in THF with a minimal amount of TFA and quickly precipitated by pentane addition, yielding the product as a dark purple solid. (8.3 mg, 4.26 µmol, 65 %)

^{1}H NMR (600 MHz, THF-d_8) δ [ppm] = 8.83 – 8.72 (m, 2H), 8.69 – 8.65 (m, 2H), 8.62 – 8.55 (m, 4H), 8.27 – 8.21 (m, 2H), 8.17 – 8.08 (m, 4H), 7.86 – 7.80 (m, 2H), 7.64 – 7.58 (m, 2H), 7.29 – 7.23 (m, 6H), 2.53 (s, 6H), 2.35 – 2.30 (m, 6H), 2.27 – 2.16 (m, 6H), 2.14 – 2.04 (m, 24H), 1.95 – 1.85 (m, 24H)

Despite our best efforts, some signals remain broad, therefore exact integration of the signals was not always possible. The signal of the ortho-mesityl protons overlaps with the solvent residue signal of THF-d_8 as judged by HSQC spectra.

^{13}C NMR (151 MHz, THF-d_8) δ [ppm] = 183.06, 175.52, 170.75, 161.28, 160.02, 150.52, 150.50, 150.31, 149.87, 139.47, 135.06, 134.84, 132.43, 130.06, 128.31, 126.66, 120.29, 119.48, 62.35, 59.03, 35.00, 32.57, 31.59, 29.43, 21.67, 21.38

Due to the limited solubility of the compound, not all carbon signals are observable.

HRMS (ESI - negative): m/z Calc.: $[M]^{2-}$ ($C_{102}H_{1103}N_{11}O_{25}Zn$): 972.8214 found: 972.8241

IR (ATR): ṽ [cm^{-1}] = 3297, 3085, 2979, 1704, 1540, 1402, 1204, 1100, 998, 796

Supramolecular Assembly of HamPBI(CyPor)$_2$

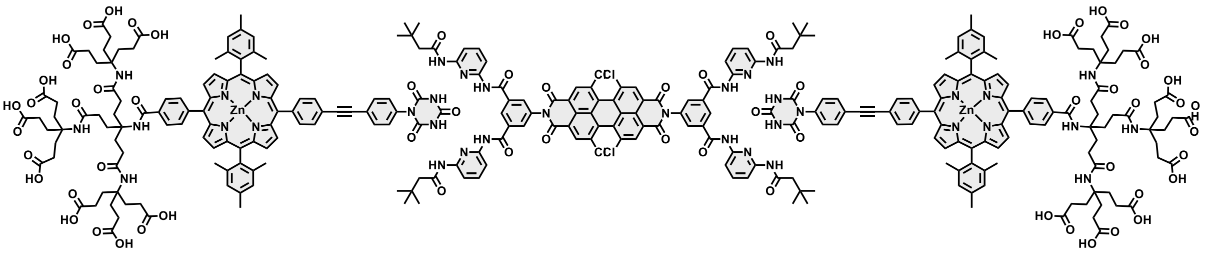

In a 10 mL vial, amphiphile **CyPor** (2.08 mg, 1.07 µmol, 2 eq.) was dissolved in aqueous NaOH solution (960 µL, 10 mM, 18 eq.) and diluted with H_2O to 7.5 mL. Then, **HamPBI** (0.86 mg, 0.53 µmol, 1 eq.) dissolved in 1 mL THF was added. This mixture was placed in a sonication bath until fully dissolved. Then, the mixture was placed in a bath of liquid nitrogen. When fully frozen, the sample was lyophilized, resulting in a spongy red solid, which forms stable dispersions in water.

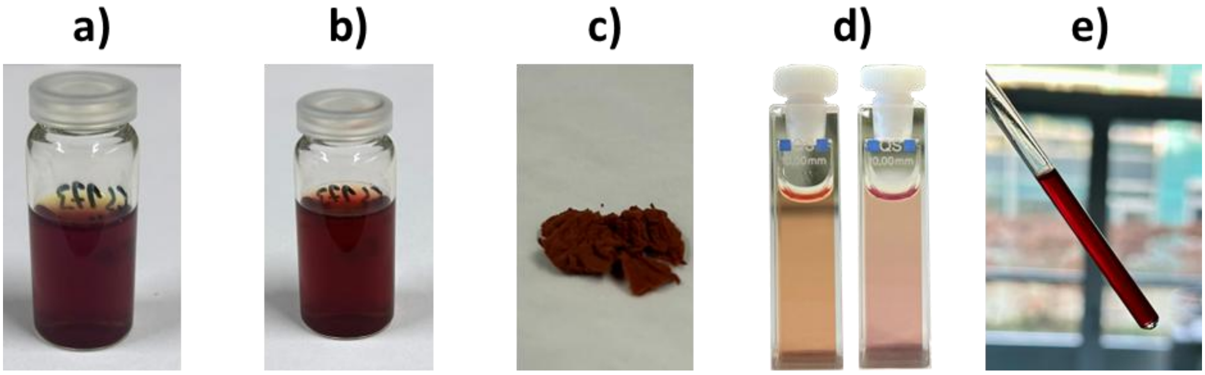

Figure S1. Pictures of the preparation of **HamPBI(CyPor)$_2$**; a) solution of **CyPor** and NaOH in H_2O; b) mixture of **CyPor** in H_2O and **HamPBI** in THF; c) solid obtained after freeze drying; d) pure aqueous solutions of **HamPBI(CyPor)$_2$** (left) and **CyPor** (right); e) solution of **HamPBI(CyPor)$_2$** in D_2O (c_{aprrox} =2 mg/ml).

Theoretical Investigations

Geometries were relaxed using density-functional theory (DFT). The calculations were carried out with the plane-wave code PWScf of the Quantum Espresso software package[6] utilizing the gradient-corrected Perdew-Burke-Ernzerhof (PBE) exchange-correlation functional,[7] Grimme D3 dispersion correction with Becke-Johnson damping,[8],[9] Vanderbilt ultrasoft pseudopotentials,[10] and a plane-wave basis set with a kinetic energy cutoff of 30 Ry. Structures were assumed to be relaxed when a force convergence threshold of 5 meV/Å was reached.

Electronic properties were determined with the ORCA code,[11] using the B3LYP hybrid exchange-correlation functional,[12],[13] the triple-zeta def2-TZVPP basis set,[14] and the RIJCOSX approximation with def2/J auxiliary basis functions.[15] Time-dependent density functional theory (TD-DFT) was used for the calculation of absorption spectra, utilizing the same settings but changing to the CAM-B3LYP long-range corrected hybrid exchange-correlation functional.[16] The lowest 150 vertical transitions were included in the TD-DFT calculations. In **Figure S6**, the transitions were shifted by 73 nm to higher wavelengths to facilitate comparison. Solvation effects in water were taken into account by employing the implicit conductor-like continuum polarization model (C-PCM).[11]

Orbital	**CyPor**	**HamPBI**
HOMO	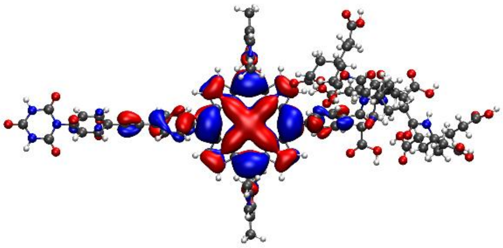	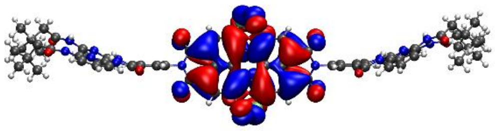
HOMO-1	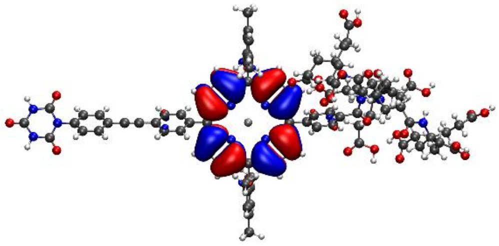	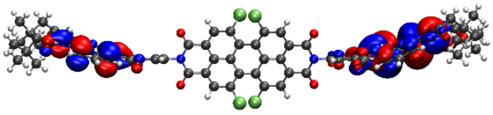
HOMO-2	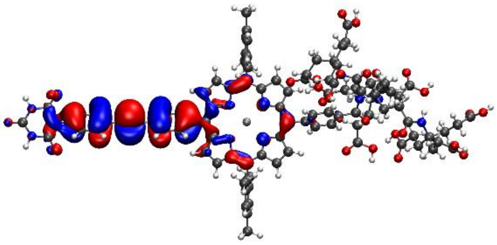	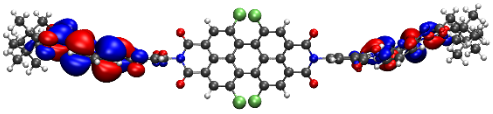
HOMO-3	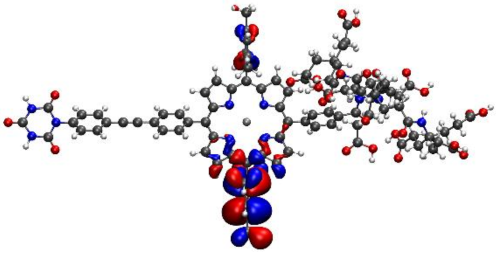	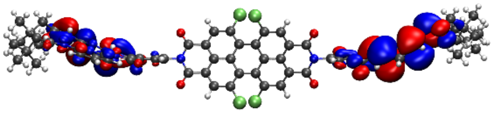

Figure S2. Geometry-optimized structures and HOMOs of **CyPor** and **HamPBI**.

Orbital	**CyPor**	**HamPBI**
LUMO	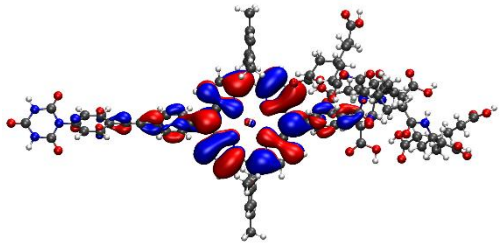	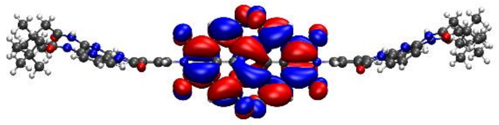
LUMO+1	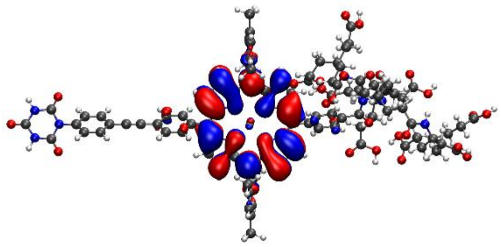	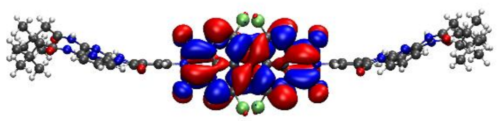
LUMO+2	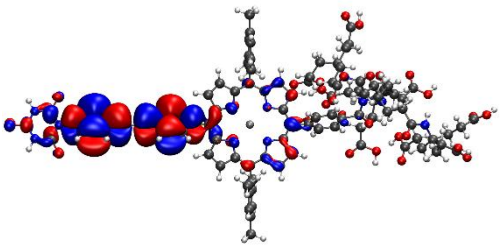	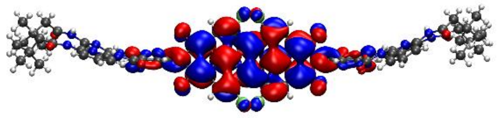
LUMO+3	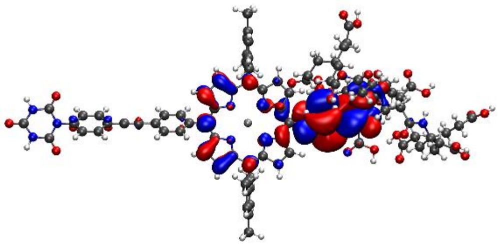	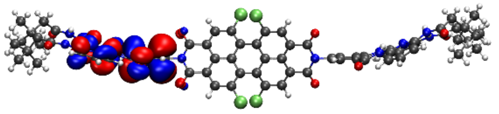

Figure S3. Geometry-optimized structures and LUMOs of **CyPor** and **HamPBI**.

HOMO	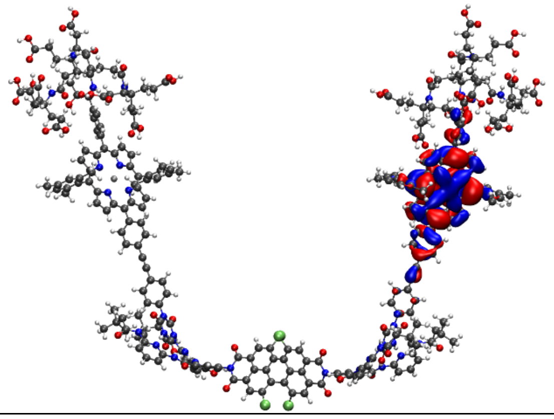
HOMO-1	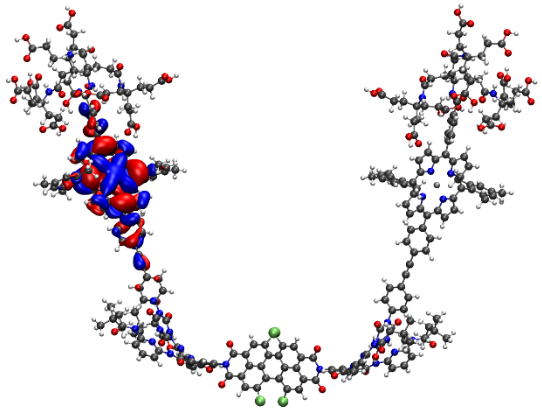
HOMO-2	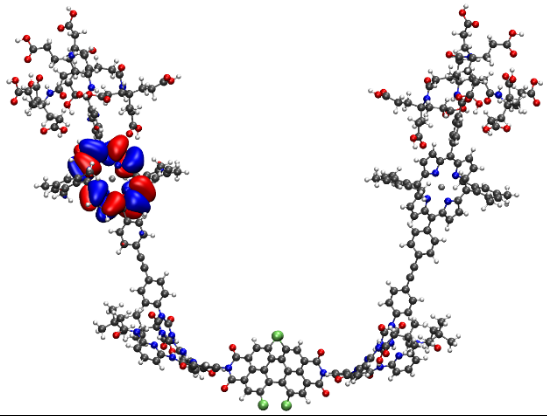
HOMO-3	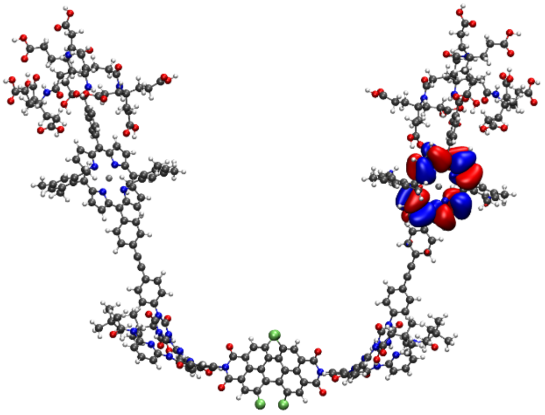

Figure S4. Geometry-optimized structures and HOMOs of **HamPBI(CyPor)$_2$**.

LUMO	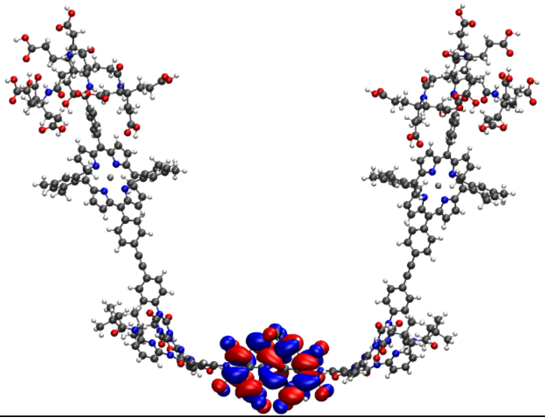
LUMO+1	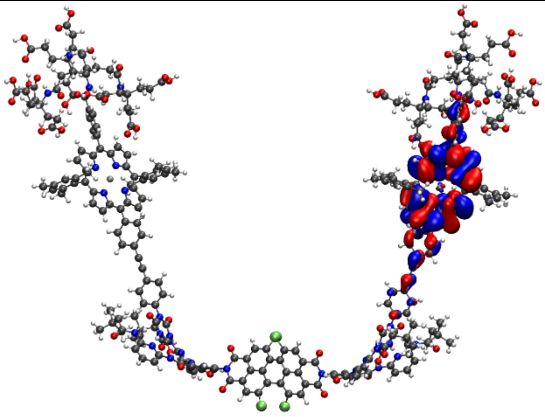
LUMO+2	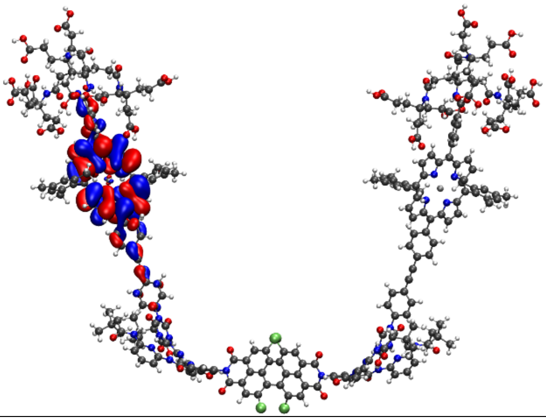
LUMO+3	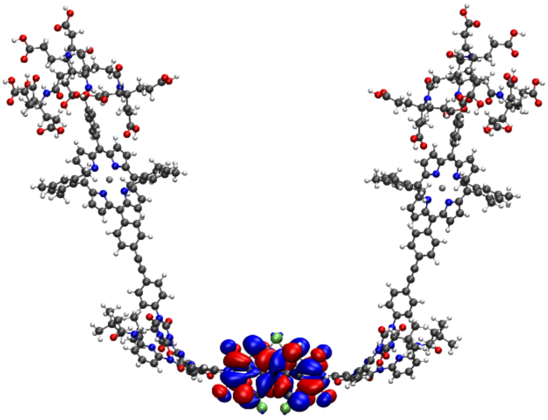

Figure S5. Geometry-optimized structures and LUMOs of **HamPBI(CyPor)$_2$**.

Table S1. Energy values of selected orbitals of **HamPBI**, **CyPor**, **U-HamPBI(CyPor)$_2$**, and **S-HamPBI(CyPor)$_2$**.

	**HamPBI**	**CyPor**	**U-HamPBI(CyPor)$_2$**	**S-HamPBI(CyPor)$_2$**
Orbital	Energy (eV)	Energy (eV)	Energy (eV)	Energy (eV)
HOMO-3	-6.340	-6.403	-5.548	-5.546
HOMO-2	-6.337	-6.128	-5.547	-5.544
HOMO-1	-6.336	-5.585	-5.302	-5.302
HOMO	-6.254	-5.333	-5.301	-5.300
LUMO	-3.820	-2.657	-3.814	-3.814
LUMO+1	-2.552	-2.568	-2.621	-2.615
LUMO+2	-2.195	-1.884	-2.618	-2.610
LUMO+3	-2.083	-1.410	-2.547	-2.546
GAP	2.434	2.676	1.488	1.487

Table S2. Total energy of **U-HamPBI(CyPor)$_2$** and **S-HamPBI(CyPor)$_2$** as well as their energy difference.

	**U-HamPBI(CyPor)$_2$**	**S-HamPBI(CyPor)$_2$**	Difference
Energy	-6697.232194 Ry	-6697.232904 Ry	0.0097 eV

Table S3. TD-DFT excitation energies and oscillator strengths of **CyPor**.

Excited state	Energy (nm)	Energy (eV)	Oscillator strength *f*	Excited state	Energy (nm)	Energy (eV)	Oscillator strength *f*
1	533.7	2.323	0.054	16	284.1	4.364	0.053
2	529.1	2.343	0.002	17	282.2	4.394	0.001
3	362.8	3.417	4.328	18	281.0	4.413	0.008
4	349.3	3.549	2.572	19	280.0	4.428	0.020
5	327.2	3.790	0.102	20	278.2	4.457	0.012
6	321.4	3.858	0.098	21	277.2	4.473	0.144
7	310.1	3.998	0.057	22	274.1	4.524	0.024
8	304.9	4.066	0.004	23	272.4	4.552	0.312
9	303.9	4.080	0.010	24	269.2	4.605	0.002
10	301.7	4.109	0.000	25	268.3	4.621	0.001
11	298.7	4.151	0.084	26	267.0	4.644	0.001
12	295.3	4.199	0.028	27	265.4	4.672	0.011
13	291.2	4.258	0.138	28	262.8	4.718	0.006
14	287.1	4.319	0.093	29	261.1	4.749	0.027
15	285.3	4.346	0.002	30	255.4	4.854	0.009

Table S4. Orbital transitions and their relative contributions to optically active excitations with large oscillator strengths from the TD-DFT calculations of **CyPor**. Only transitions with contributions exceeding 10% are listed.

State	Energy (eV)	Transition	Contribution (%)
1	2.323	HOMO-1 → LUMO+1	37.8
		HOMO → LUMO	53.3
3	3.417	HOMO-1 → LUMO+1	42.4
		HOMO → LUMO	29.6
4	3.549	HOMO-1 → LUMO	41.2
		HOMO → LUMO+1	36.4
5	3.790	HOMO-8 → LUMO	55.2
		HOMO-7 → LUMO	28.9
6	3.858	HOMO-8 → LUMO+1	53.9
		HOMO-7 → LUMO+1	29.4
7	3.998	HOMO-2 → LUMO	30.8
		HOMO-2 → LUMO+2	15.6
		HOMO → LUMO+2	19.4

Table S5. TD-DFT excitation energies and oscillator strengths of **HamPBI**.

Excited state	Energy (nm)	Energy (eV)	Oscillator strength *f*	Excited state	Energy (nm)	Energy (eV)	Oscillator strength *f*
1	476.9	2.600	1.208	16	271.5	4.567	0.378
2	366.6	3.382	0.255	17	271.0	4.576	0.000
3	353.1	3.511	0.030	18	270.2	4.588	0.008
4	325.9	3.805	0.014	19	266.8	4.647	2.077
5	314.5	3.942	0.000	20	266.5	4.652	0.001
6	309.0	4.013	0.001	21	266.3	4.656	0.107
7	308.7	4.016	0.001	22	259.9	4.770	0.059
8	308.1	4.024	0.001	23	256.7	4.830	0.376
9	307.8	4.028	0.000	24	254.4	4.874	0.002
10	301.3	4.115	0.001	25	254.1	4.879	0.001
11	297.6	4.166	0.000	26	253.8	4.885	0.002
12	296.8	4.177	0.003	27	253.6	4.888	0.001
13	295.7	4.193	0.000	28	253.5	4.892	0.009
14	271.8	4.562	0.318	29	253.1	4.898	0.017
15	271.7	4.563	0.076	30	251.0	4.940	0.001

Table S6. Orbital transitions and their relative contributions to optically active excitations with large oscillator strengths from the TD-DFT calculations of **HamPBI**. Only transitions with contributions exceeding 10% are listed.

State	Energy (eV)	Transition	Contribution (%)
1	2.600	HOMO → LUMO	96.2
2	3.382	HOMO-5 → LUMO	84.6
3	3.511	HOMO-6 → LUMO	73.1
14	4.562	HOMO-3 → LUMO+3	13.9
		HOMO-2 → LUMO+3	14.1
16	4.567	HOMO-4 → LUMO+4	23.5
		HOMO-1 → LUMO+4	15.6
19	4.647	HOMO-2 → LUMO+3	14.1

Table S7. TD-DFT excitation energies and oscillator strengths of **HamPBI(CyPor)$_2$**.

Excited state	Energy (nm)	Energy (eV)	Oscillator strength *f*	Excited state	Energy (nm)	Energy (eV)	Oscillator strength *f*
1	533.7	2.323	0.054	16	324.6	3.819	0.015
2	529.1	2.343	0.002	17	324.1	3.826	0.080
3	362.8	3.417	4.328	18	323.8	3.829	0.081
4	349.3	3.549	2.572	19	323.0	3.838	0.096
5	327.2	3.790	0.102	20	322.6	3.843	0.094
6	321.4	3.858	0.098	21	314.4	3.944	0.001
7	310.1	3.998	0.057	22	307.0	4.039	0.077
8	304.9	4.066	0.004	23	306.3	4.048	0.100
9	303.9	4.080	0.010	24	305.8	4.054	0.000
10	301.7	4.109	0.000	25	305.2	4.062	0.000
11	298.7	4.151	0.084	26	304.4	4.073	0.000
12	295.3	4.199	0.028	27	304.2	4.075	0.001
13	291.2	4.258	0.138	28	303.2	4.089	0.000
14	287.1	4.319	0.093	29	303.2	4.090	0.002
15	285.3	4.346	0.002	30	303.1	4.090	0.002

Table S9. Orbital transitions and their relative contributions to optically active excitations with large oscillator strengths from the TD-DFT calculations of **HamPBI(CyPor)$_2$**. Only transitions with contributions exceeding 10% are listed.

State	Energy (eV)	Transition	Contribution (%)
1	2.342	HOMO-3 → LUMO+3	43.1
		HOMO-1 → LUMO+1	53.8
2	2.343	HOMO-2 → LUMO+4	43.3
		HOMO → LUMO+2	53.6
5	2.600	HOMO-4 → LUMO	96.2
10	3.383	HOMO-29 → LUMO	84.5
11	3.452	HOMO-3 → LUMO+3	42.6
		HOMO-1 → LUMO+1	34.1
12	3.461	HOMO-2 → LUMO+4	42.8
		HOMO → LUMO+2	34.4
14	3.577	HOMO-3 → LUMO+1	44.1
		HOMO-1 → LUMO+3	37.2
15	3.580	HOMO-2 → LUMO+2	44.1
		HOMO → LUMO+4	37.1

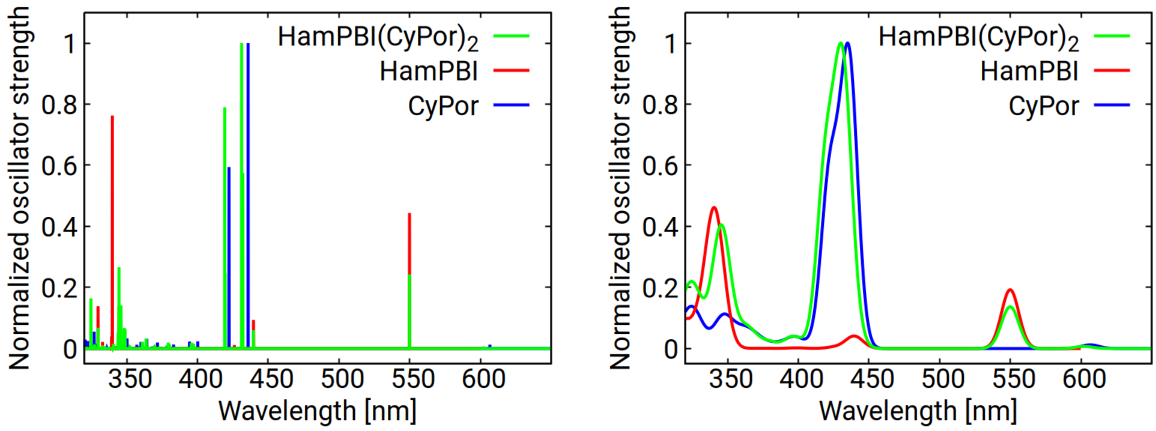

Figure S6. Left: Calculated TD-DFT transitions for **CyPor**, **HamPBI**, and **HamPBI(CyPor)$_2$** overlaid in a line spectrum. Right: The calculated transitions are broadened by a Gaussian function with a width of 7 nm.

ITC Measurement

Isothermal titration calorimetry (ITC) experiments were performed using a Nano ITC-low volume calorimeter from TA instruments. During the titration, 2.46 µL of the guest solution were added over 20 injections with the computer-controlled 50 µL syringe into the corresponding host solution. Experiments were performed with a stirring rate of 350 rpm at 25 °C. Experimental data was analyzed using the AFFINImeter ITC software. For each titration a blank experiment of the addition of ***t*BuCyPor** into the solvent was subtracted from the data.

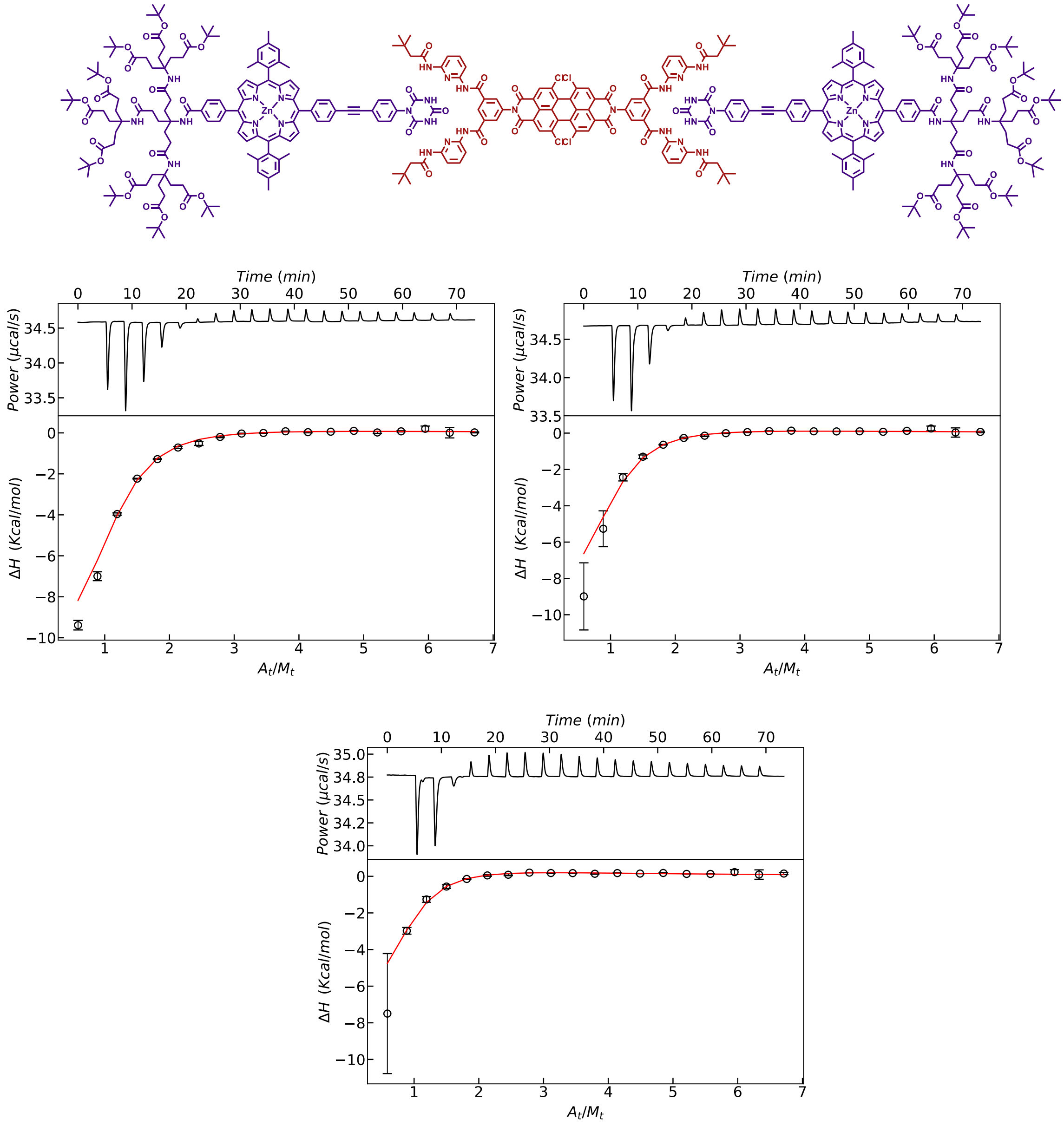

Figure S7. ITC data (each: top: thermogram; bottom: fitting (red line) of the experimental data (black dots) based on a 1:2 binding model) **of HamPBI** and ***t*BuCyPor** in anhydrous $CHCl_3$.

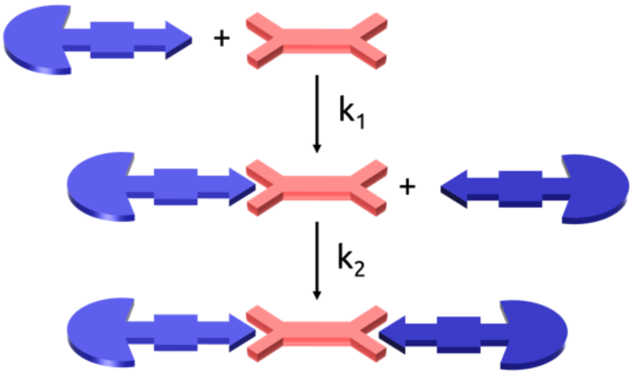

Figure S8. Schematic representation of the stepwise binding of ***t*BuCyPor** to **HamPBI**.

Table S10. Overview over the biding constants obtained from applying a 2:1 binding model.

Reaction	K_a [M^{-n}]	STD (K)
Free Species ↔ $H^{PBI}C^{Por}$	8.9980×10^4	2.2817×10^3
$H^{PBI}C^{Por}$↔ $H^{PBI}(C^{por})_2$	4.0791×10^3	2.0385×10^2

	k_1 ($CHCl_3$)	k_2 ($CHCl_3$)
ΔH / kcal	-11,1	-2,4
ΔH / kJ	-46,4424	-10,0416
K_a	$9{,}00\times10^4$	$4{,}08\times10^3$
K_d	$1{,}11\times10^{-5}$	$2{,}45\times10^{-4}$
ΔG / J	$-2{,}83\times10^4$	$-2{,}06\times10^4$
ΔG / cal	$-6{,}76\times10^3$	$-4{,}93\times10^3$
ΔS / kJ	$-6{,}09\times10^{-2}$	$3{,}54\times10^{-2}$
**ΔS / kcal**	**$-1{,}46\times10^{-2}$**	**$8{,}47\times10^{-3}$**
ΔG / kJ	$-2{,}83\times10^1$	$-2{,}06\times10^1$
**ΔG / kcal**	**$-6{,}76\times10^0$**	**$-4{,}93\times10^0$**
ΔS / cal	$-1{,}46\times10^1$	$8{,}47\times10^0$

IR Spectroscopy

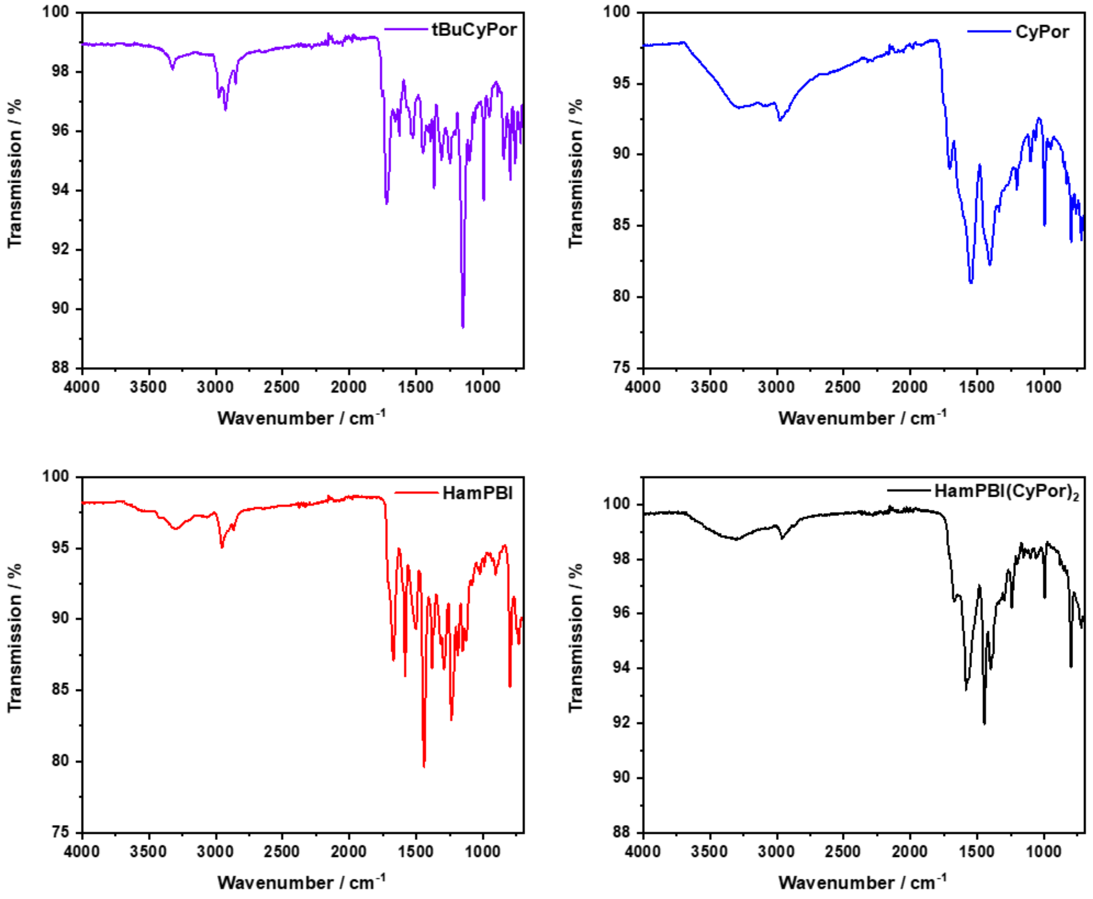

Figure S8. ATR-IR spectra of **tBuCyPor** (purple), **CyPor** (blue), **HamPBI(CyPor)$_2$** (black) and **HamPBI** (red).

Comparing the obtained normalized IR spectra shows, that the spectrum of **HamPBI(CyPor)$_2$** presents the superimposed peaks of the single chromophores, without any significant changes being observed. This can either be interpreted as the absence of assemblies in the solid state, or that the bands corresponding to the hydrogen bonding motif overlapping with other signals and can therefore not be observed.

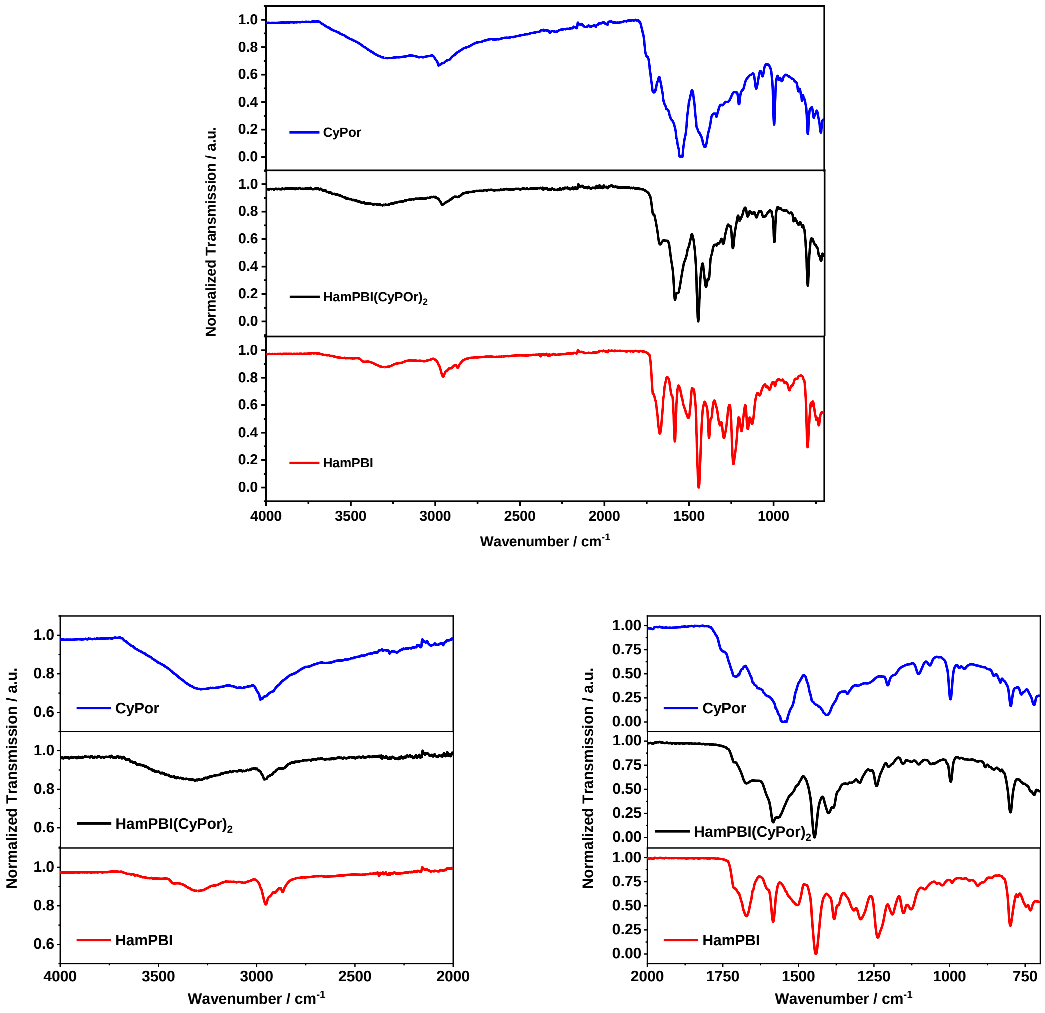

Figure S9. Normalized ATR-IR spectra of **CyPor** (blue), **HamPBI(CyPor)$_2$** (black) and **HamPBI** (red); bottom partial spectra.

DLS Measurement

DLS measurements were carried out in phosphate buffered solutions and in pure aqueous solutions. The used solvents were filtered through a micro filter prior to use. All samples were rather polydisperse (PdI in the range of 0.3-0.4). For **HamPBI(CyPor)₂** a trend towards larger sizes was observed when the samples was diluted, while **CyPor** stayed in the same size range. The correlation coefficients show an almost mono-exponential decay, indicating that spherical particles are present.

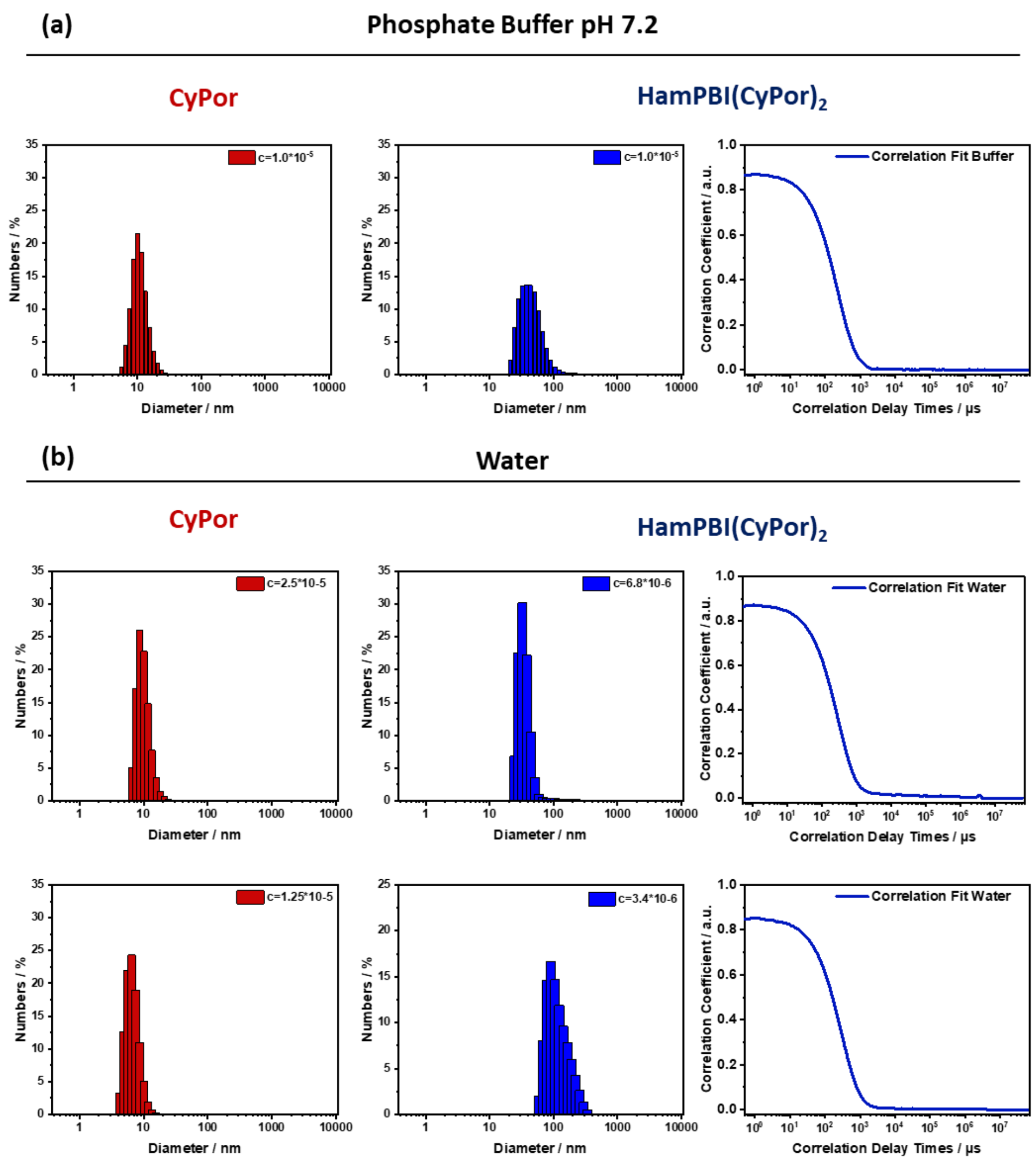

Figure S10. DLS measurements in (a) phosphate buffered solutions (pH 7.2) and (b) pure aqueous solutions of **CyPor** and **HamPBI(CyPor)₂** (concentrations as indicated) at room temperature with the corresponding correlation data of the **HamPBI(CyPor)₂** measurements.

Cryo-TEM Imaging

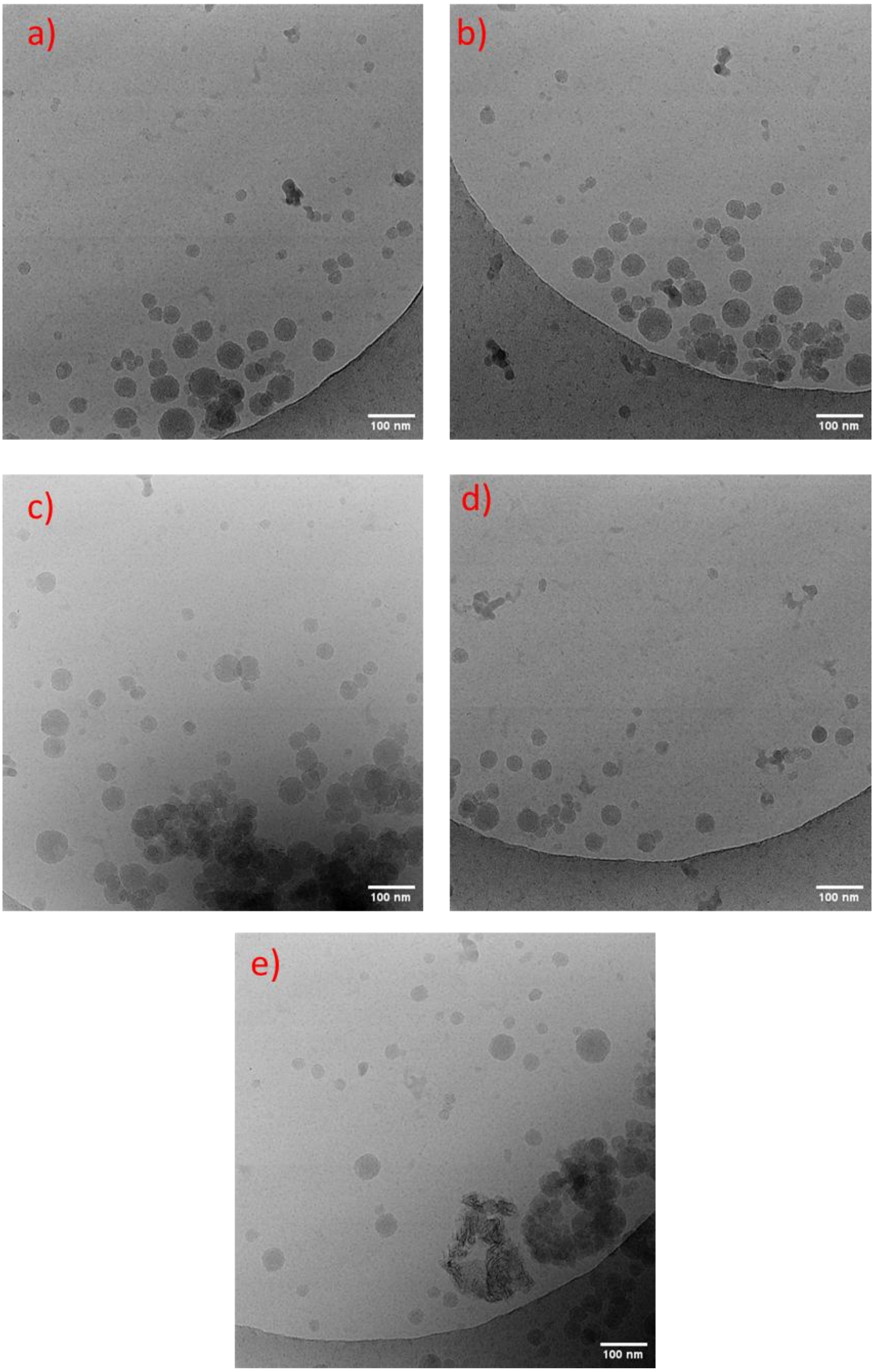

Figure S11. Selection of further representative cryo-TEM micrographs (a-b) highly agglomerated areas (c), less covered areas (d), and highly agglomerated as well as undissolved crystalline areas (e).

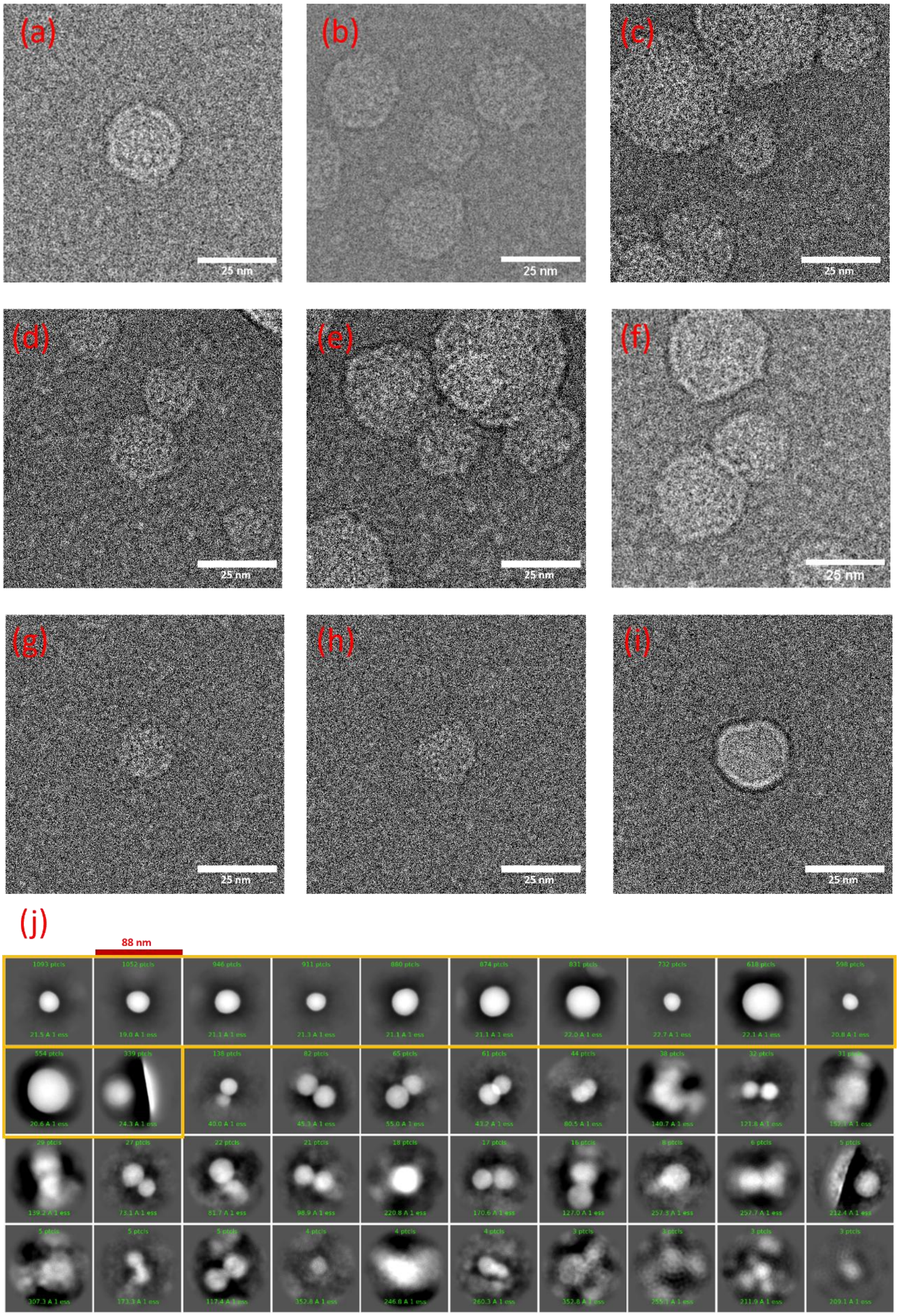

Figure S12. a-h) selection of further single particle images, i) image of a differently shaped particle, which is ascribed to an ethane artifact from the vitrification to visualize the difference with the observed aggregates j) 2D-classification through automated picking of particles with the amounts of particles in each class, the highlighted images were used for the size distribution, as the remaining particles classes show more than particle and only a small number of particles were sorted into them.

Photophysical Investigations

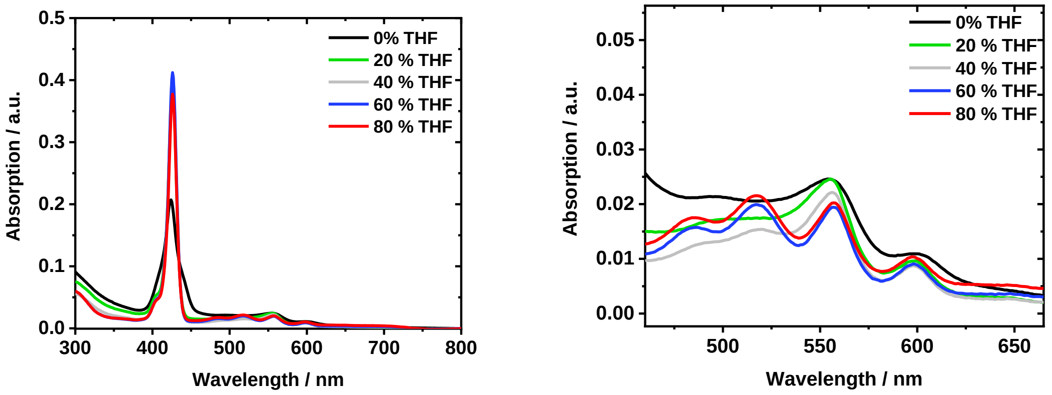

Figure S13. UV/Vis absorptions spectra of **HamPBI(CyPor)₂** in H_2O with different amounts of THF (c = 6×10^{-6} M).

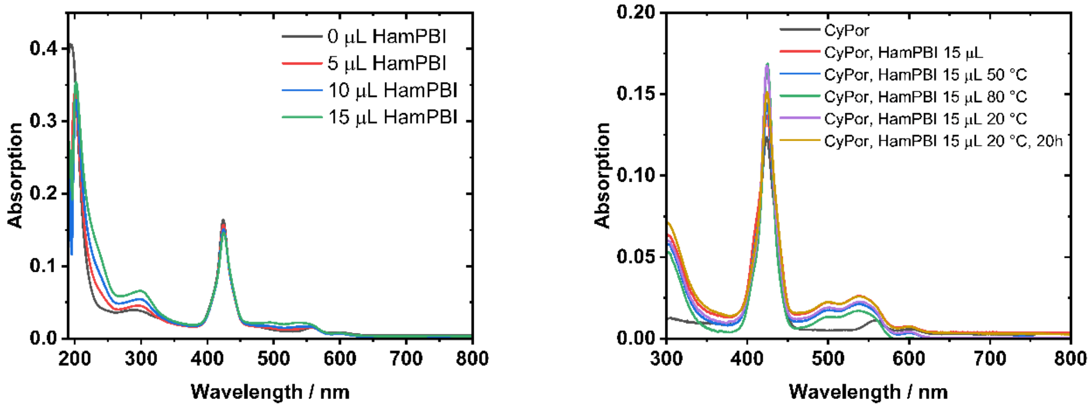

Figure S14. Steady-state titration measurements of **CyPor** 2 x 10^{-6} M (3 x 10^{-3} L) in water using a 2 x 10^{-4} M **HamPBI** solution in THF (left) and temperature and time dependent measurements of the 2:1 **CyPor**:**HamPBI** mixture (right). This was achieved by adding a 2 x 10^{-4} M **HamPBI** THF solution to **CyPor** 2 x 10^{-6} M (3 x 10^{-3} L) in water.

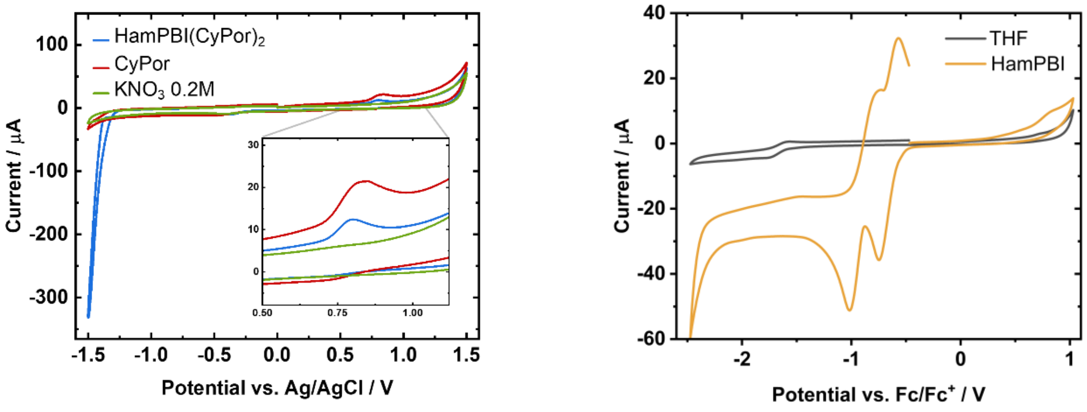

Figure S15. Cyclic voltammogram of **CyPor** and **HamPBI(CyPor)₂** in an aqueous 0.2 M KNO_3 solution (left) measured using a glassy carbon electrode as working electrode, a Pt wire as counter electrode and an Ag/AgCl as reference electrode.

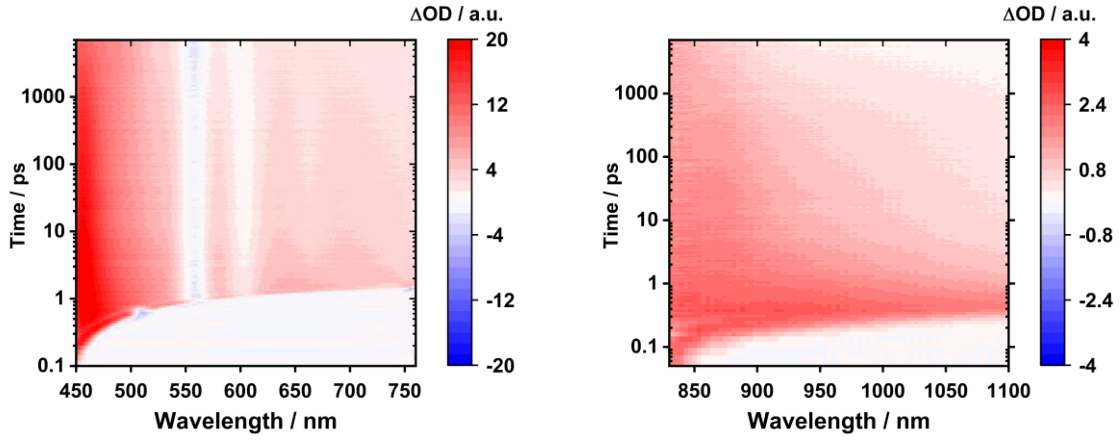

Figure S16. Differential absorption spectra in the visible (left) and near-infrared region (right) obtained upon 430 nm femtosecond photoexcitation (0.4 µJ) of **CyPor** (5×10^{-5} M) in water with time delays between 0 and 7500 picoseconds.

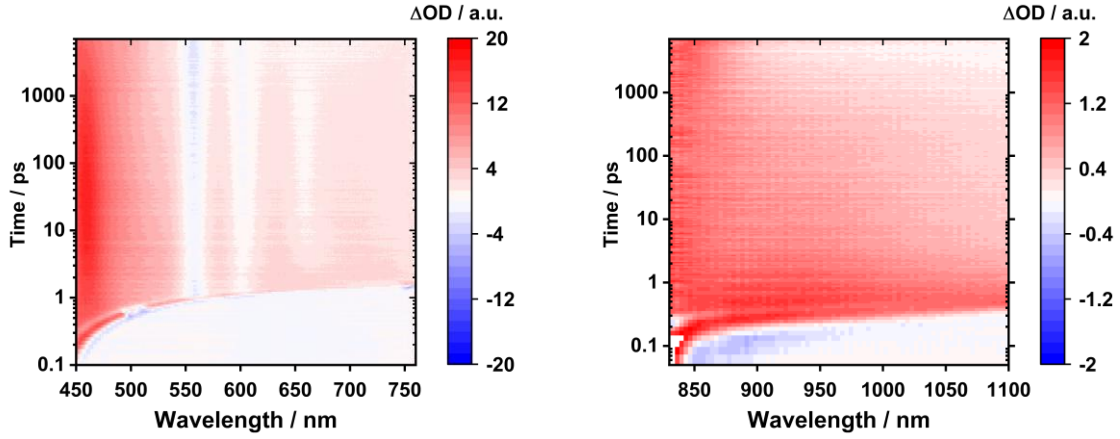

Figure S17. Differential absorption spectra in the visible (left) and near-infrared region (right) obtained upon 430 nm femtosecond photoexcitation (0.4 µJ) of **CyPor** (2.5×10^{-5} M) in 50 vol% THF in water with time delays between 0 and 7500 picoseconds.

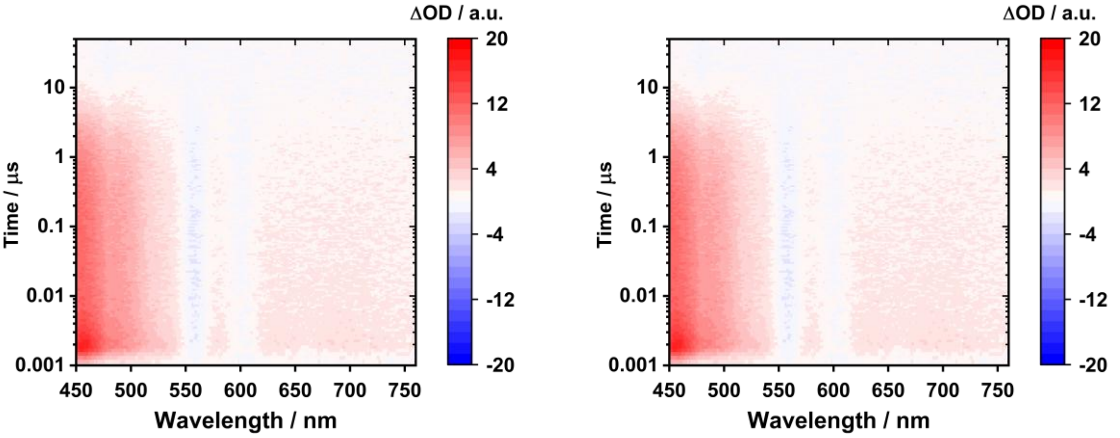

Figure S18. Differential absorption spectra in the visible region of **CyPor** in water (5×10^{-5} M) (left) and 50 vol% THF in water (2.5×10^{-5} M) (right) obtained upon 430 nm femtosecond photoexcitation (0.4 µJ) with time delays between 0 and 50 microseconds.

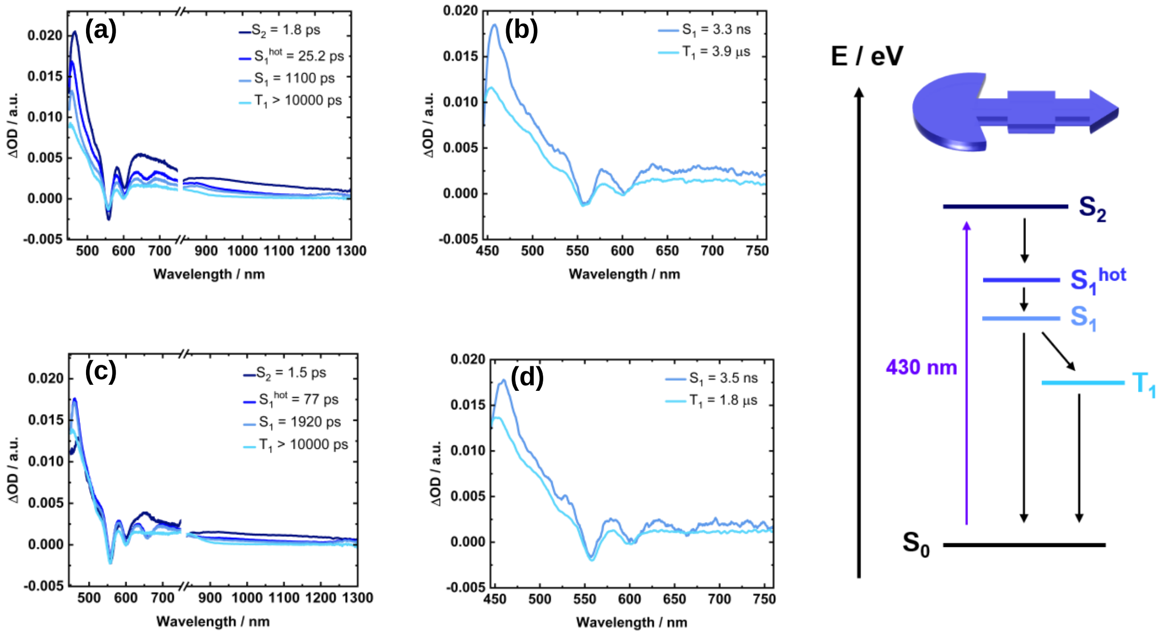

Figure S19. Evolution associated spectra obtained through deconvolution of the fsTA data using a sequential model (photoexcitation at 430 nm) of **CyPor** in (a) water (Figure S13) and (c) 50 vol% THF in water (Figure S14), the respective nsTA data in (b) water and (d) 50 vol% THF in water (Figure S15) and the proposed deactivation pathway is depicted on the right.

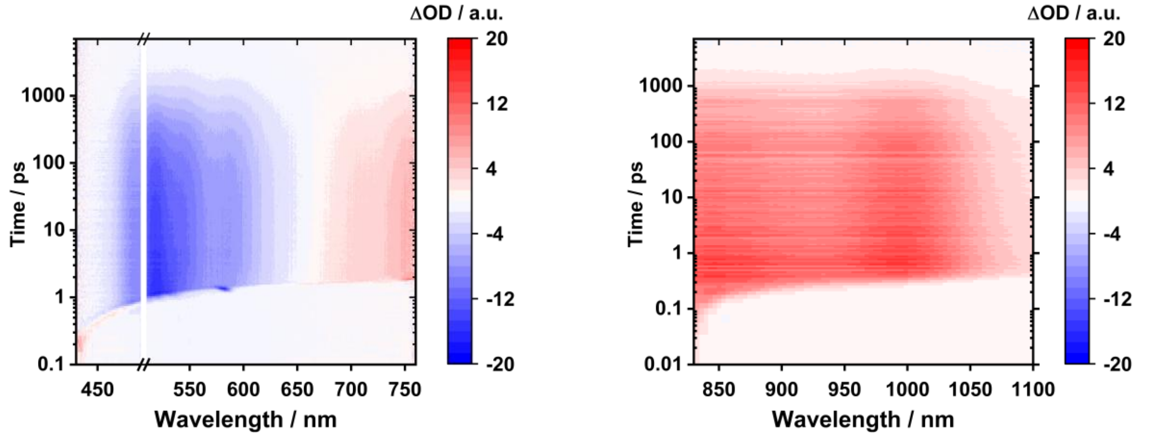

Figure S20. Differential absorption spectra in the visible (left) and near-infrared region (right) obtained upon 500 nm femtosecond photoexcitation (0.6 µJ) of **HamPBI** in THF with time delays between 0 and 7500 picoseconds.

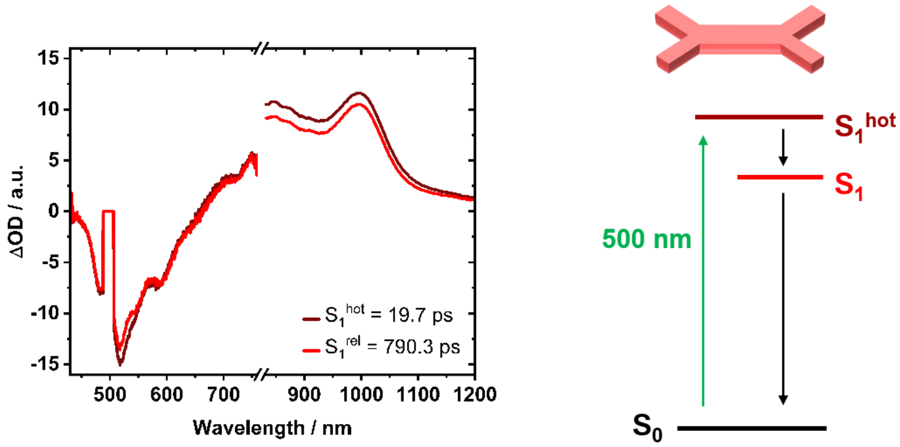

Figure S21. Evolution associated spectra obtained through deconvolution using a sequential pathway of the fsTA data (Figure S20) of **HamPBI** in THF (left) and the proposed deactivation pathway (right).

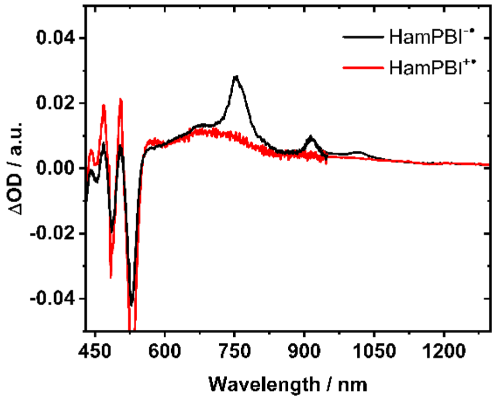

Figure S22. Steady-state absorption measurement of the oxidation of 1 × 10^{-3} M of **HamPBI** in 0.2 M $TBAPF_6$ in THF using a Pt mesh as working electrode, a Pt wire as counterelectrode and a silver wire as quasi reference electrode. The applied potentials were -0.66 V for the radical anion and 0.8 V for the radical cation, which was determined through electrochemical measurements (Figure S13).

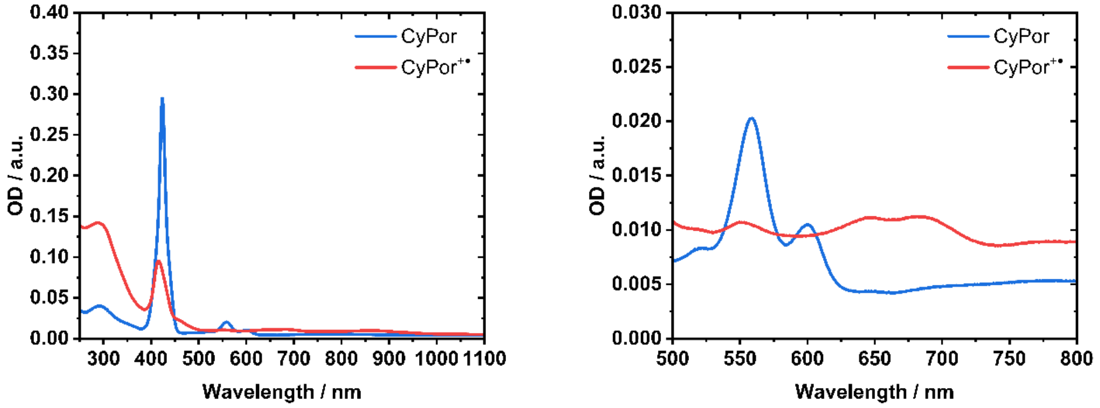

Figure S23. Steady-state absorption measurement of the chemical oxidation of 7 × 10^{-6} M **CyPor** in water using ceric ammonium nitrate. Spectrolectrochemical measurements did not show any spectral features for the radical cation which is why we chose to oxidate it chemically.

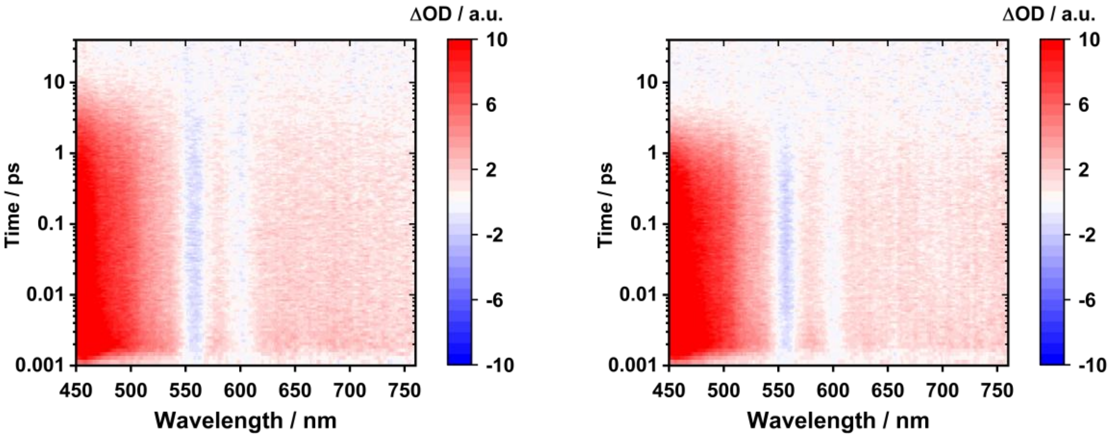

Figure S24. Differential absorption spectra in the visible region of **HamPBI(CyPor)₂** in water (5 × 10^{-5} M) (left) and 50 vol% THF in water (2.5 × 10^{-5} M) (right) obtained upon 430 nm femtosecond photoexcitation (0.4 μJ) with time delays between 0 and 50 microseconds.

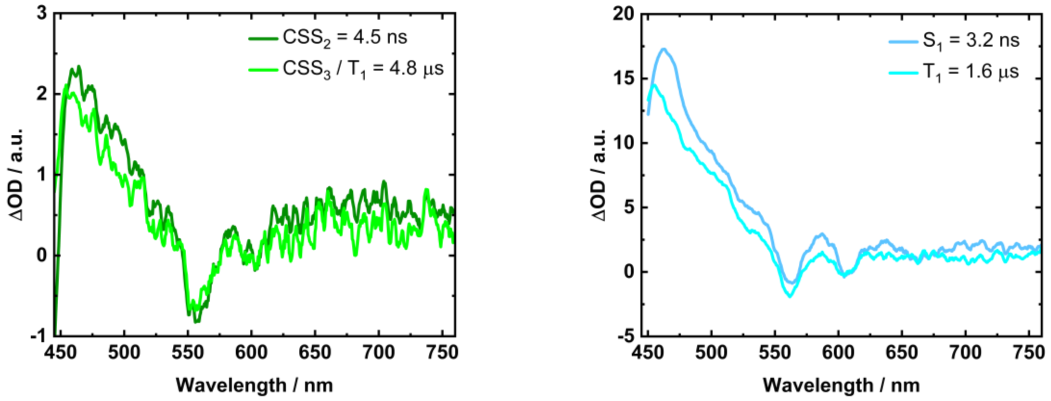

Figure S25. Evolution associated spectra obtained through deconvolution of the nsTA data of **HamPBI(CyPor)₂** (Figure S18) in water (left) and 50 vol% THF in water (right).

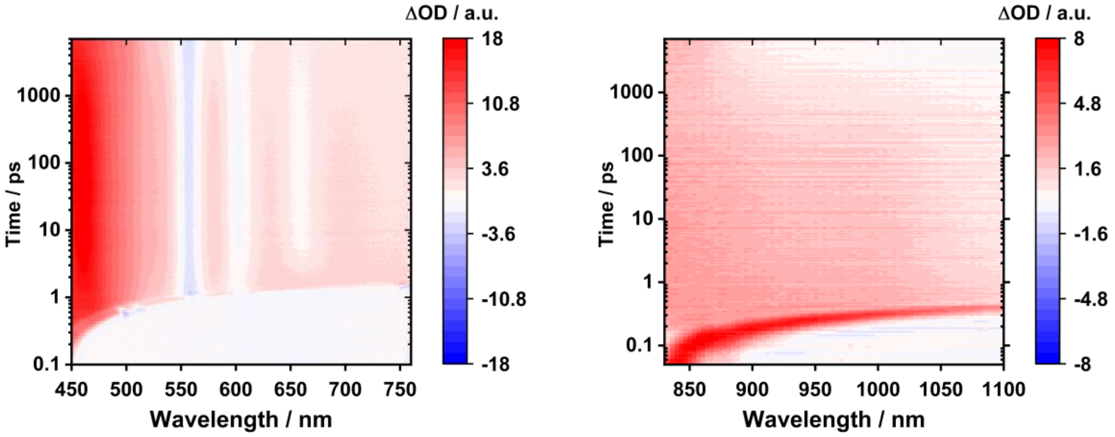

Figure S26. Differential absorption spectra in the visible (left) and near-infrared region (right) obtained upon 430 nm femtosecond photoexcitation (0.4 μJ) of **HamPBI(CyPor)₂** (2.5 × 10^{-5} M) in 50 vol% THF in water with time delays between 0 and 7500 picoseconds.

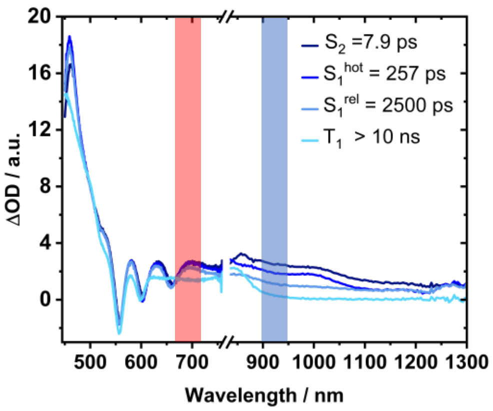

Figure S27. Evolution associated spectra obtained through deconvolution of the fsTA data (Figure S26) of **HamPBI(CyPor)₂** (2.5 × 10^{-5} M) in 50 vol% THF in water using a sequential model (photoexcitation at 430 nm). The marked regions correspond to the regions, where the radical cation of **CyPor** (red) and radical anion of **HamPBI** (blue) were visible before 50 vol% THF addition.

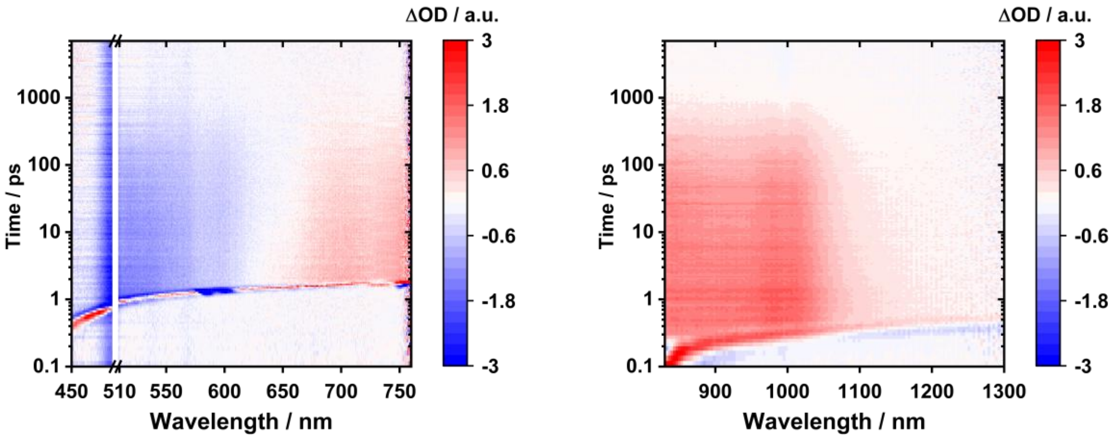

Figure S28. Differential absorption spectra in the visible (left) and near-infrared region (right) obtained upon 500 nm femtosecond photoexcitation (0.4 µJ) of **HamPBI(CyPor)₂** (2.5 × 10^{-5} M) in 50 vol% THF in water with time delays between 0 and 7500 picoseconds.

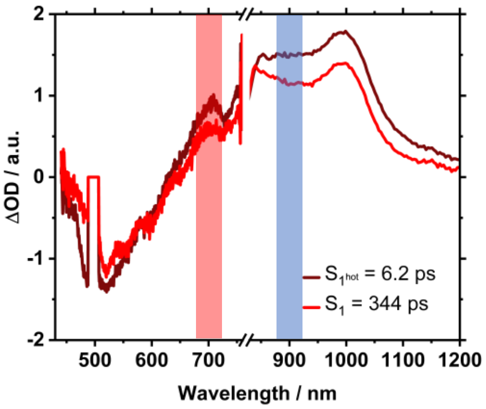

Figure S29. Evolution associated spectra obtained through deconvolution of the fsTA data (Figure S28) of **HamPBI(CyPor)₂** (2.5 × 10^{-5} M) in 50 vol% THF in water using a sequential model (excitation at 500 nm). The marked regions correspond to the regions were the radical cation of **CyPor** (red) and radical anion of **HamPBI** (blue) were visible before 50 vol% THF addition.

NMR Spectroscopy

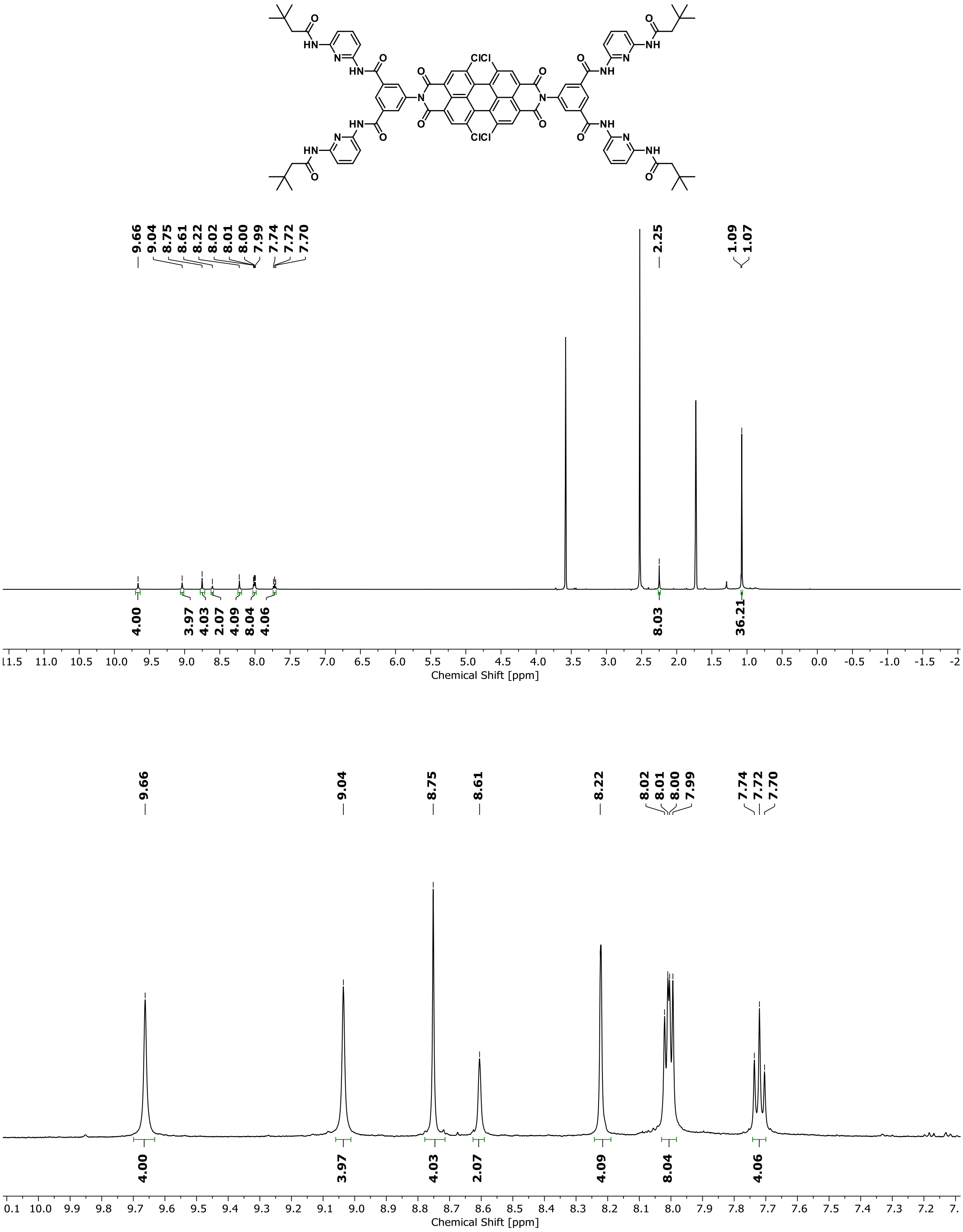

Figure S30. ^{1}H NMR (500 MHz, THF-d_8, *rt*) of **HamPBI** (Top: whole spectrum; bottom: aromatic region)**.**

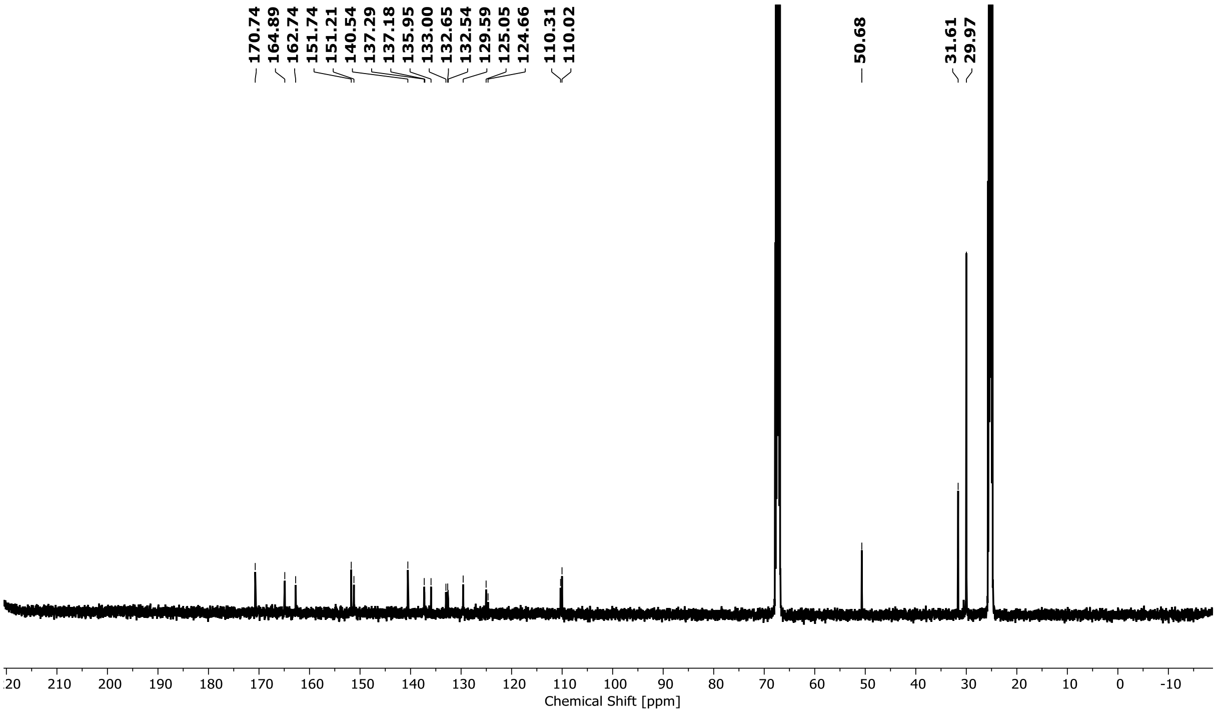

Figure S31. ^{13}C NMR (126 MHz, THF-d8, r.t.) of **HamPBI**.

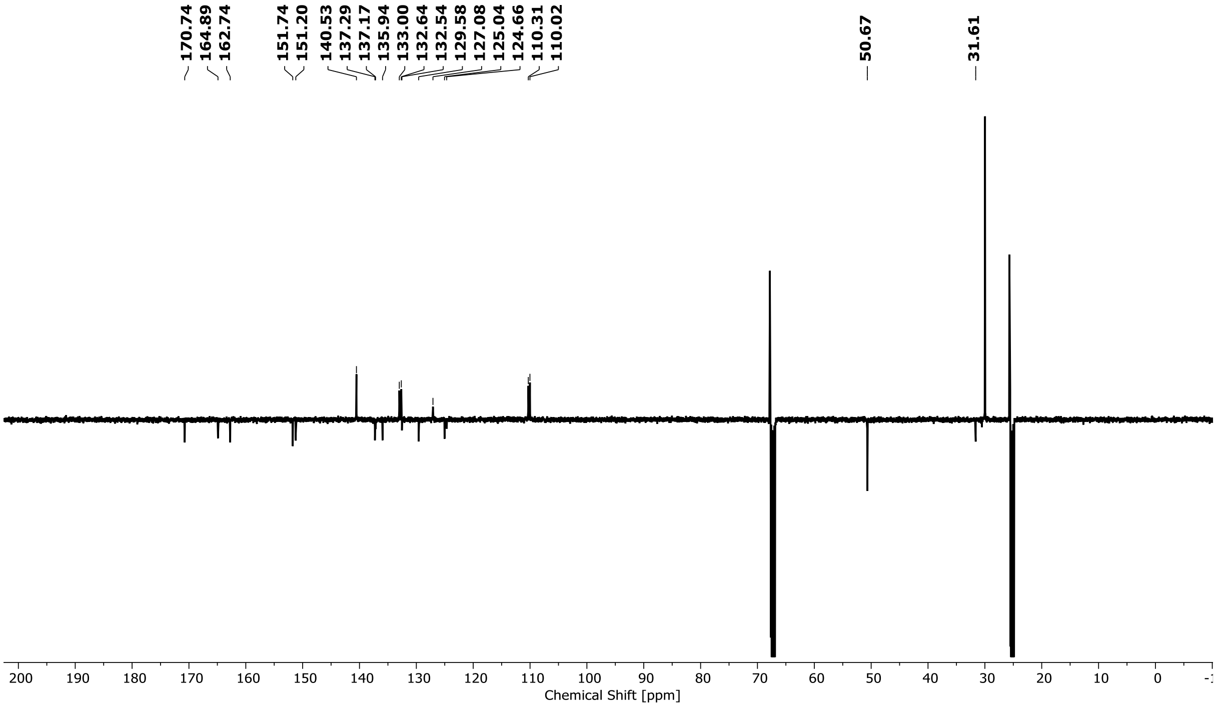

Figure S32. DEPTq135 NMR (126 MHz, THF-d_8, rt) of **HamPBI**.

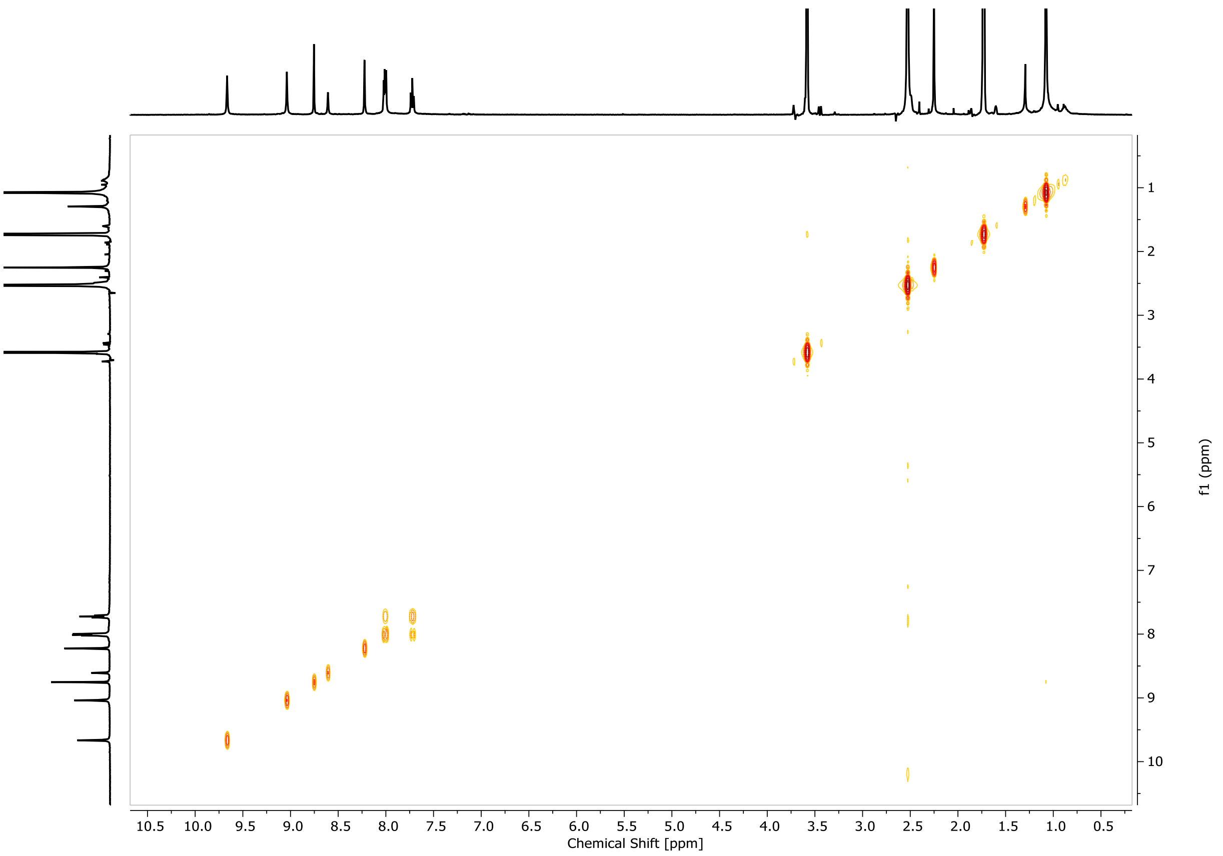

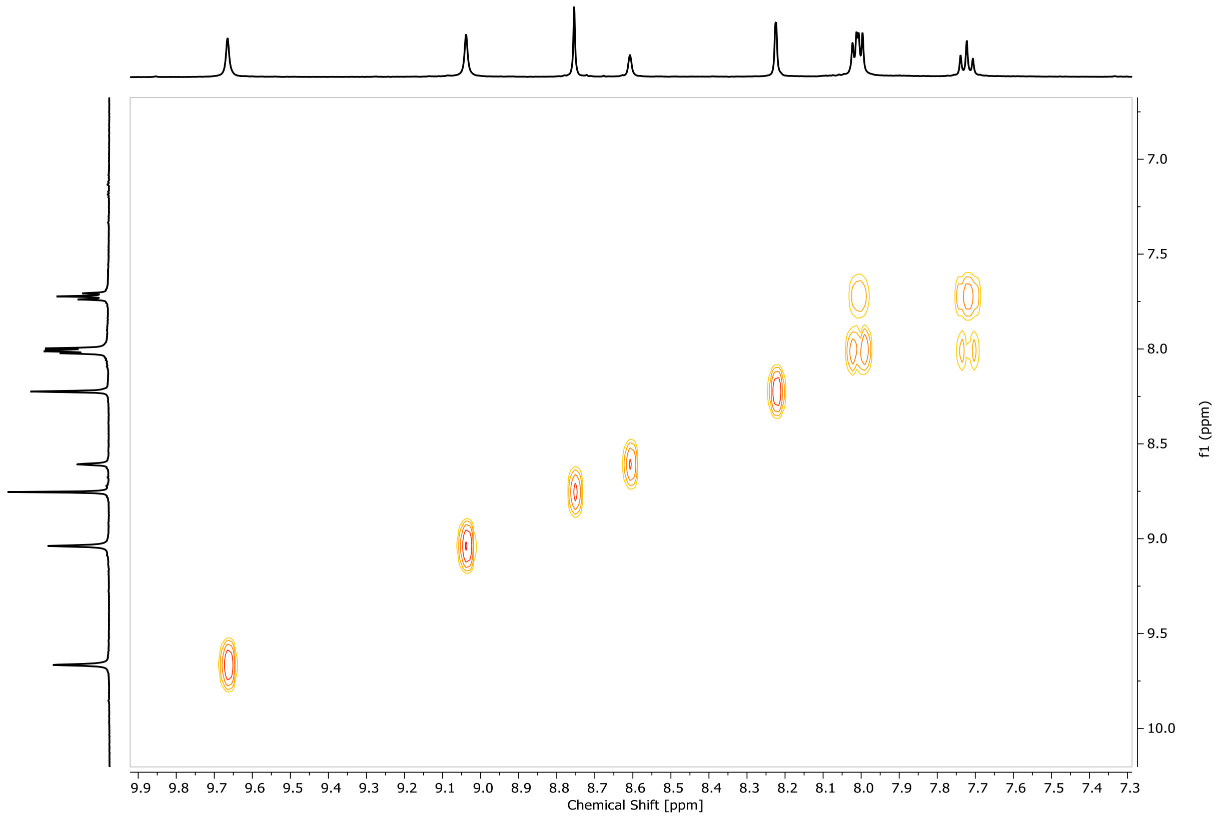

Figure S33. COSY NMR (500 MHz, THF-d_8, rt) of **HamPBI**.

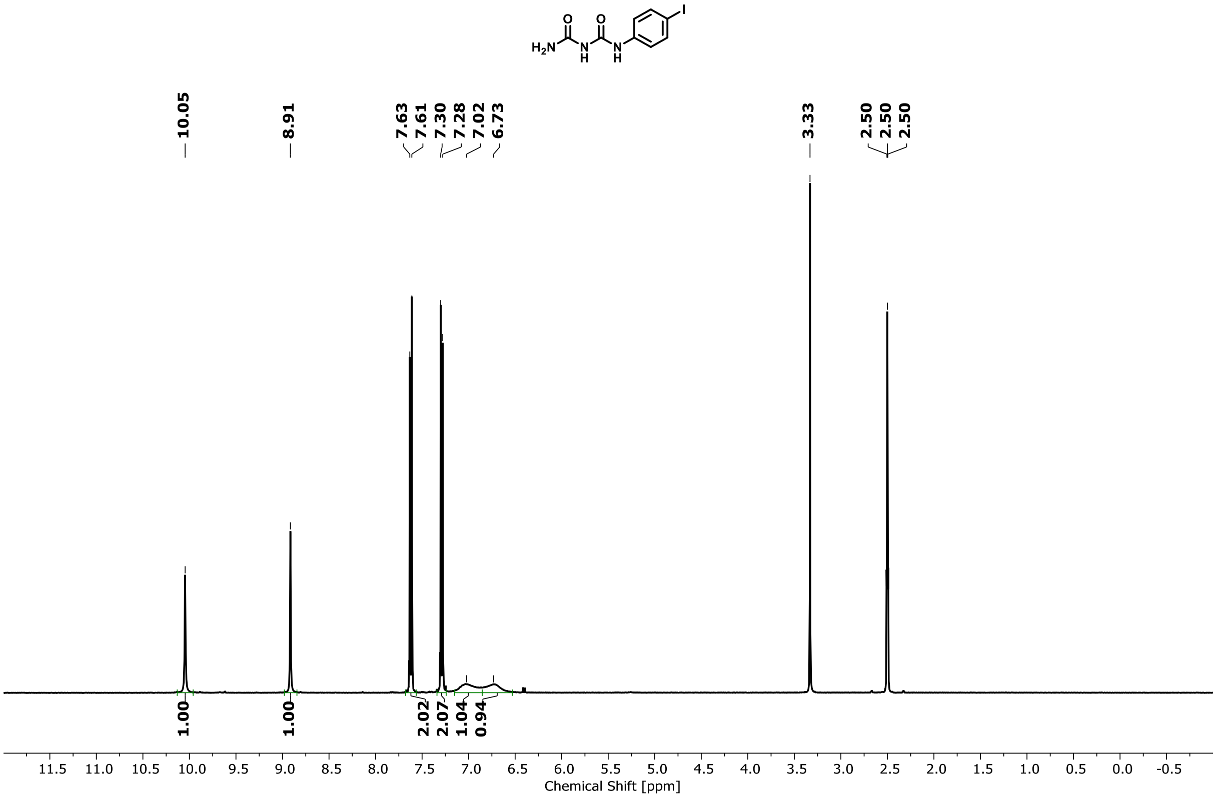

Figure S34. ^{1}H NMR (500 MHz, DMSO-d_6, rt) of **1-(4-Iodophenyl) biuret**.

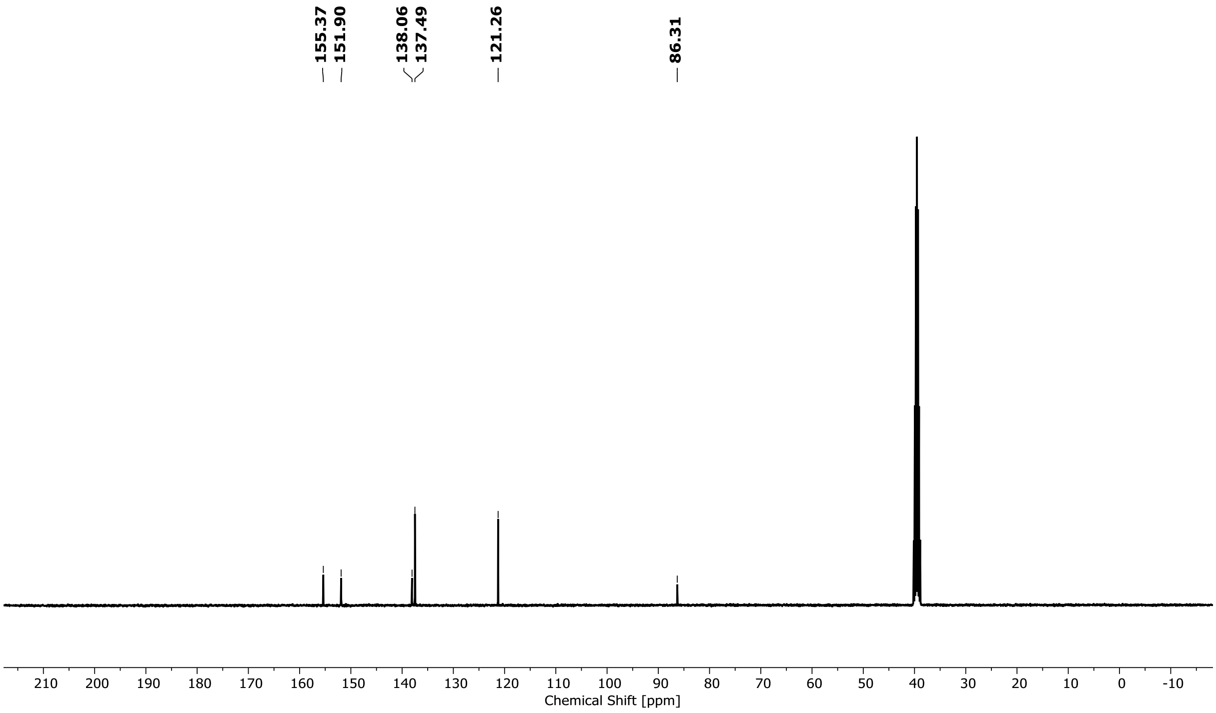

Figure S35. ^{13}C NMR (101 MHz, DMSO-d_6, rt) of **1-(4-Iodophenyl) biuret**.

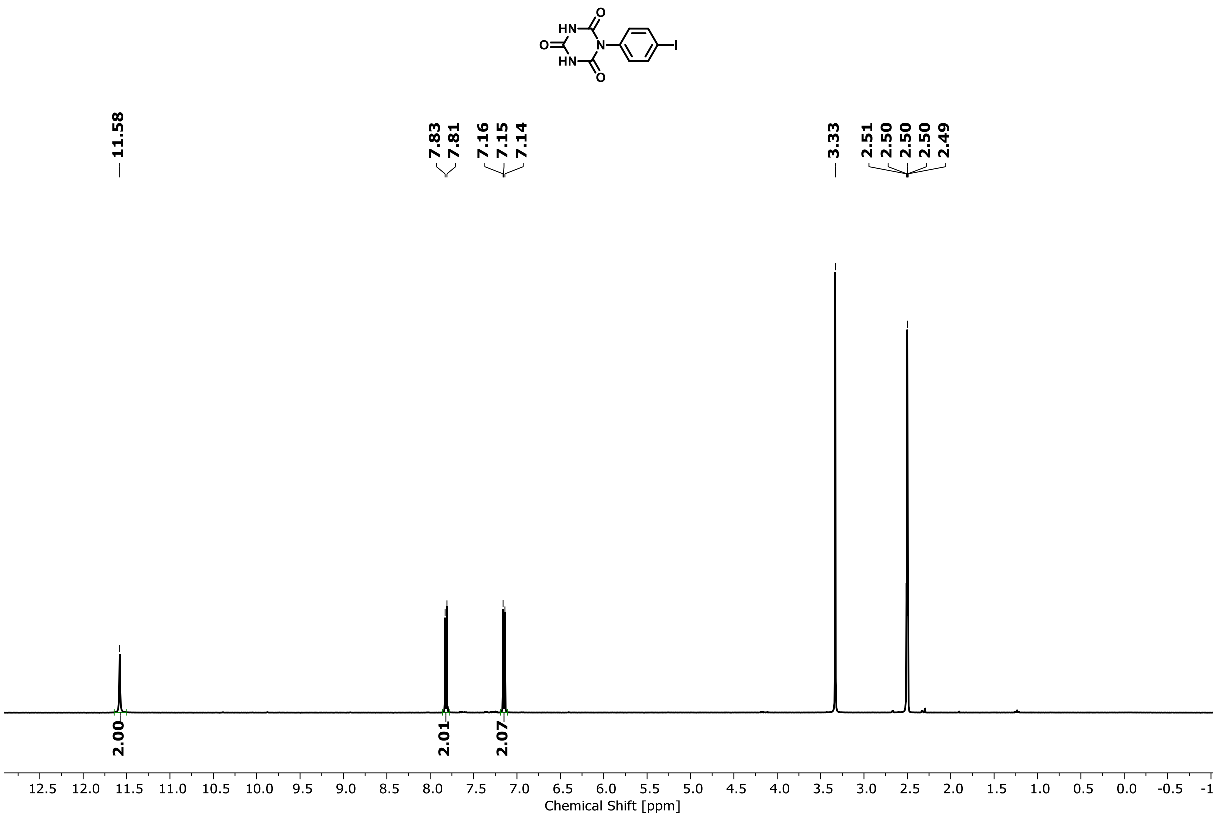

Figure S36. ^{1}H NMR (400 MHz, DMSO-d_6, rt) of **4-Iodo phenyl cyanuric acid**.

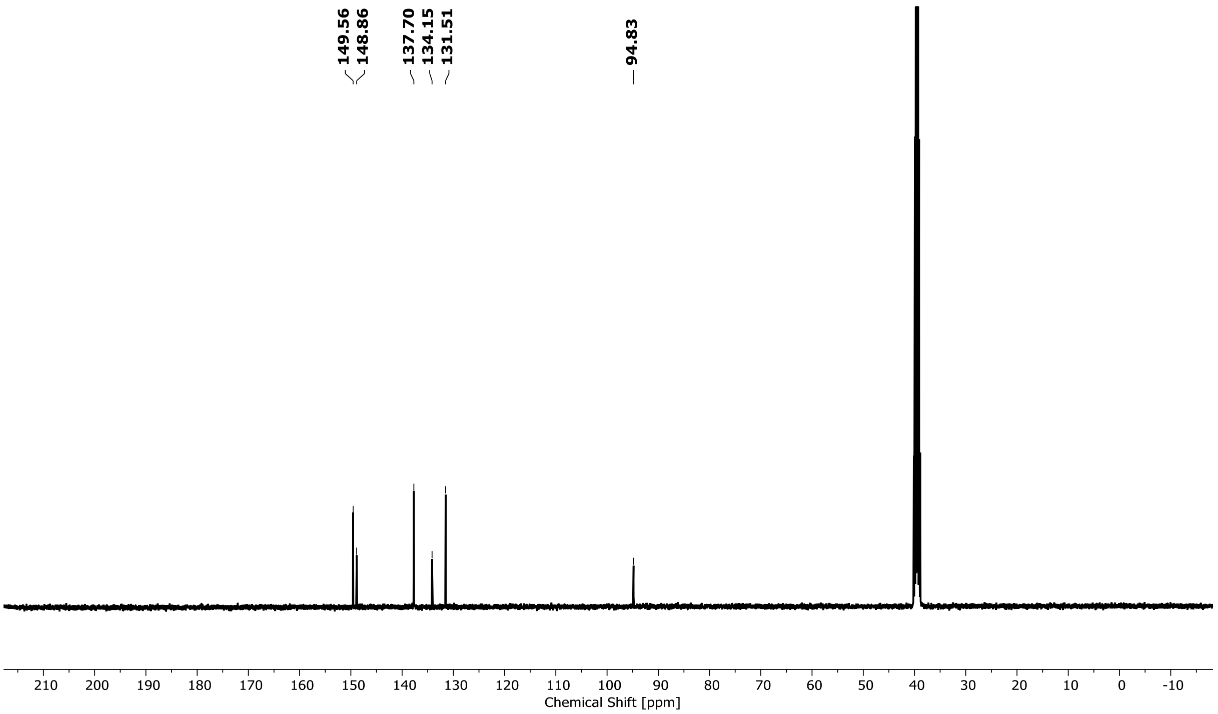

Figure S37. ^{13}C NMR (101 MHz, DMSO-d_6, rt) of **4-Iodo phenyl cyanuric acid**.

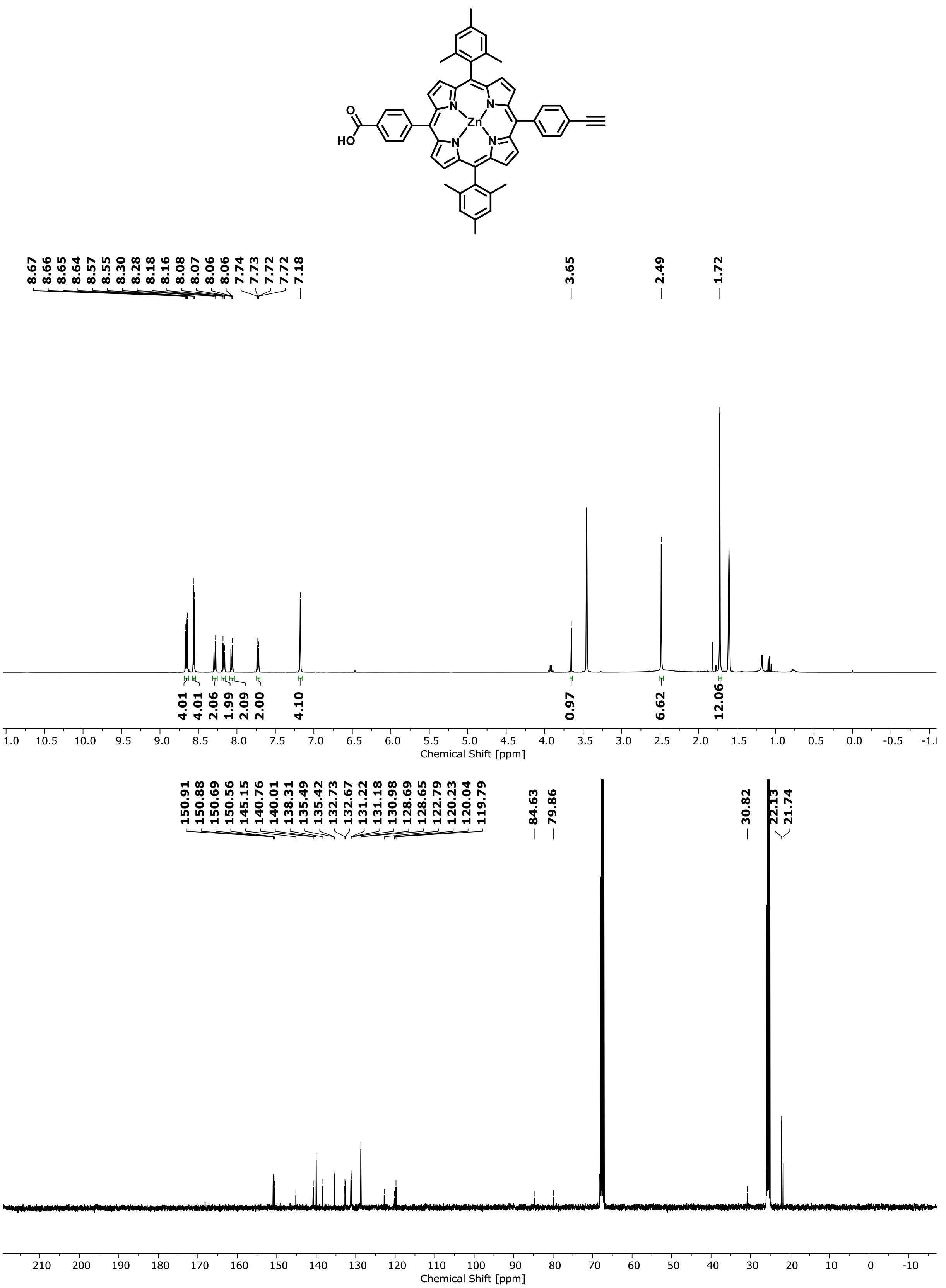

Figure S38. Top: ^{1}H NMR (400 MHz, THF-d_8) of **AcidPor**, Bottom: ^{13}C NMR (101 MHz, THF-d_8) of **AcidPor**.

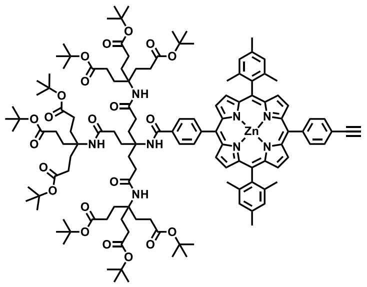

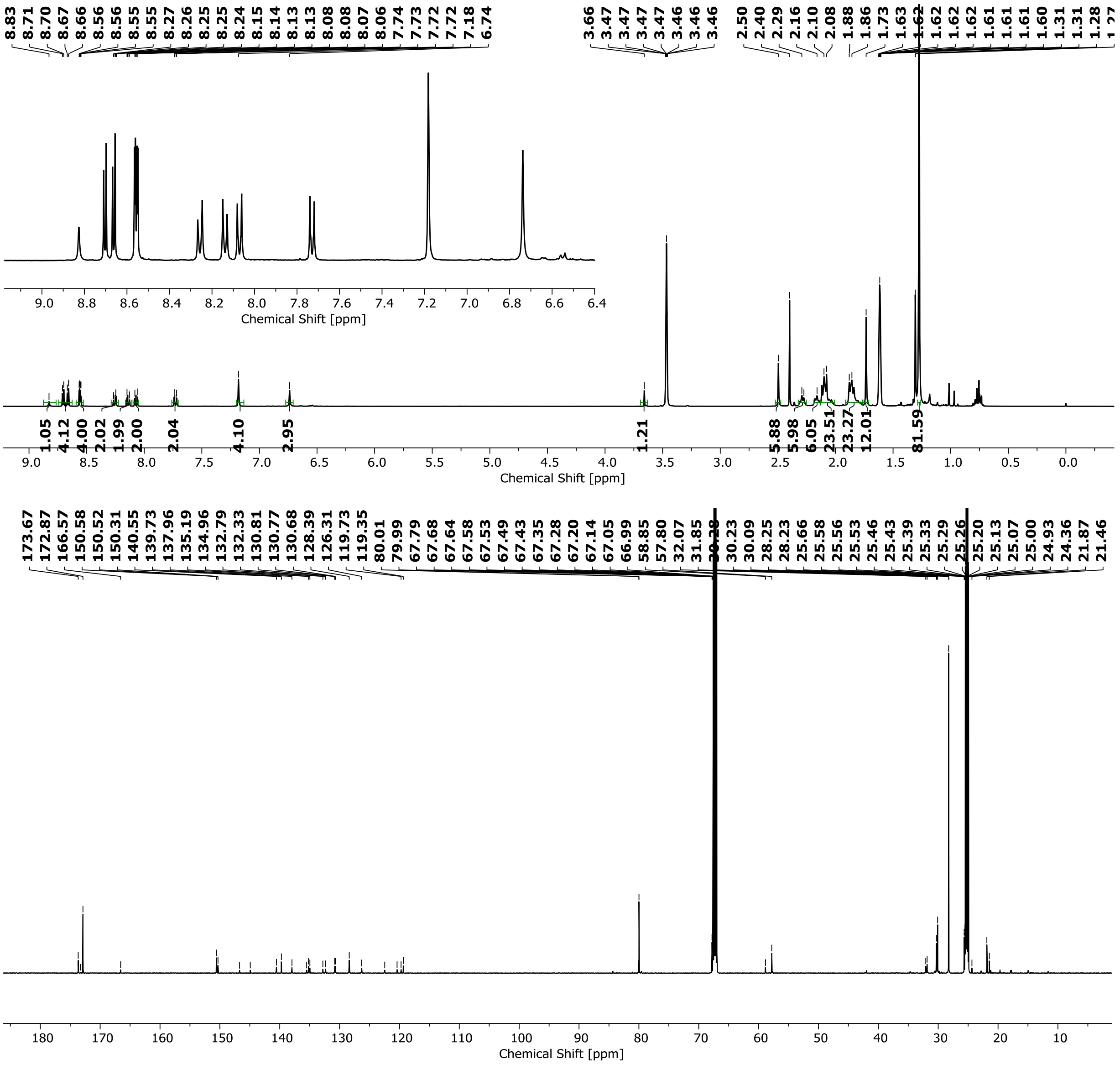

Figure S39. Top: ^{1}H NMR (400 MHz, THF-d_8) of **G2Por**, Bottom: ^{13}C NMR (101 MHz, THF-d_8) of **G2Por**.

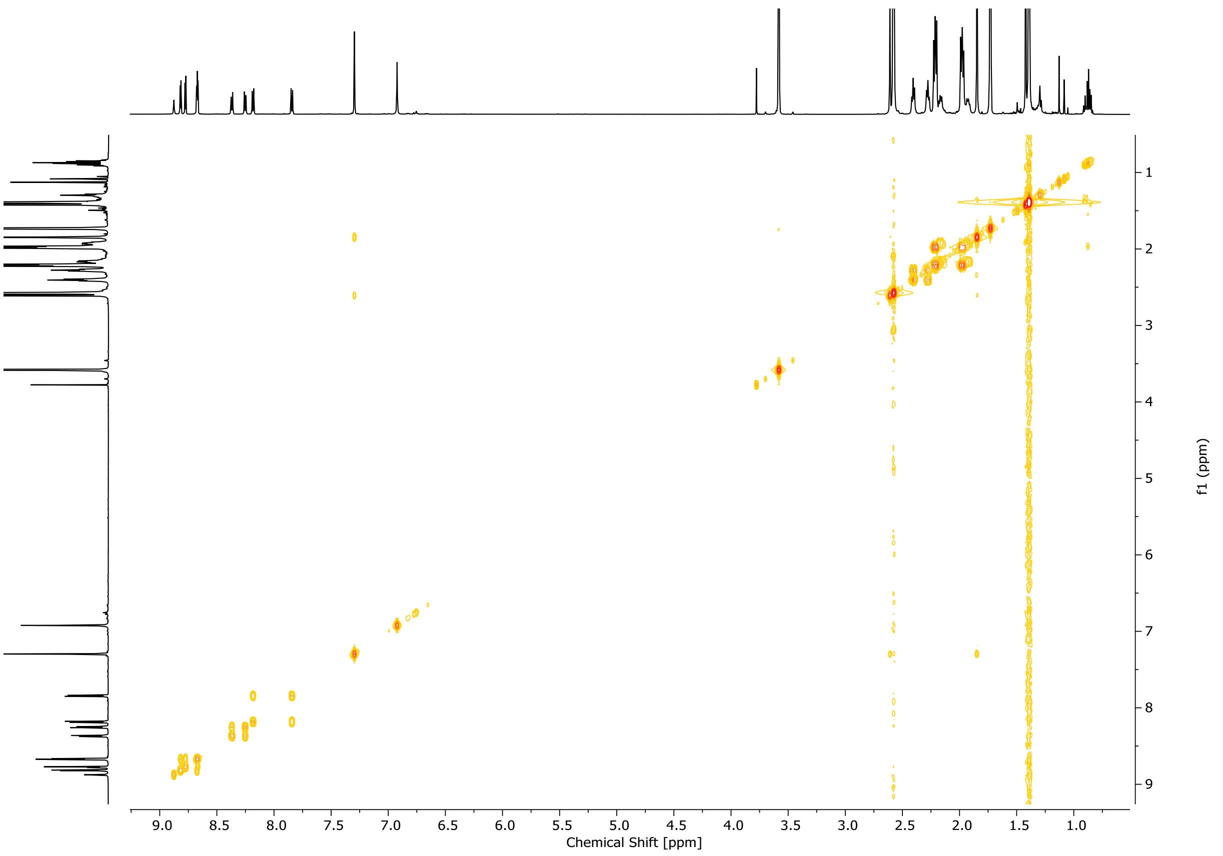

Figure S40. COSY NMR (600 MHz, THF-d_8) of **G2Por**.

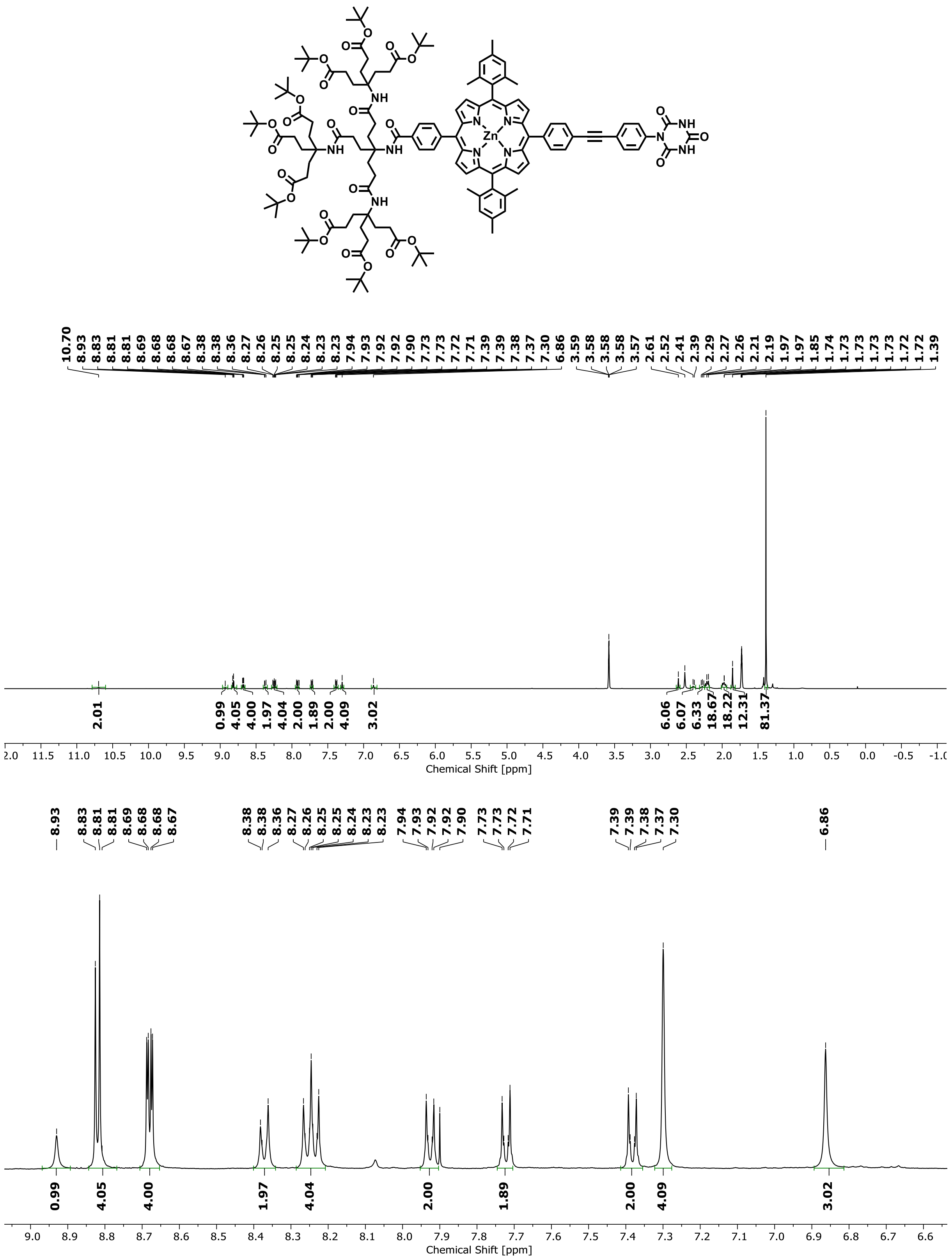

Figure S41. ^{1}H NMR (600 MHz, THF-d8) of ***t*BuCyPor** (Top: whole spectrum; bottom: aromatic region).

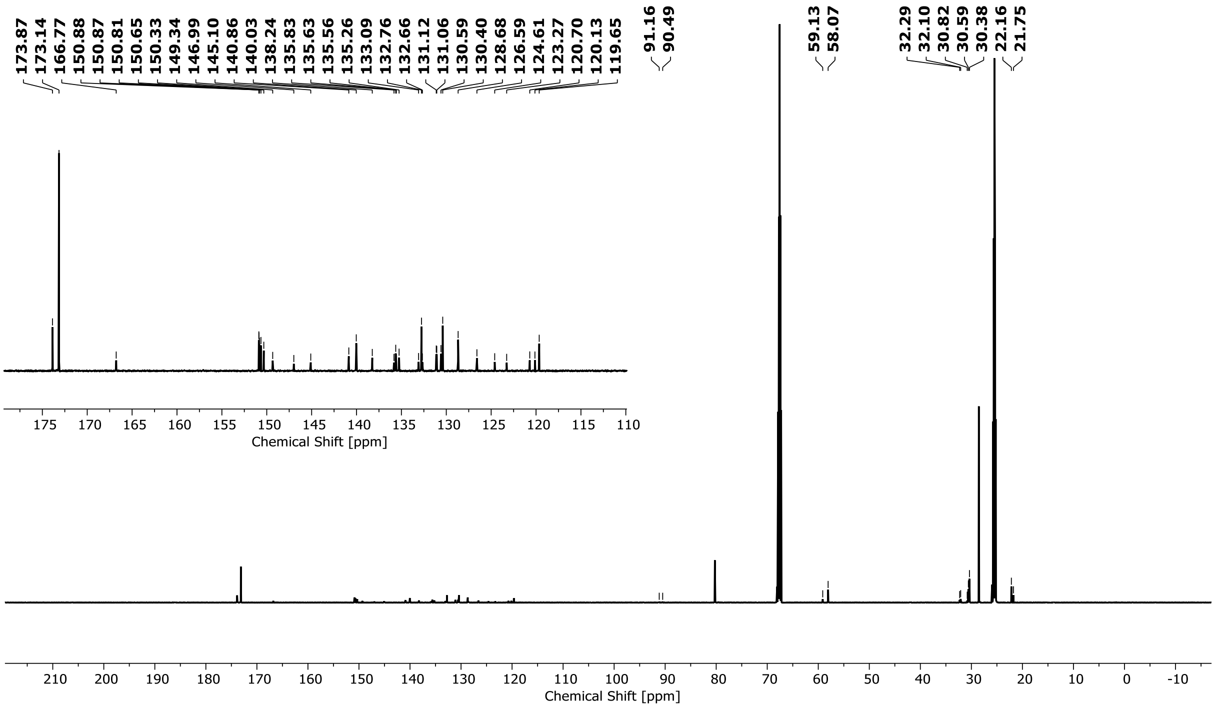

Figure S42. ^{13}C NMR (151 MHz, THF-d_8, rt) of ***t*BuCyPor** (Top: whole spectrum; bottom: aromatic region).

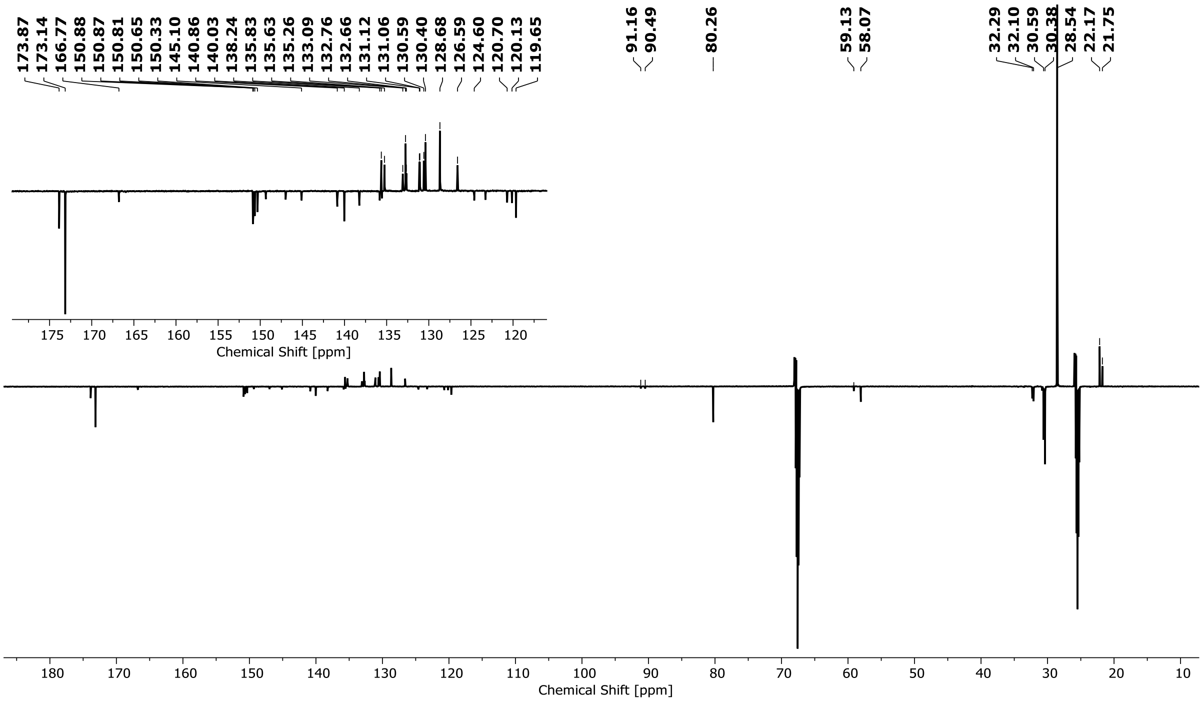

Figure S43. DEPT135 NMR (151 MHz, THF-d8, rt) of ***t*BuCyPor** (Top: whole spectrum; bottom: aromatic region).

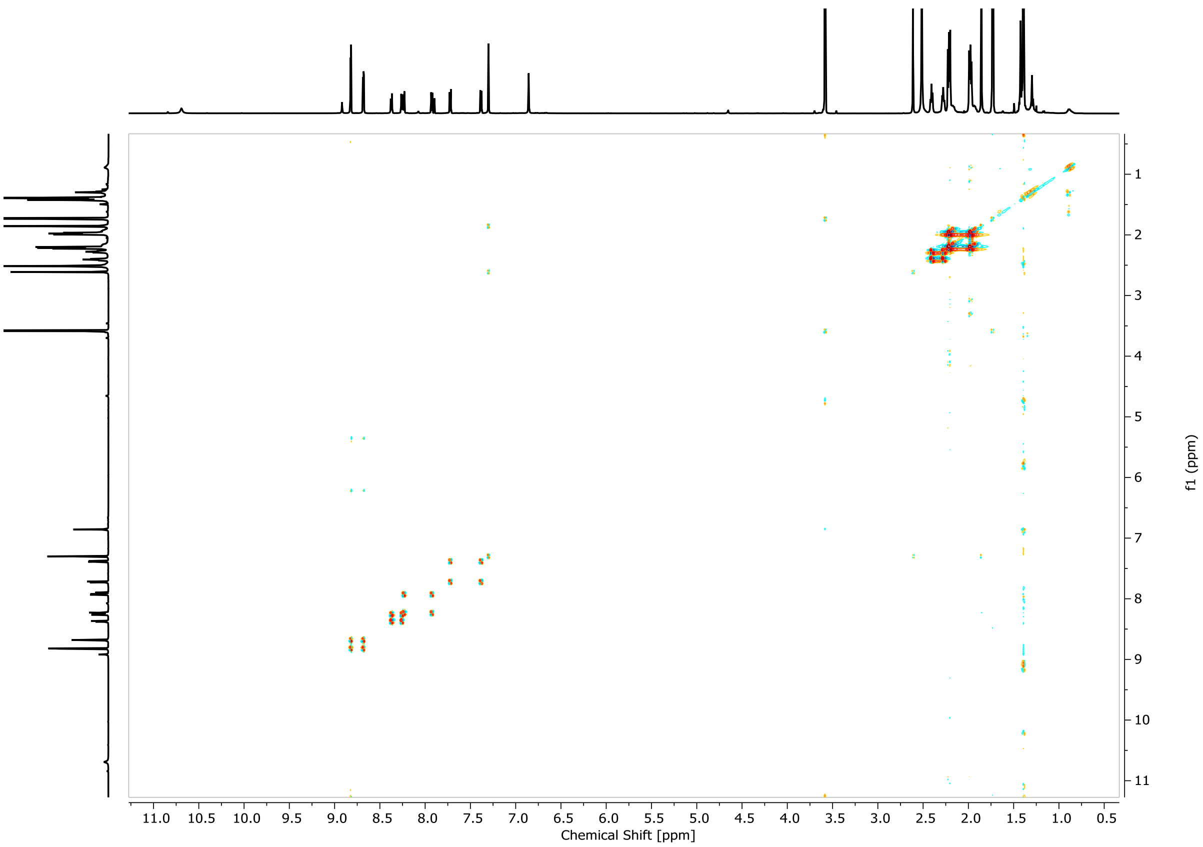

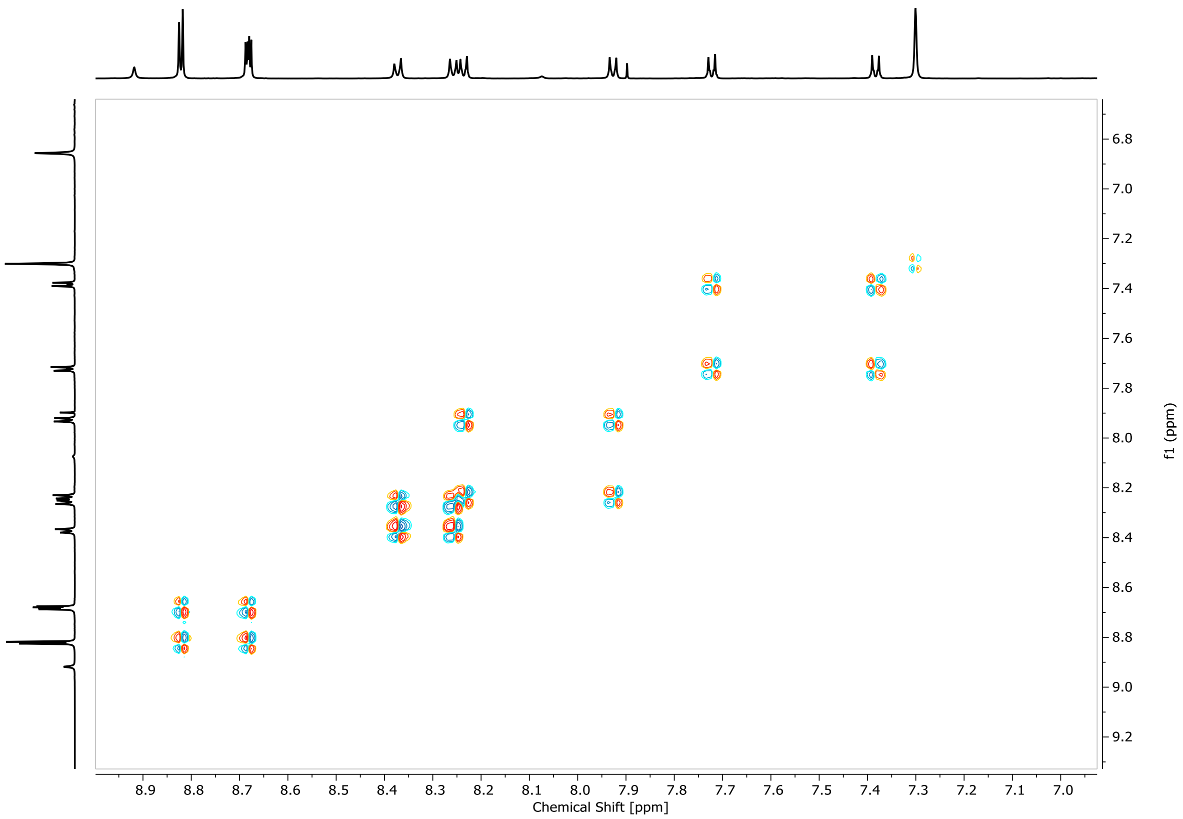

Figure S44. COSY NMR (600 MHz, THF-d_8, rt) of ***t*BuCyPor** (Top: whole spectrum; bottom: aromatic region)**.**

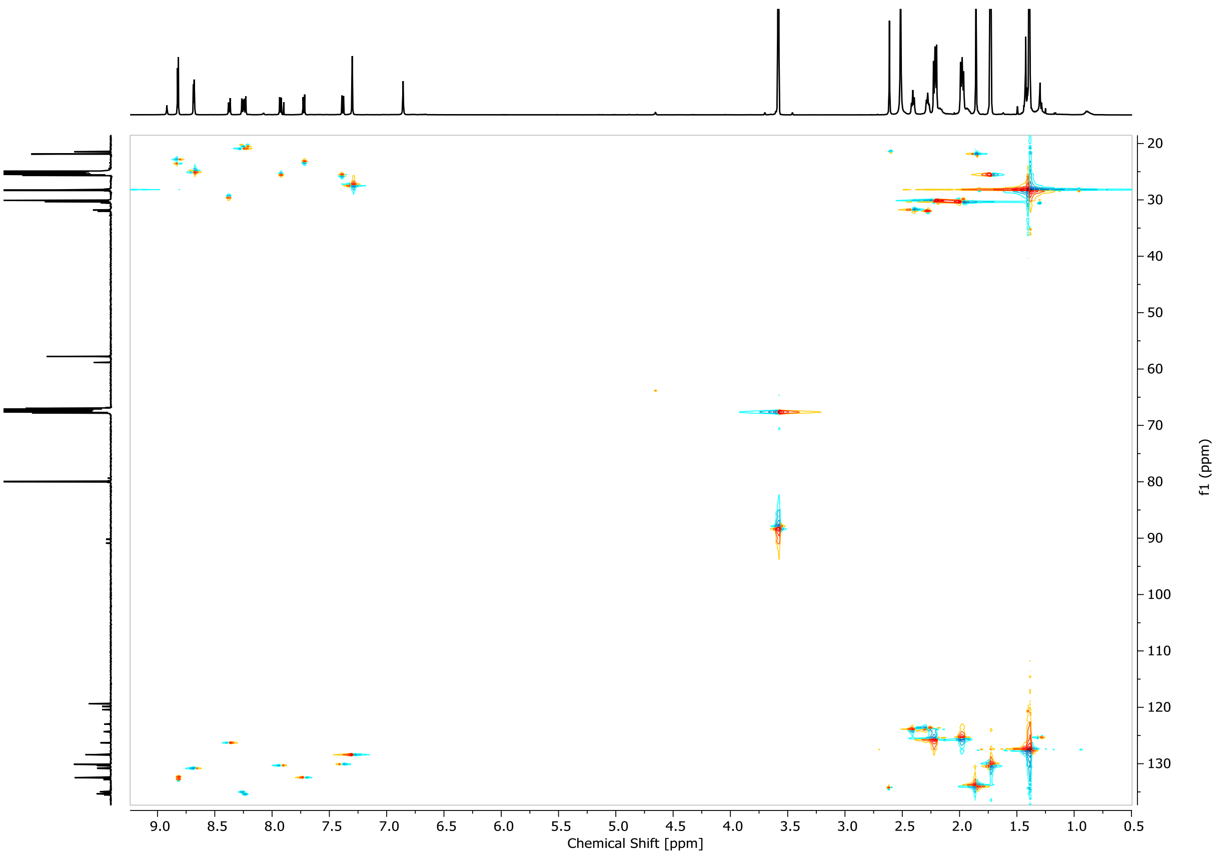

Figure S45. HSQC NMR (600 MHz, THF-d_8, rt) of ***t*BuCyPor**.

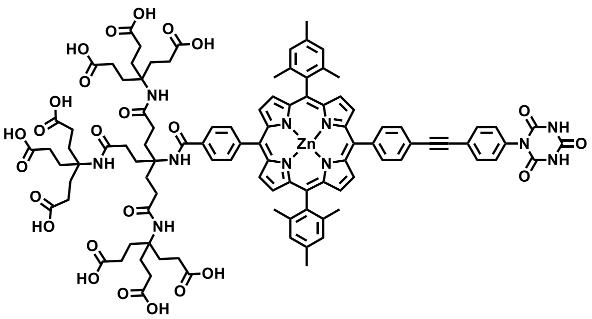

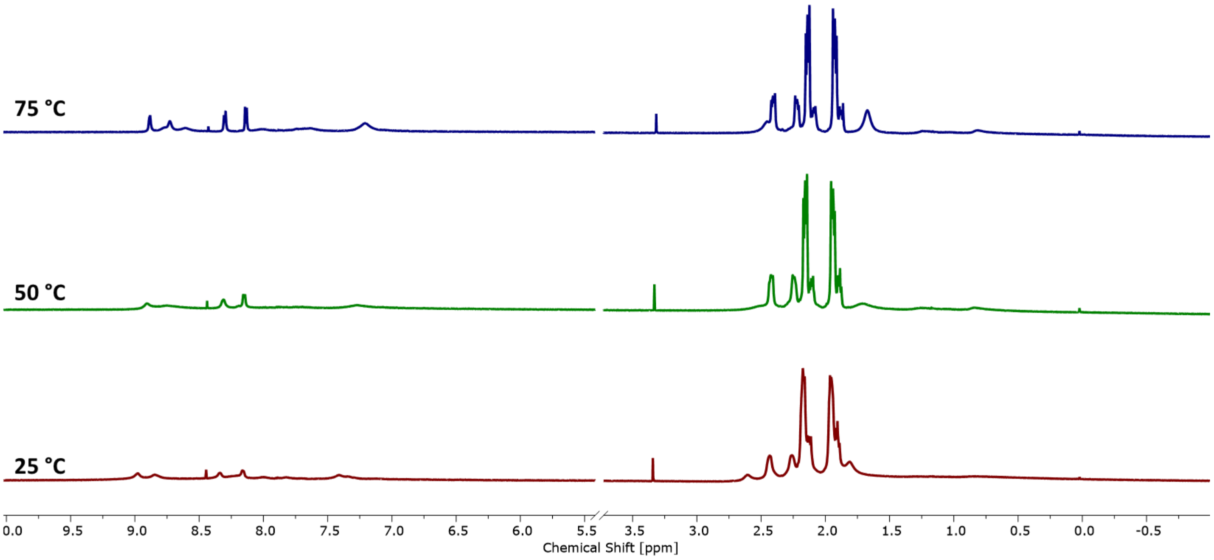

Figure S46. Temperature dependent ^{1}H NMR (600 MHz, D_2O / K_3PO_4) of **CyPor**; the HDO signal was omitted for clarity.

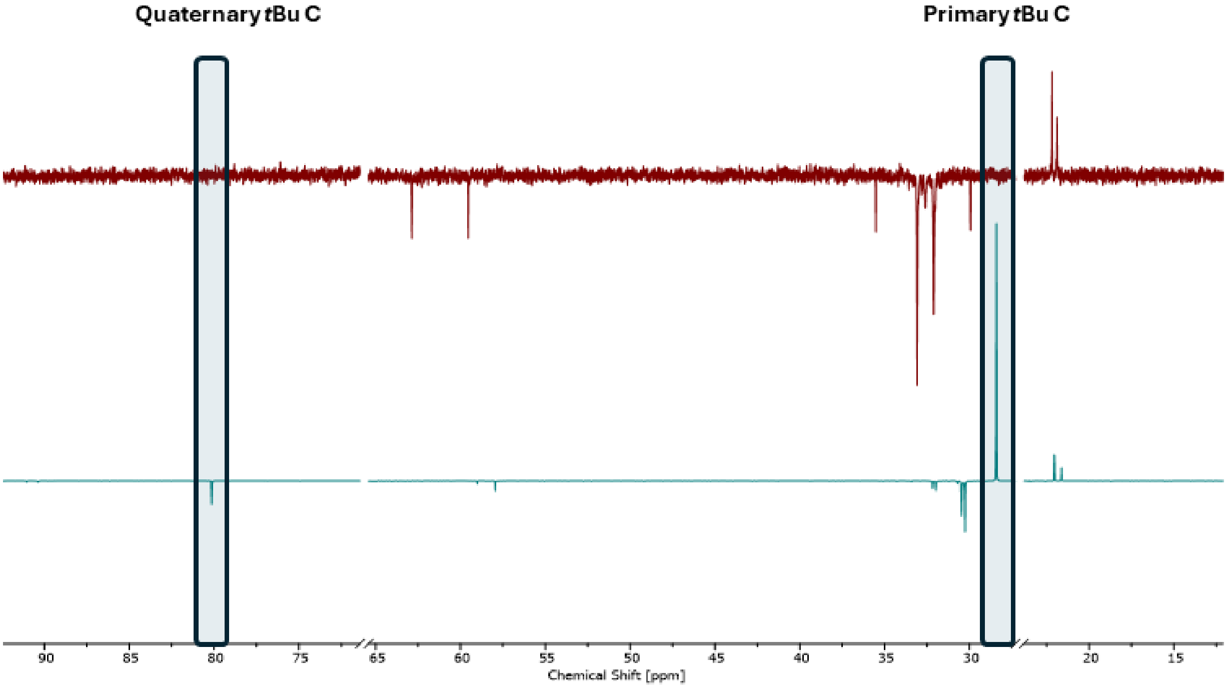

Figure S47. Aliphatic region of the DEPTq 135 NMRs of **CyPor** (red) and ***t*BuCyPor** (turquoise). The absence of the quaternary carbon and primary carbon of the *t*Bu group are discernable.

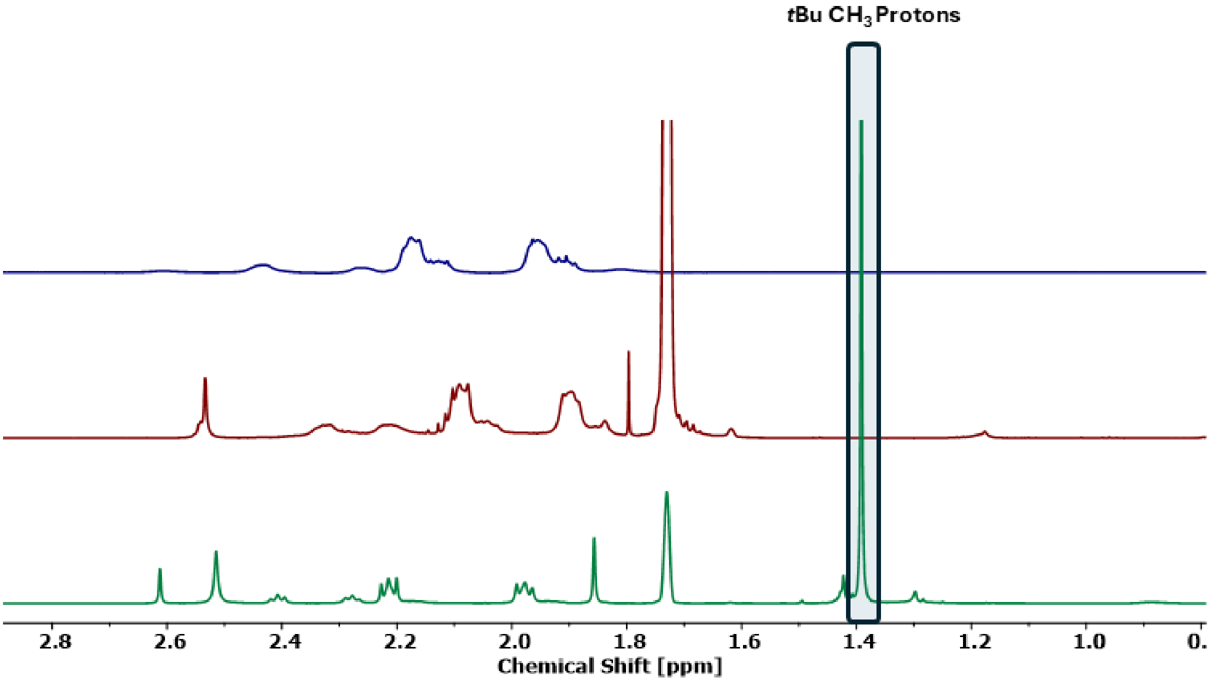

Figure S48. Aliphatic region of the ^{1}H NMRs (600 MHz, rt) of **CyPor** (blue, D_2O / K_3PO_4), **CyPor** (red, THF-d_8 / D_2O 1:1) and ***t*BuCyPor** (green, THF-d_8). The absence of the *t*Bu-protons is clearly discernable for the deprotected amphiphiles.

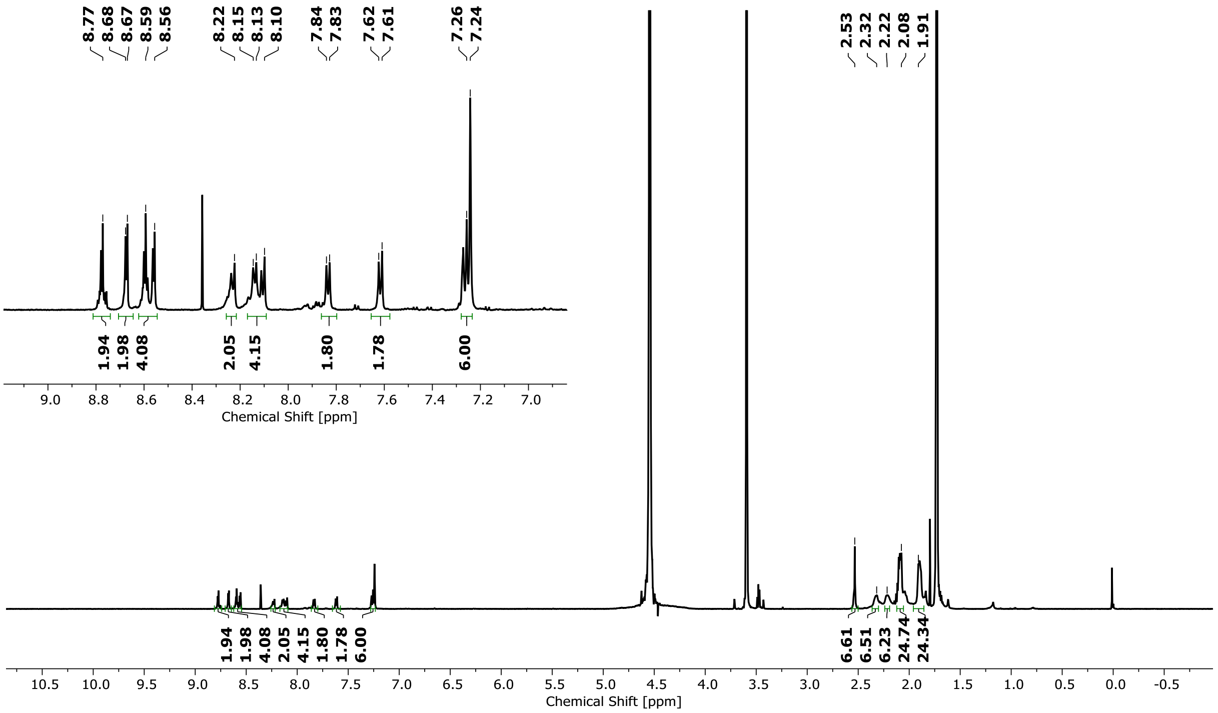

Figure S49. ^{1}H NMR (600 MHz, THF-d_8 / D_2O 1:1, 25 °C) of the sodium ion of **CyPor**.

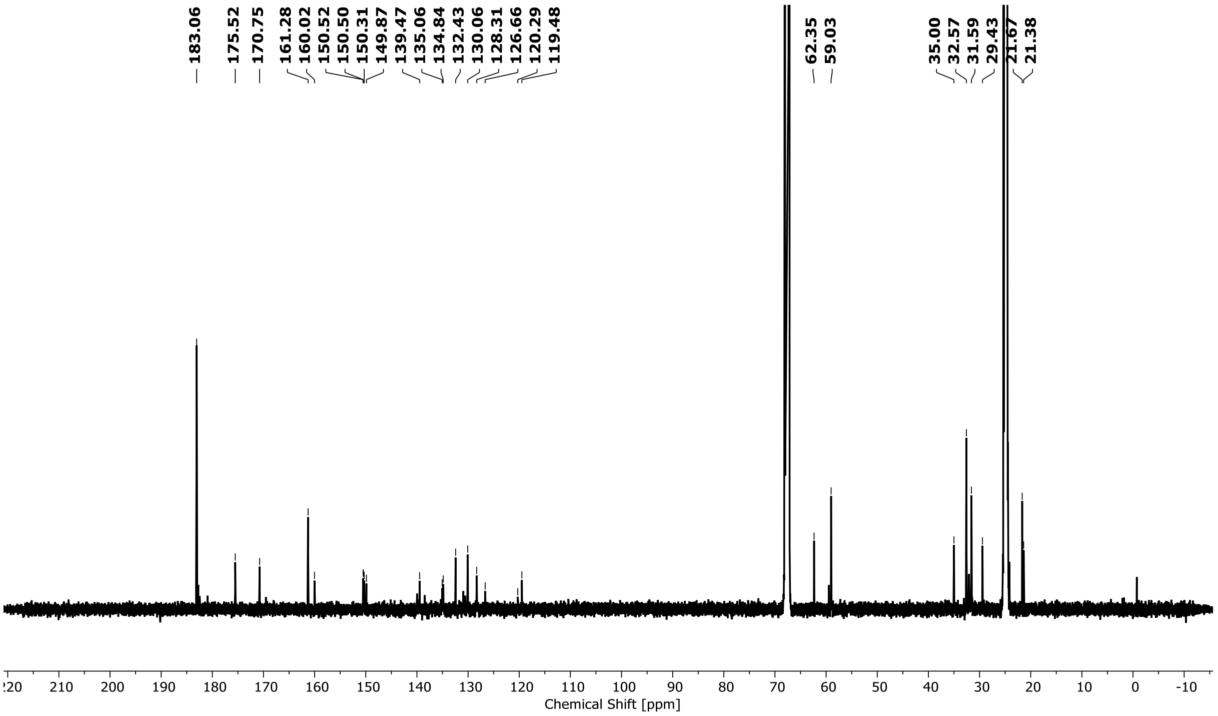

Figure S50. ^{13}C NMR (151 MHz, THF-d_8 / D_2O 1:1, 25 °C) of the sodium ion of **CyPor**.

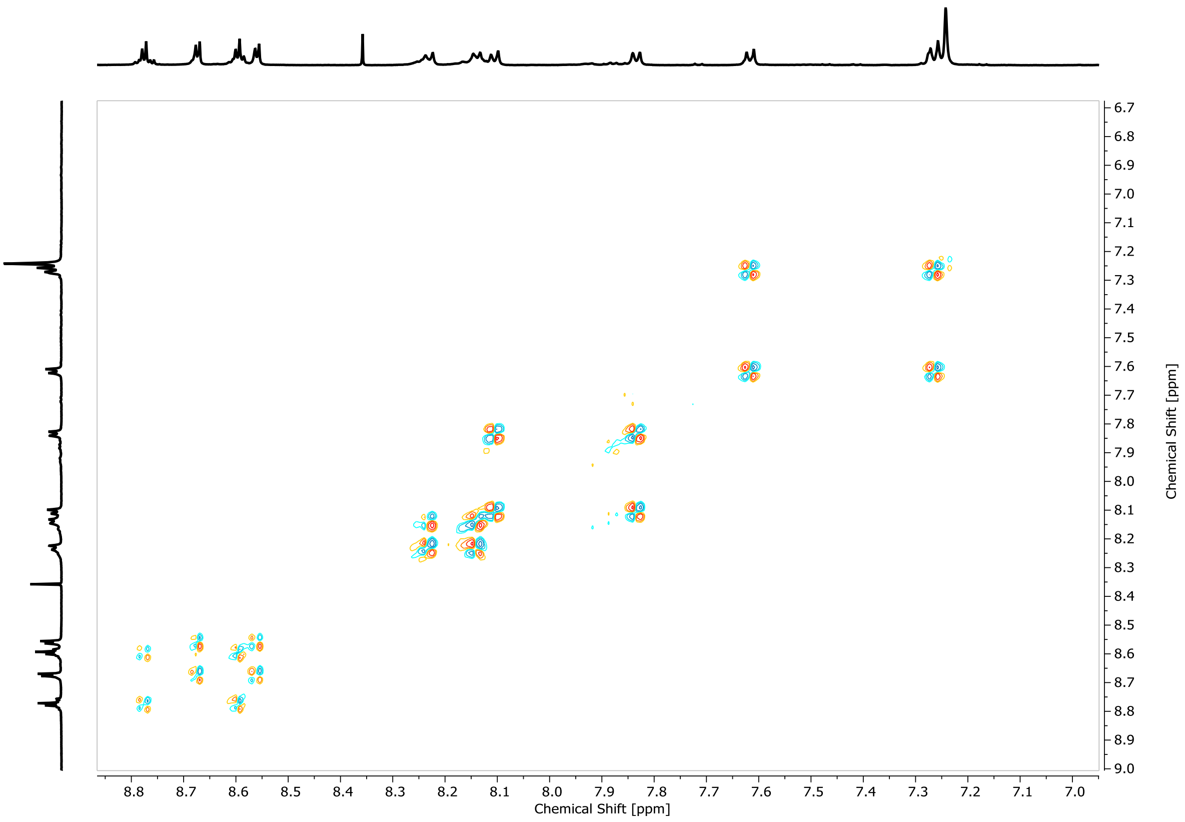

Figure S51. COSY NMR (600MHz THF-d_8 / D_2O 1:1, 25 °C) of the sodium ion of **CyPor**.

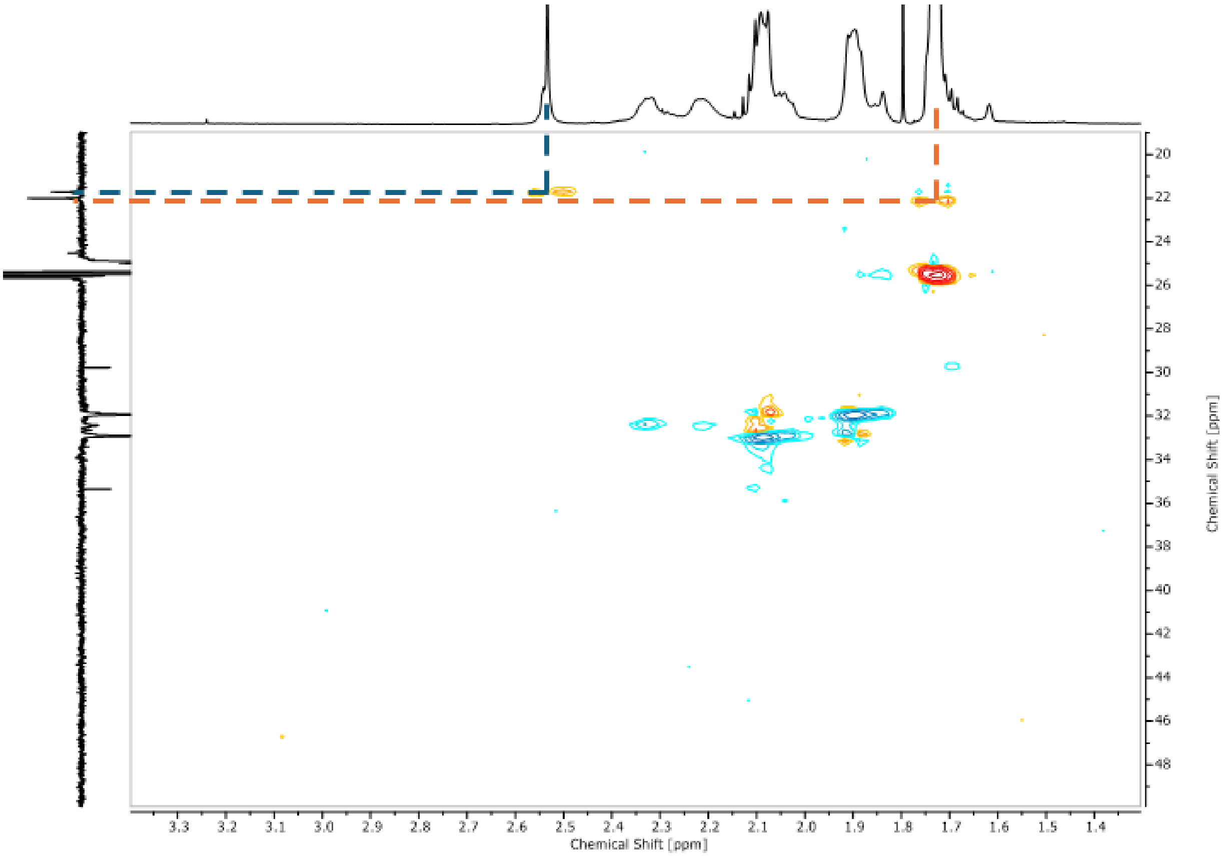

Figure S52. HSQC NMR (THF-d_8/D_2O 1:1, rt) of the sodium ion of **CyPor**.

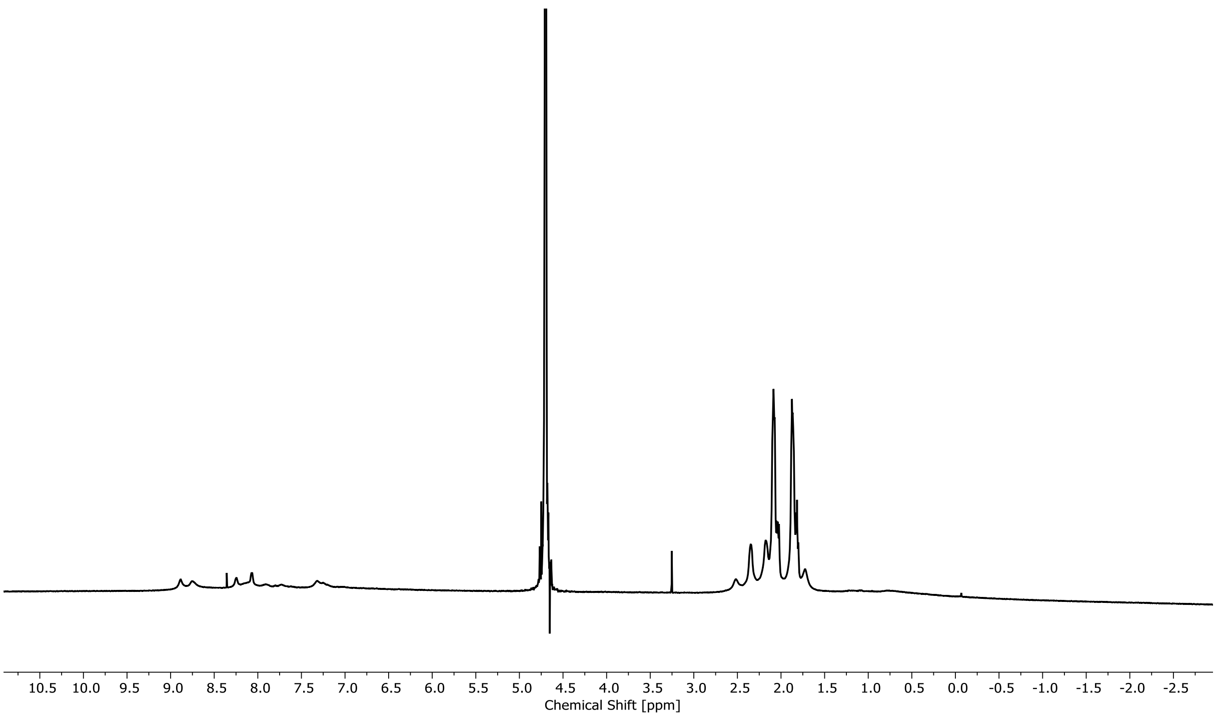

Figure S53. ^{1}H NMR (600 MHz, D_2O / K_3PO_4 25 °C) of **CyPor**.

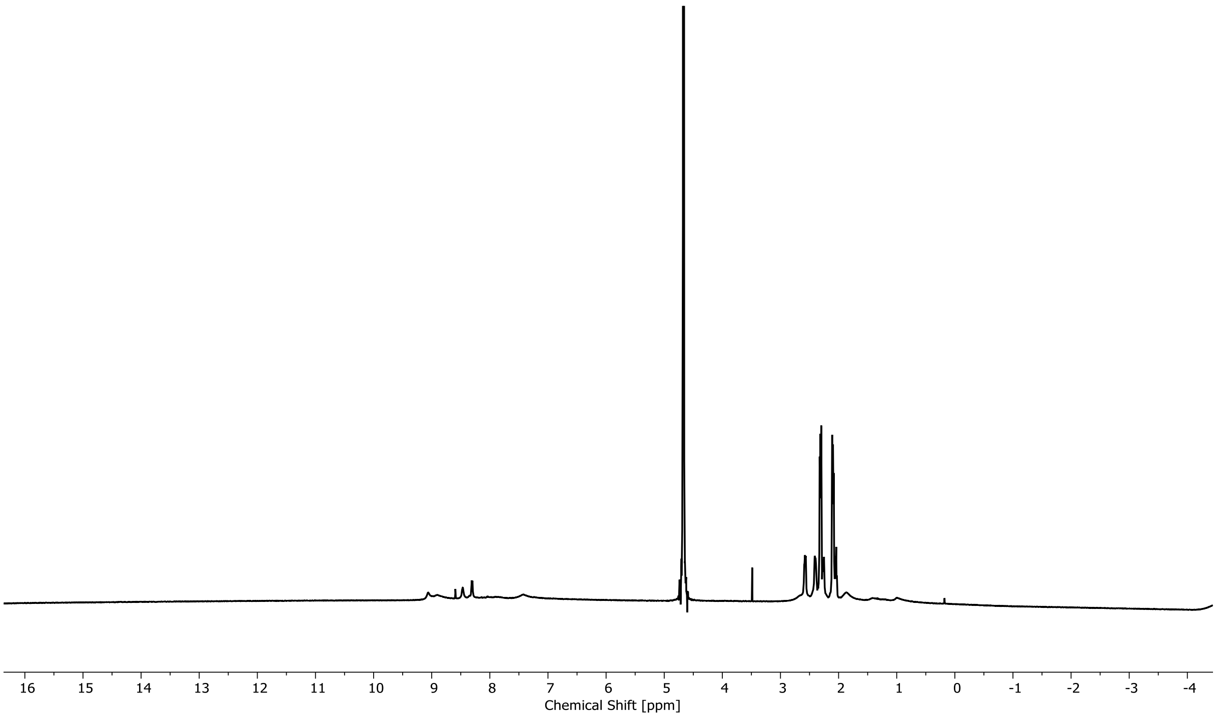

Figure S54. ^{1}H NMR (600 MHz, D_2O / K_3PO_4 50 °C) of **CyPor**.

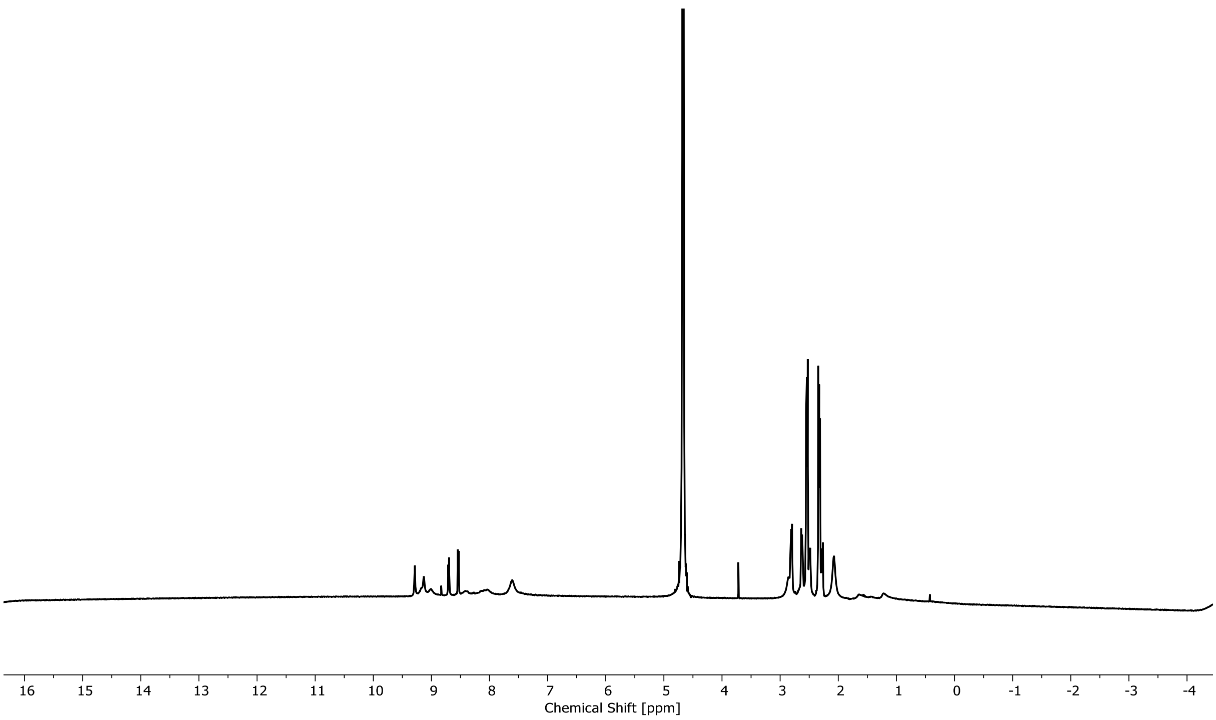

Figure S55. ^{1}H NMR (600 MHz, D_2O / K_3PO_4 75 °C) of **CyPor**.

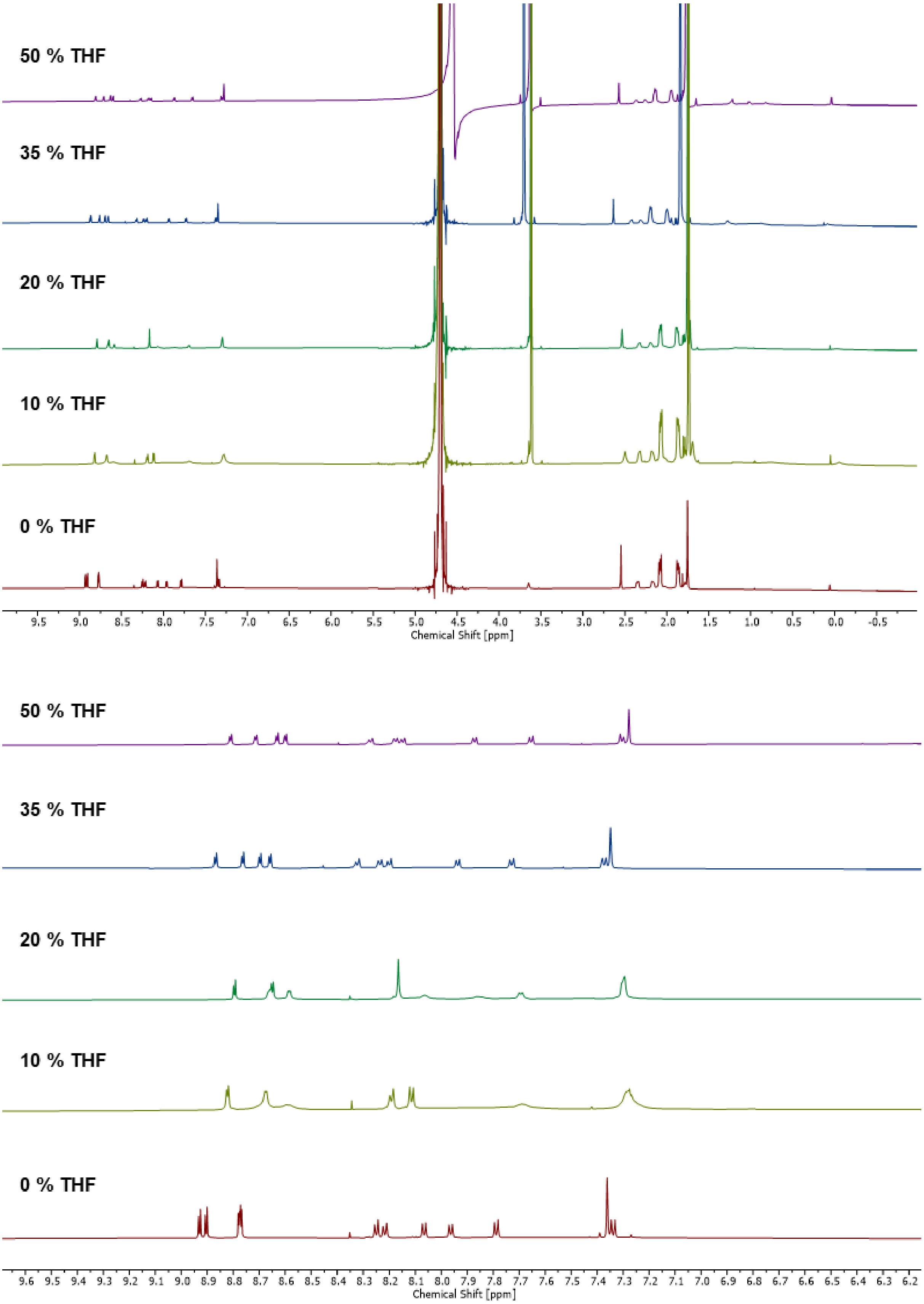

Figure S56. ^{1}H NMR of **BisHam(CyPor)** in D_2O (600 MHz, rt) with different amount of THF-d_8 added. The concentration was not kept constant. Here, only the signals of **CyPor** are observed, even in the "dissolved" state. We reason that at the NMR concentrations upon breaking of the aggregates at low THF amounts **HamPBI** does not stay in solution and precipitates.

Mass Spectrometry

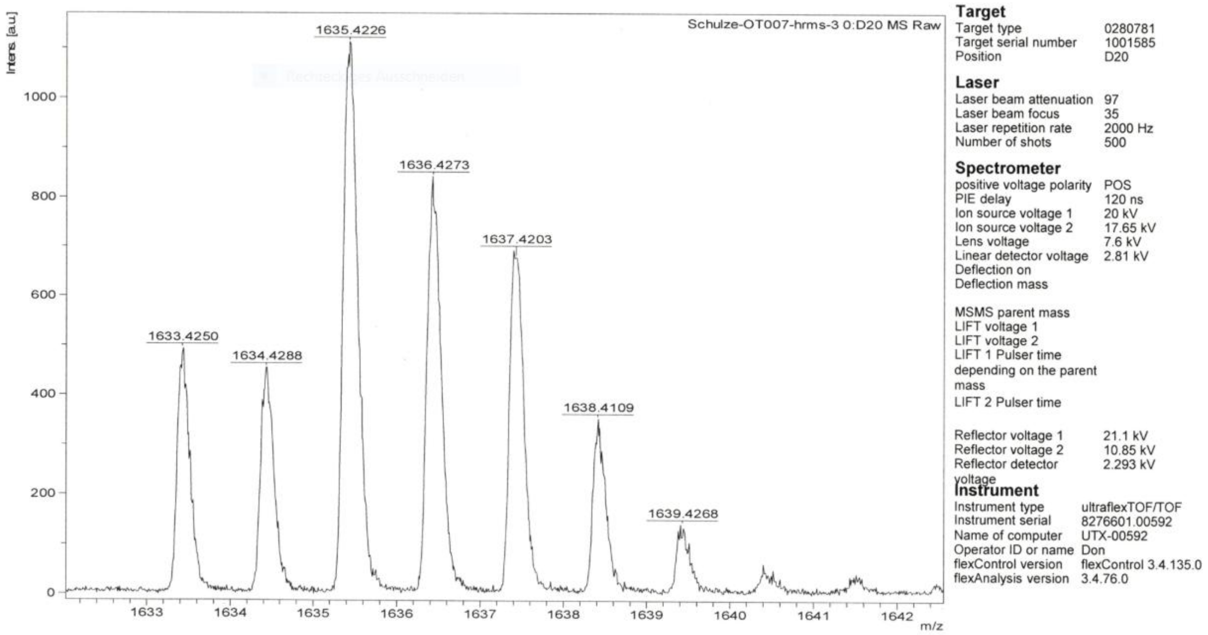

Figure S57. HRMS (MALDI-dctb) of **HamPBI**.

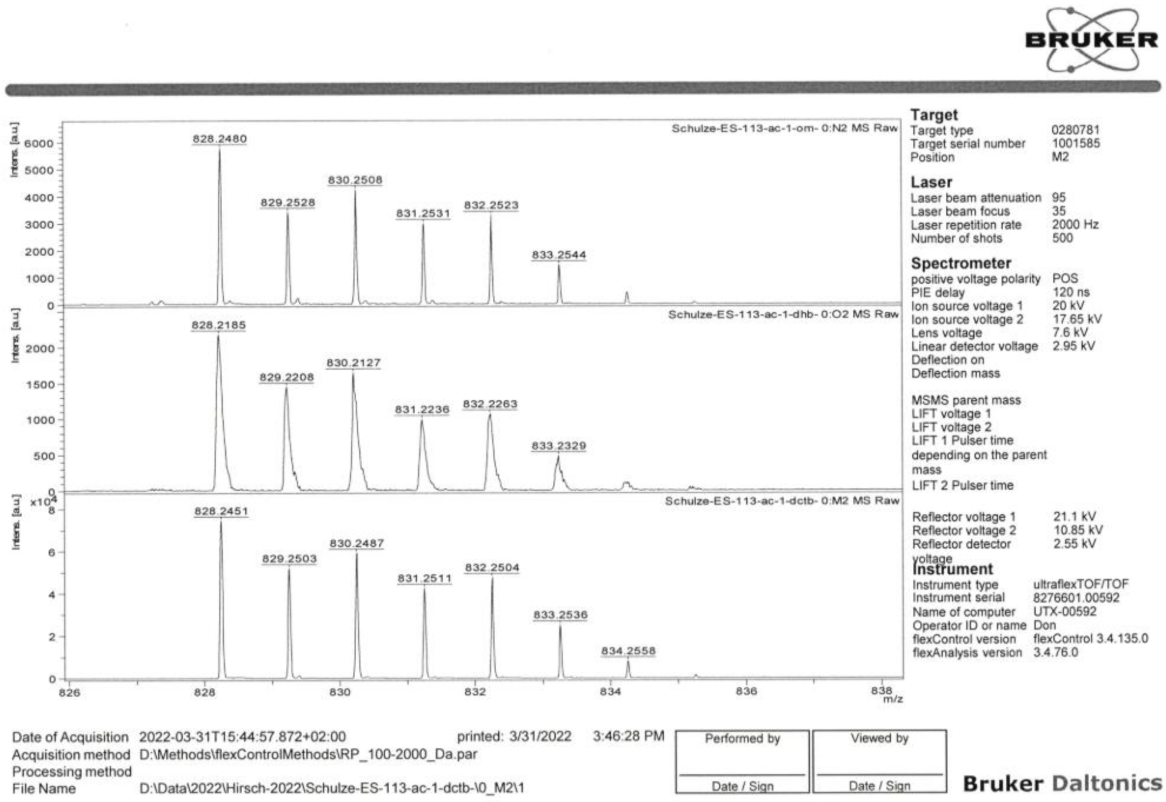

Figure S58. HRMS (MALDI-dctb) of **AcidPor**.

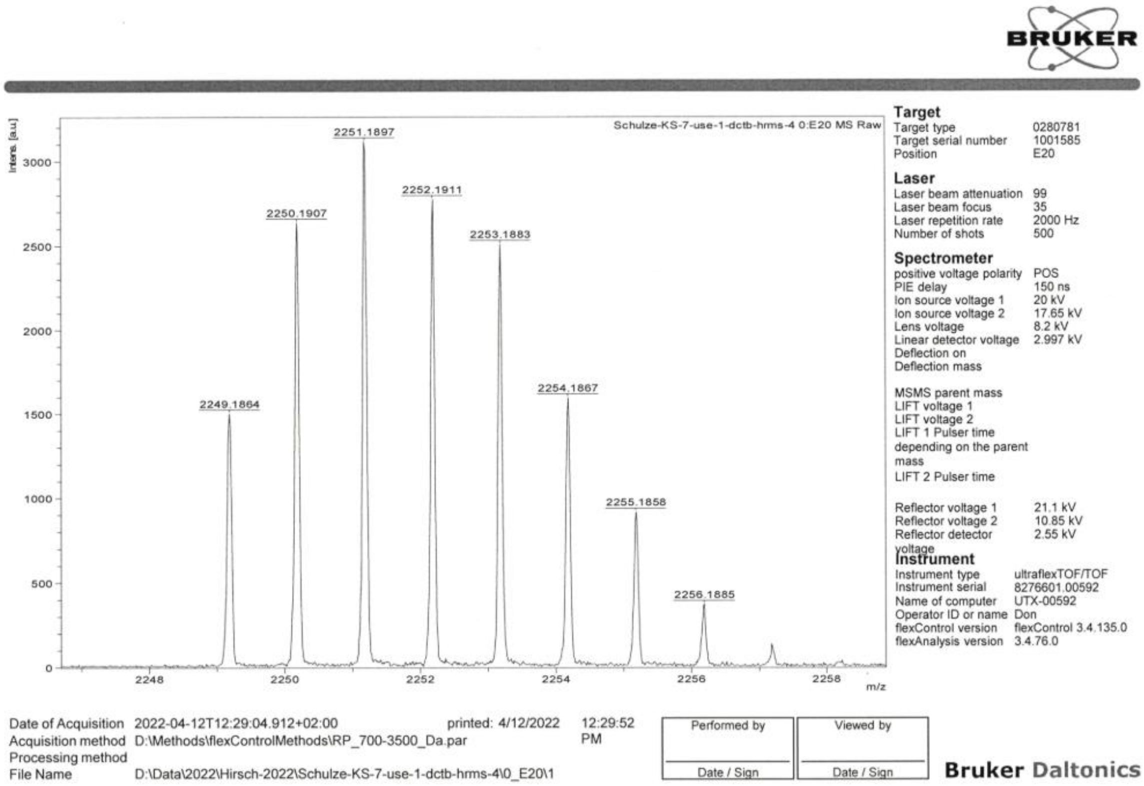

Figure S59. HRMS (MALDI-dctb) of **G2Por**.

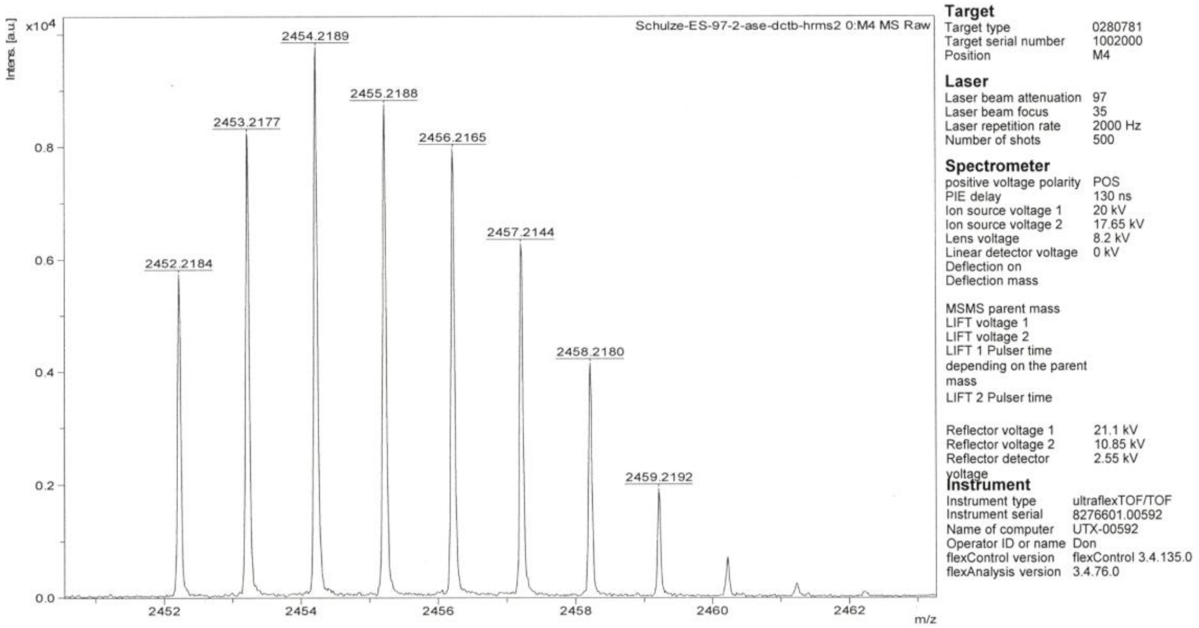

Figure S60. HRMS (MALDI-TOF dctb) of ***t*BuCyPor**.

HRMS of **CyPor** was obtained in ESI negative mode, resulting in the observation of the twofold, threefold and fourfold charge species.

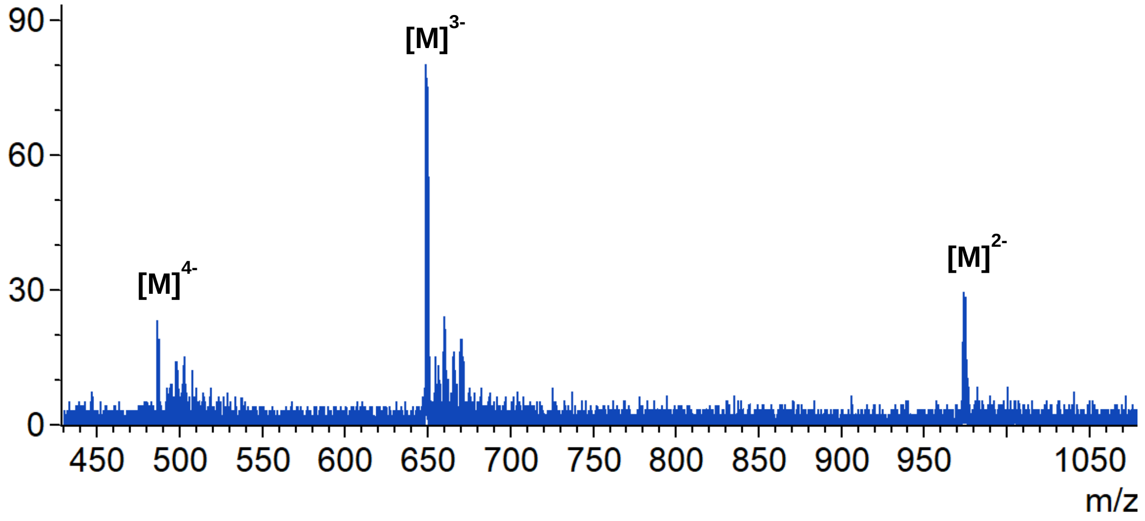

Figure S61. HRMS (ESI-TOF negative mode) of **CyPor**.

Calc.: [M]$^{-}$ ($C_{102}H_{1103}N_{11}O_{25}Zn$): 1946.6502 m/z; found: 1946.6708 m/z

Calc.: [M]$^{2-}$ ($C_{102}H_{1103}N_{11}O_{25}Zn$): 972.8214 m/z; found: 972.8241 m/z

Calc.: [M]$^{3-}$ ($C_{102}H_{1103}N_{11}O_{25}Zn$): 648.2119 m/z; found: 648.2220 m/z

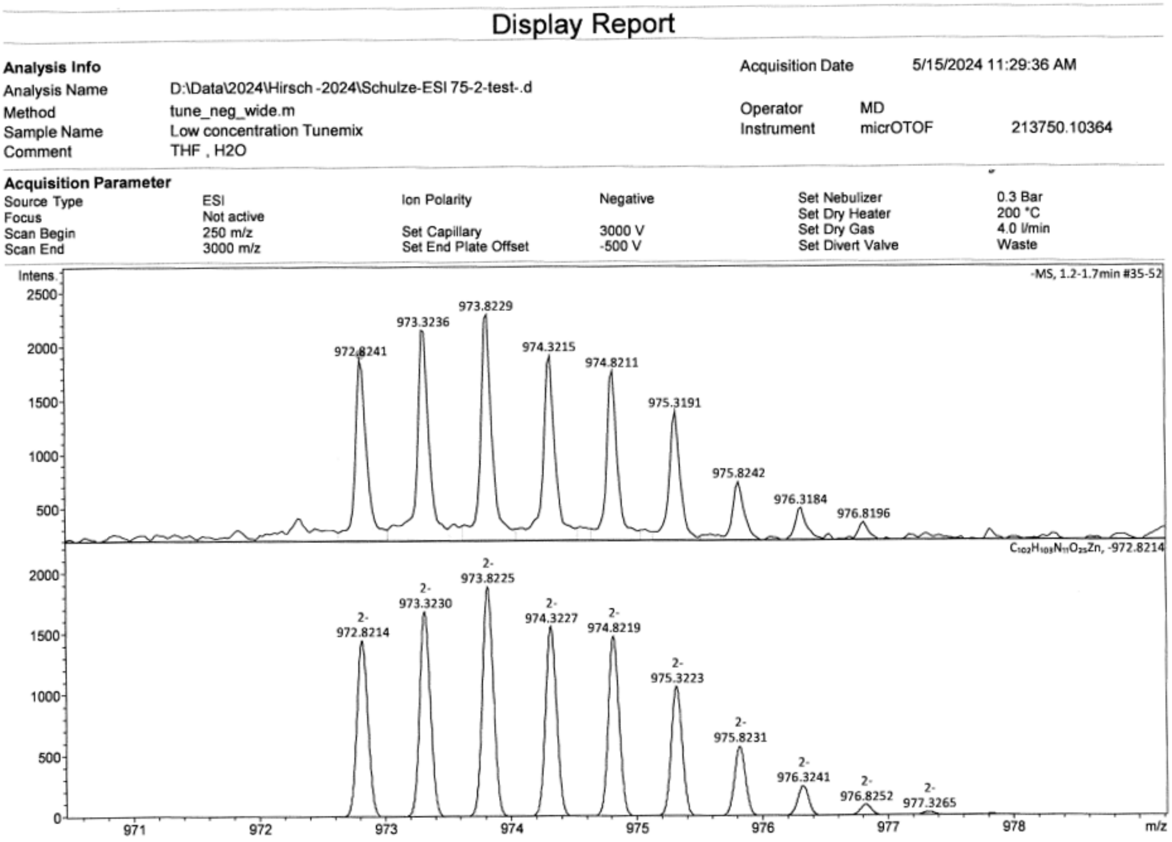

Figure S62. HRMS (ESI-TOF negative mode) of **CyPor**.

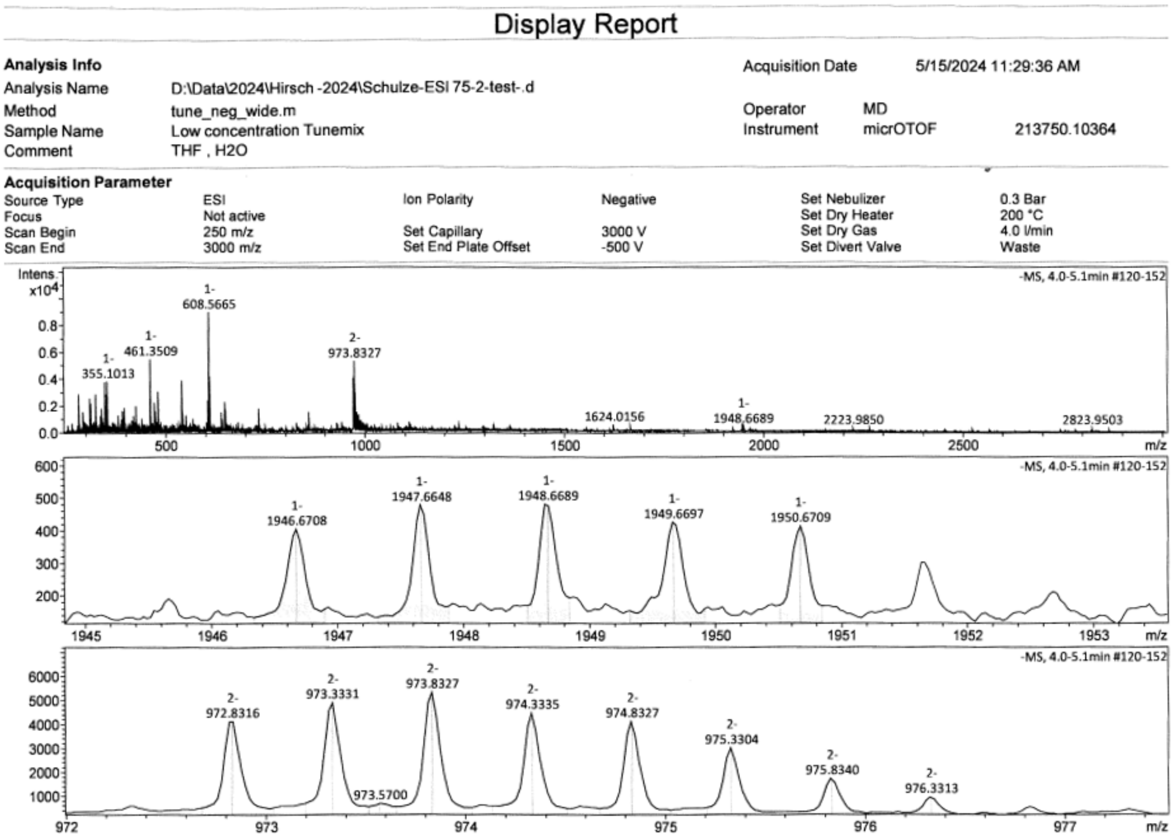

Figure S63. HRMS (ESI-TOF negative mode) of **CyPor** (single and double charged species).

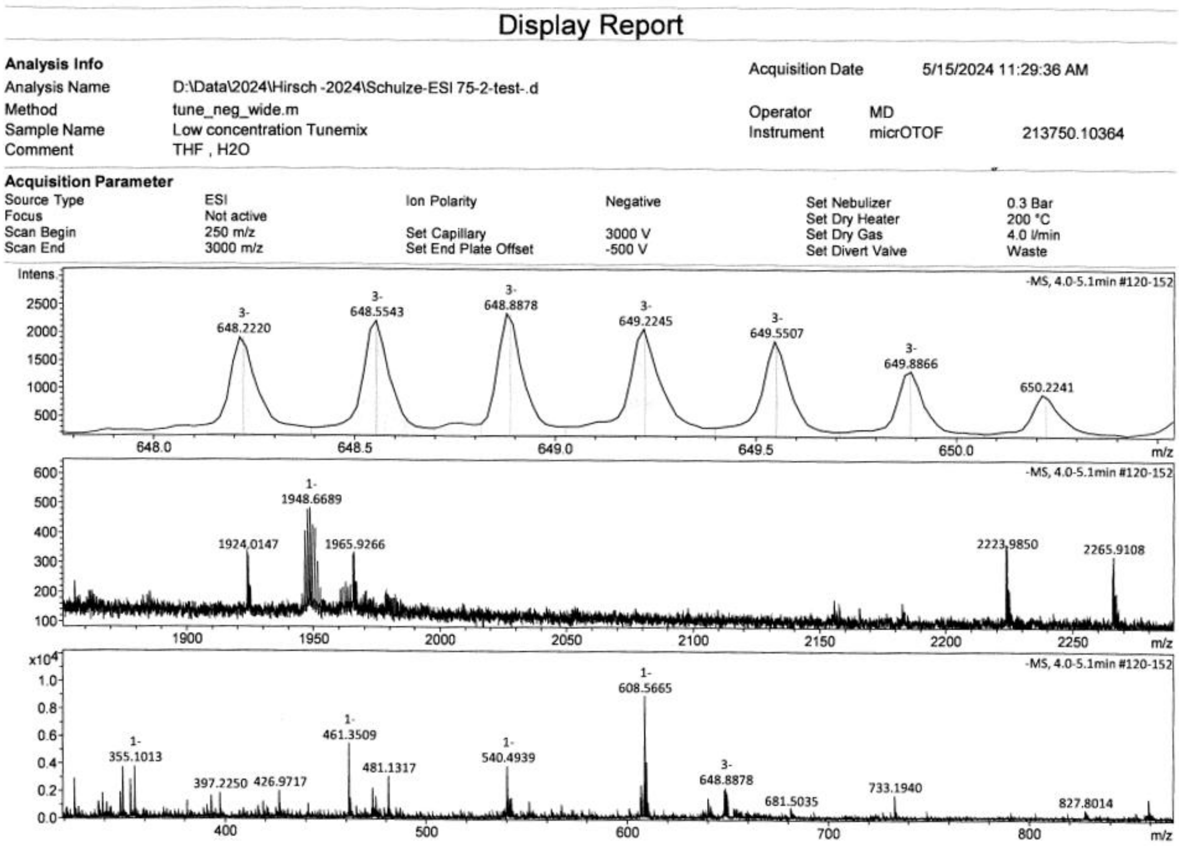

Figure S64. HRMS (ESI-TOF negative mode) of **CyPor** (threefold charged species).

References

[1] J. J. Snellenburg, S. P. Laptenok, R. Seger, K. M. Mullen, I. H. M. van Stokkum, "Glotaran : A Java -Based Graphical User Interface for the R Package TIMP" *J. Stat. Softw.* **2012**, *49*, 1–22.

[2] I. H. M. van Stokkum, D. S. Larsen, R. van Grondelle, "Global and target analysis of time-resolved spectra" *Biochim. Biophys. Acta - Bioenerg.* **2004**, *1657*, 82–104.

[3] M. Ali, E. Kataev, J. Müller, H. Park, M. Halik, A. Hirsch, "Host-Guest Systems on the Surface of Functionalized Superparamagnetic Iron Oxide Nanoparticles (SPIONs) Utilizing Hamilton Receptors and Cyanurate Derivative Molecules" *Chem. - A Eur. J.* **2021**, *27*, 16429–16439.

[4] E. J. Schulze, C. L. Ritterhoff, E. Franz, O. Tavlui, O. Brummel, B. Meyer, A. Hirsch, "Synthesis and Characterization of Bola-Amphiphilic Porphyrin-Perylenebisimide Architectures" *Chem. - A Eur. J.* **2024**, *30*, e202303515.

[5] M. J. Plater, J. P. Sinclair, S. Aiken, T. Gelbrich, M. B. Hursthouse, "The CA.M lattice revisited. Gel formation from a linear bis-isocyanuric acid and 2-amino-4,6-bis-(4-tert-butylphenylamino)-1,3,5-triazine" *Tetrahedron* **2004**, *60*, 6385–6394.

[6] P. Giannozzi, S. Baroni, N. Bonini, M. Calandra, R. Car, C. Cavazzoni, D. Ceresoli, G. L. Chiarotti, M. Cococcioni, I. Dabo, A. Dal Corso, S. De Gironcoli, S. Fabris, G. Fratesi, R. Gebauer, U. Gerstmann, C. Gougoussis, A. Kokalj, M. Lazzeri, L. Martin-Samos, N. Marzari, F. Mauri, R. Mazzarello, S. Paolini, A. Pasquarello, L. Paulatto, C. Sbraccia, S. Scandolo, G. Sclauzero, A. P. Seitsonen, A. Smogunov, P. Umari, R. M. Wentzcovitch, "QUANTUM ESPRESSO: A modular and open-source software project for quantum simulations of materials" *J. Phys. Condens. Matter* **2009**, *21*, 395502.

[7] J. P. Perdew, K. Burke, M. Ernzerhof, "Generalized Gradient Approximation Made Simple" *Phys. Rev. Lett.* **1996**, *77*, 3865–3868.

[8] S. Grimme, J. Antony, S. Ehrlich, H. Krieg, "A consistent and accurate ab initio parametrization of density functional dispersion correction (DFT-D) for the 94 elements H-Pu" *J. Chem. Phys.* **2010**, *132*, DOI 10.1063/1.3382344.

[9] S. Grimme, S. Ehrlich, L. Goerigk, "Effect of the damping function in dispersion corrected density functional theory" *J. Comput. Chem.* **2011**, *32*, 1456–1465.

[10] D. Vanderbilt, "Soft self-consistent pseudopotentials in a generalized eigenvalue formalism" *Phys. Rev. B* **1990**, *41*, 7892–7895.

[11] F. Neese, "The ORCA program system" *WIREs Comput. Mol. Sci.* **2012**, *2*, 73–78.

[12] C. Lee, W. Yang, R. G. Parr, "Development of the Colle-Salvetti correlation-energy formula into a functional of the electron density" *Phys. Rev. B* **1988**, *37*, 785–789.

[13] A. D. Becke, "A new mixing of Hartree-Fock and local density-functional theories" *J. Chem. Phys.* **1993**, *98*, 1372–1377.

[14] F. Weigend, R. Ahlrichs, "Balanced basis sets of split valence, triple zeta valence and quadruple zeta valence quality for H to Rn: Design and assessment of accuracy" *Phys. Chem. Chem. Phys.* **2005**, *7*, 3297.

[15] F. Neese, F. Wennmohs, A. Hansen, U. Becker, "Efficient, approximate and parallel Hartree-Fock and hybrid DFT calculations. A “chain-of-spheres” algorithm for the Hartree-Fock exchange" *Chem. Phys.* **2009**, *356*, 98–109.

[16] T. Yanai, D. P. Tew, N. C. Handy, "A new hybrid exchange-correlation functional using the Coulomb-attenuating method (CAM-B3LYP)" *Chem. Phys. Lett.* **2004**, *393*, 51–57.
